# Supplementary material for: Identification of biomarkers that predict response to subthalamic nucleus deep brain stimulation in resistant obsessive–compulsive disorder: protocol for an open-label follow-up study
Source: BMJ Open. 2021 Jun 21;11(6):e047492. doi: 10.1136/bmjopen-2020-047492 (PMC8220486; doi:10.1136/bmjopen-2020-047492)
Supplement: Author's manuscript [file bmjopen-2020-047492.draft_revisions.pdf]

BMJ Open

BMJ Open is committed to open peer review. As part of this commitment we make the peer review history of every article we publish publicly available.

When an article is published we post the peer reviewers' comments and the authors' responses online. We also post the versions of the paper that were used during peer review. These are the versions that the peer review comments apply to.

The versions of the paper that follow are the versions that were submitted during the peer review process. They are not the versions of record or the final published versions. They should not be cited or distributed as the published version of this manuscript.

BMJ Open is an open access journal and the full, final, typeset and author-corrected version of record of the manuscript is available on our site with no access controls, subscription charges or pay-per-view fees (http://bmjopen.bmj.com).

If you have any questions on BMJ Open's open peer review process please email info.bmjopen@bmj.com

BMJ Open

Identification of biomarkers that predict response to deep brain stimulation over the subthalamic nucleus in obsessive-compulsive disorder

Journal:	*BMJ Open*
Manuscript ID	bmjopen-2020-047492
Article Type:	Protocol
Date Submitted by the Author:	02-Dec-2020
Complete List of Authors:	Arumugham, Shyam Sundar; National Institute of Mental Health and Neuro Sciences, Psychiatry<br>Srinivas, Dwarakanath; National Institute of Mental Health and Neuro Sciences, Neurosurgery<br>Narayanaswamy, Janardhanan ; National Institute of Mental Health and Neuro Sciences, Psychiatry<br>Jaisoorya, TS; National Institute of Mental Health and Neuro Sciences, Psychiatry<br>Kashyap, Himani; National Institute of Mental Health and Neuro Sciences, Clinical Psychology<br>Domenech, Philippe; Hôpitaux Universitaires Henri Mondor, Univ Paris-Est Créteil, DMU CARE - Département Médical-Universitaire de Chirurgie et Anesthésie réanimation, DMU IMPACT, Département Médical-Universitaire de Psychiatrie et d'Addictologie; INSERM U955, Univ of Paris 12 UPEC, Faculté de médecine<br>Palfi, Stéphane; Hôpitaux Universitaires Henri Mondor, Univ Paris-Est Créteil, DMU CARE - Département Médical-Universitaire de Chirurgie et Anesthésie réanimation, DMU IMPACT, Département Médical-Universitaire de Psychiatrie et d'Addictologie; INSERM U955, Univ of Paris 12 UPEC, Faculté de médecine<br>Mallet, Luc; Sorbonne Université, Institut du Cerveau, ICM, Inserm U 1127, CNRS UMR 7225; University of Geneva, Department of Mental Health and Psychiatry<br>Venkatasubramanian, Ganesan; National Institute of Mental Health and Neuro Sciences, Psychiatry<br>Reddy, YC Janardhan; National Institute of Mental Health and Neuro Sciences, Psychiatry
Keywords:	PSYCHIATRY, NEUROSURGERY, Anxiety disorders < PSYCHIATRY

SCHOLARONE™
Manuscripts

I, the Submitting Author has the right to grant and does grant on behalf of all authors of the Work (as defined in the below author licence), an exclusive licence and/or a non-exclusive licence for contributions from authors who are: i) UK Crown employees; ii) where BMJ has agreed a CC-BY licence shall apply, and/or iii) in accordance with the terms applicable for US Federal Government officers or employees acting as part of their official duties; on a worldwide, perpetual, irrevocable, royalty-free basis to BMJ Publishing Group Ltd ("BMJ") its licensees and where the relevant Journal is co-owned by BMJ to the co-owners of the Journal, to publish the Work in this journal and any other BMJ products and to exploit all rights, as set out in our licence.

The Submitting Author accepts and understands that any supply made under these terms is made by BMJ to the Submitting Author unless you are acting as an employee on behalf of your employer or a postgraduate student of an affiliated institution which is paying any applicable article publishing charge ("APC") for Open Access articles. Where the Submitting Author wishes to make the Work available on an Open Access basis (and intends to pay the relevant APC), the terms of reuse of such Open Access shall be governed by a Creative Commons licence – details of these licences and which Creative Commons licence will apply to this Work are set out in our licence referred to above.

Other than as permitted in any relevant BMJ Author's Self Archiving Policies, I confirm this Work has not been accepted for publication elsewhere, is not being considered for publication elsewhere and does not duplicate material already published. I confirm all authors consent to publication of this Work and authorise the granting of this licence.

For peer review only

Identification of biomarkers that predict response to deep brain stimulation over the subthalamic nucleus in obsessive-compulsive disorder

Shyam Sundar Arumugham[1*], Dwarakanath Srinivas[2], Janardhanan C Narayanaswamy[1], Jaisoorya TS[1], Himani Kashyap[3], Philippe Domenech[4,5], Stéphane Palfi[4,5], Luc Mallet[4,6,7], Ganesan Venkatasubramanian[1], YC Janardhan Reddy[1]

[1]Department of Psychiatry, National Institute of Mental health and Neurosciences, India
[2]Department of Neurosurgery, National Institute of Mental health and Neurosciences, India
[3]Department of Clinical Psychology, National Institute of Mental health and Neurosciences, India
[4]Univ Paris-Est Créteil, DMU CARE - Département Médical-Universitaire de Chirurgie et Anesthésie réanimation, DMU IMPACT, Département Médical-Universitaire de Psychiatrie et d'Addictologie, Hôpitaux Universitaires Henri Mondor - Albert Chenevier, Assistance Publique-Hôpitaux de Paris, Créteil, France
[5]U955 INSERM IMRB eq.15, Univ of Paris 12 UPEC, Faculté de médecine, Créteil, F-94010, France
[6]Institut du Cerveau, ICM, Inserm U 1127, CNRS UMR 7225, Sorbonne Université, F-75013, Paris, France
[7]Department of Mental Health and Psychiatry, Global Health Institute, University of Geneva, Geneva, Switzerland

*** Corresponding author**
Shyam Sundar Arumugham
DBT/Wellcome Trust India Alliance Intermediate Fellow
Additional Professor
Department of Psychiatry
National Institute of Mental Health and Neurosciences (NIMHANS)
Bengaluru, India 560029
Email- a.shyamsundar@gmail.com, shyamsundar.a@nimhans.ac.in
Phone- +91-80-26995353

Number of words: 3995
Number of tables: 2
Number of figures: 1

For peer review only

Trial registration: Clinical trials Registry – India (CTRI): CTRI/2020/03/024131

PROTOCOL VERSION

The current manuscript describes the version of the protocol approved by the institute ethics committee of NIMHANS dated 20th July 2019.

FUNDING

The study is fully funded by the DBT/Wellcome Trust India Alliance Intermediate fellowship granted to SSA. All the material/equipment including DBS electrodes would be purchased through funds from the fellowship.

ROLES & RESPONSIBILITIES

A. Contributors

SSA, the recipient of the Indian alliance fellowship, conceived and drafted the protocol YCJR advised on study design, clinical phenotyping, funding application and mentored the primary applicant; GVS mentored funding application and mentored the primary applicant, advised on study design, neuroimaging, electrophysiological recording; LM mentored the applicant, is the external sponsor for the fellowship, advised on study design, finalizing biomarkers and DBS targeting/procedure; PD advised on study design, DBS management, electrophysiological recording, biomarkers and DBS targeting/procedure; DS is a collaborator in the project advised on DBS procedures and study design; JCN is a collaborator who worked on the study design, advised on neuroimaging, electroencephalogram, neurocognitive assessment and study design; JTS advised on clinical phenotyping, study design and clinical recruitment, SP advised on DBS targeting and neurosurgical procedures, HK advised on neurocognitive assessment. All authors contributed to the refinement of the study protocol and approved the final version.

B. Sponsor

National Institute of Mental Health and Neuro Sciences (NIMHANS), Bengaluru, India
Contact: Director, NIMHANS, Hosur Road, Bengaluru, Karnataka, India 560029.
Email: dirstaff@nimhas.ac.in

C. Role of funding source

This funding source has no role in the design of this study and will not have any role during its execution, analyses, interpretation of the data, or decision to submit results

D. Committees

The Data Safety Monitoring Board (DSMB) consisting of independent specialists and a biostatistician will be constituted for overseeing the trial for its safety.

For peer review only

ABSTRACT

Introduction: Deep brain stimulation (DBS) over the anteromedial subthalamic nucleus (STN) has been proven to be effective in severe, chronic, and treatment-refractory obsessive-compulsive disorder (OCD). Biomarkers may aid in patient selection and optimization of this invasive treatment. In this trial, we intend to evaluate neurocognitive function related to STN and related biosignatures as potential biomarkers for STN-DBS in OCD.

Methods and analysis: Twenty-four subjects with treatment-refractory OCD will undergo open-label DBS over STN. Structural/functional imaging, electrophysiological recording, and neurocognitive assessment would be performed at baseline. The subjects would undergo a structured clinical assessment for 12 months post-surgery. A group of 24 healthy volunteers and 24 subjects with treatment-refractory OCD who receive treatment as usual (TAU) would be recruited for comparison of biomarkers and treatment response, respectively. Baseline biomarkers would be evaluated as predictors of clinical response. Neuroadaptive changes would be studied through a reassessment of neurocognitive functioning, imaging, and electrophysiological activity post-DBS.

Ethics and dissemination: The protocol has been approved by the NIMHANS Ethics Committee. The study findings will be disseminated through peer-reviewed scientific journals and scientific meetings.

Keywords: Obsessive-compulsive disorder, treatment-refractory, deep brain stimulation, subthalamic nucleus, biomarkers

STRENGTHS AND LIMITATIONS

- Multimodal evaluation of biomarkers through neuroimaging, scalp/invasive electrophysiological recording and neurocognitive evaluation
- Comparison group of treatment refractory OCD subjects with sufficient follow-up to help evaluating treatment response to DBS
- Limitations include open-label treatment and unblinded assessment.

For peer review only

INTRODUCTION

Obsessive-compulsive disorder (OCD) is a common neuropsychiatric condition with a lifetime prevalence of 2-2.5%.[1] It is among the top ten causes of neuropsychiatric disabilities worldwide.[2] The first-line treatment of OCD includes selective serotonin reuptake inhibitors (SSRIs) and/or cognitive-behaviour therapy (CBT).[3,4] However, around 20-30% of patients do not respond to standard treatment strategies.[5] Neurosurgical interventions are considered in patients with chronic, severe, and treatment-refractory OCD.[6] Ablative neurosurgical procedures, such as gamma ventral capsulotomy are helpful in around 45-65% of patients with treatment-refractory OCD.[5] Due to the potential irreversible and severe adverse effects associated with ablative procedures, there has been a surge of interest in deep brain stimulation (DBS) as an alternate treatment. DBS involves electrical stimulation of subcortical regions through surgically implanted microelectrodes. High frequency electrical stimulation through these electrodes modulate the activity of dysfunctional neuronal circuits.[7,8] Unlike ablative procedures, the stimulation in DBS can be modulated to optimise improvement and adverse effects.[9,10]

Cortico-striato-thalamo-cortical (CSTC) circuits passing through subcortical regions are implicated in the pathogenesis of OCD.[11,12] Thus, several subcortical structures including anterior limb of the internal capsule, ventral capsule/ventral striatum (VC/VS), nucleus accumbens (NAc), bed nucleus of stria terminalis, anteromedial subthalamic nucleus (amSTN), and inferior thalamic peduncle are targets for DBS in OCD. DBS over some of these targets has been found to significantly reduce OCD symptoms as compared to sham stimulation.[13] Based on evidence from a double-blinded randomised controlled trial,[14] a recent treatment guideline recommended DBS over bilateral STN for treatment refractory OCD.[15] Two recent randomised crossover trials found equivalent efficacy of DBS targeting amSTN as compared to VC/VS, caudate nucleus and NAc [16,17]. In the later study, most patients, who were masked/blinded to the target of stimulation, preferred the amSTN stimulation based on subjective improvement.[17] A recent systematic review also found similar efficacy for DBS over amSTN and striatal targets.[8] Interestingly, recent evidence suggests that the different targets have similar connections along fronto-subcortical circuits and thus may be targeting the same network.[18,19]

The adverse effects of DBS are frequently stimulation-related and generally mild and reversible.[7,20] DBS is an invasive procedure that requires long term close monitoring for optimization of stimulation as well as periodic changes in battery, which adds to the treatment cost. Despite these limitations, long-term follow-up studies have shown that DBS leads to improvement in symptoms as well as the quality of life.[21,22] However, DBS is helpful in only 60-75% of patients with treatment-refractory OCD.[20,22] There is a need to identify predictors of treatment response, which would assist in patient selection for this invasive and expensive treatment.

Despite decades of research, the exact mechanism of action of DBS is still not clearly understood.[23] Recent evidence suggests that DBS has both local and distant effects with resultant amelioration of pathological network activity.[11,23] The role of target nuclei such as amSTN in the pathophysiology of OCD provides useful clues to unravel the mechanism, which would help identify bio-predictors of response. Further, DBS provides a unique opportunity to study the role of STN in psychopathology with spatial and temporal precision.

Neurocircuitry of OCD

Although the aetiology of OCD is still unknown, converging evidence from neuroimaging studies implicates the dysfunctional CSTC circuits in the pathophysiology of OCD.[11,24] Particularly, the CSTC pathways involving the orbitofrontal cortex (OFC) and, to a lesser extent the dorsal anterior cingulate cortex (dACC) are dysfunctional in OCD.[25–28]

For peer review only

Functional neuroimaging studies show that these cortical regions are hyperactive at rest, which is accentuated during symptoms provocation.[29] Pretreatment OFC and caudate metabolism predict response to medications in OCD.[30,31] Similarly, DBS over various targets modulate the activity in these circuits by increasing their connectivity.[32,33] Thus, modulation of functional connectivity in the CSTC circuits is an important target for anti-obsessive treatments including DBS.

STN in the pathophysiology of OCD

STN is the key basal ganglia input structure of the "hyperdirect" pathways.[34] Computational models and imaging studies suggest that the "hyperdirect" pathway connecting inferior frontal gyrus (IFG) and STN is involved in global response inhibition, for example, during the stop-signal task (SST).[35,36] Functional magnetic resonance imaging (fMRI) studies have shown activation of STN, IFG and pre-supplementary motor area (pre-SMA) during SST performance.[36,37] STN also plays a crucial role in decision making and response selection in conflict situations, by setting a decision threshold that is contextually modulated.[38,39] Recent evidence suggests that neurons in ventromedial STN, which is preferentially connected to dACC and OFC, are especially involved in reactive stopping and switching.[40,41] Both these functions are impaired in patients with OCD.[42,43] Following DBS over STN, patients with OCD and Parkinson's disease show decreased reaction time and performance, suggesting more 'impulsivity', as defined in response inhibition paradigms.[44] STN DBS modulates uncertainty/conflict driven decision threshold adjustment and adapting to speed/accuracy trade-offs.[45] Thus, DBS over amSTN may decrease decisional threshold towards more optimal levels leading to a less cautious and more rapid goal-directed behaviour, which may be beneficial in patients with OCD.[46,47]

STN connectivity has been implicated in the pathophysiology of OCD. Resting-state functional connectivity of STN with cortical and striatal structures have been associated with cognitive and behavioural measures of compulsivity.[48] Tracing studies in non-human primates have found hyperdirect pathways connecting OFC, dACC and dorsolateral prefrontal cortex (DLPFC) projecting to the anteromedial STN [40]. A prospectively acquired imaging study found that effective STN DBS targets in OCD are located in this region i.e. in the anterior-inferior medial border, which has direct connections with the OFC, dACC and DLPFC.[16] Further, evidence suggests that STN DBS modulates cortico-striatal connectivity during SST.[47] STN DBS in OCD decreases metabolism in ACC and the therapeutic effects correlate with a decrease in OFC metabolism.[49] Thus, CSTC networks connecting various prefrontal regions with STN play a role in the neurocognitive functions underlying OCD, its behavioural manifestations and may mediate the therapeutic actions of DBS in OCD. The hyperdirect pathways have especially been implicated in this regard.[50]

Another line of evidence has utilised the unique opportunity provided by DBS to collect electrophysiological data directly from the nucleus. STN oscillatory activity and frontal cortico-STN coherence in β and θ frequency bands are associated with different phases of the SST.[51] The medial prefrontal cortex (mPFC)-STN θ-phase coherence increases during high-conflict trials in the Flanker task.[52] Response inhibition and conflict monitoring, assessed through SST and Flanker task respectively, are putative endophenotypes for OCD.[53,54] In OCD subjects, bursting and oscillatory activity have been localised to the associative/limbic STN, with predominant oscillatory activity in δ-band. Further, the severity of OCD is associated with low-frequency oscillatory activity and burst characteristics.[55] Low-frequency activity (θ-band) has been associated with symptom provocation and cognitive/emotional functioning in OCD subjects.[56] A recent study demonstrated that emotional images distinctively modulated STN θ-band activity in OCD subjects and emotion-related θ-band activity correlated with symptom severity.[57] Thus, electrophysiological

For peer review only

activity in STN, in particular low-frequency oscillatory activity, is a putative biomarker associated with neurocognitive functions underlying OCD and its clinical manifestations.

Overall, STN-cortical connectivity (especially with the frontal cortex), scalp electroencephalographic activity in the frontal cortex, bursting/oscillatory activity in the subthalamic nucleus, and STN-related neurocognitive functioning (response inhibition) are potential biomarkers for treatment response to DBS in OCD.

The current proposal plans to identify such biomarkers with the following objectives:

Primary objective:

i. To evaluate whether STN functional connectivity when performing response inhibition task predicts improvement in obsessive-compulsive symptoms after DBS over STN.

Secondary objectives:

i. To evaluate whether the improvement of obsessive-compulsive symptoms following DBS over STN is predicted by
   a. baseline STN resting-state functional connectivity with prefrontal cortex (PFC)
   b. STN functional connectivity with PFC regions during symptom provocation paradigm
   c. White matter structural connectivity (fractional anisotropy and other measures) of STN with cortical regions
   d. Local field potential time frequency amplitude in the STN at rest, during response inhibition task and symptom provocation
   e. Frontal EEG activity at rest, during response inhibition task and symptom provocation.
ii. To study the neuroadaptive changes following DBS by evaluating neurocognitive performance and EEG activity pre and 12-months post-DBS.
iii. To evaluate the effect of DBS on clinical symptomatology, disability, quality of life, and subjective well-being.

We hypothesise that baseline STN-prefrontal connectivity, low-frequency oscillatory activity over frontal lobes/STN, and impaired performance on response inhibition task would predict improvement in obsessive-compulsive symptoms after DBS over STN in OCD. The current report is prepared based on SPIRIT checklist.[58]

Experimental design:

We plan to conduct a longitudinal open-label follow-up study of 24 subjects undergoing DBS over STN for severe, chronic and treatment-refractory OCD to evaluate whether baseline biomarkers predict a decrease in the severity of OCD at follow-up. The DBS leads and stimulator would be implanted after baseline investigation for biomarkers. The initial 6 months of follow-up would be utilised for the optimization of stimulus parameters. This would be followed by an open-labelled naturalistic clinical follow-up of 6 months. The primary outcome would be a clinical improvement after 6 months of stable stimulation i.e. approximately 1 year after the implantation procedure. A comparison group consisting of 24 subjects with chronic, severe, and treatment-refractory OCD, who would receive treatment as usual (TAU), would also be followed-up for 1 year. We would also recruit a sample of 24 healthy volunteers for comparison of biomarkers at baseline (Figure-1).

Sample:

DBS Group (OCD-DBS)

Twenty-four subjects with severe, chronic, and treatment-refractory OCD would be recruited from the OCD clinic, in-patient and out-patient services of the National Institute of Mental

For peer review only

Health & Neuro Sciences (NIMHANS), Bangalore. The subjects would be screened with the following selection criteria:

Inclusion criteria

i. Age 18-60 years.
ii. Primary diagnosis of obsessive-compulsive disorder satisfying the DSM-5 criteria,[59] established using Mini International Neuropsychiatric Interview-7.0.2.[60]
iii. Duration of OCD ≥ 5 years.
iv. Yale-Brown Obsessive-Compulsive Scale (YBOCS)[61] score of ≥ 28 or ≥ 14 if the subject has either predominantly obsessions or compulsions alone.
v. Clinical Global Impressions – Severity (CGI-S)[62] scale of ≥ 5
vi. Disability of ≥ 40% as evaluated by the Indian Disability Evaluation and Assessment Scale (IDEAS).[63]
vii. Failure to obtain meaningful improvement in OCD despite adequate trial with standard treatment strategies which should include:
   a. At least 3 trials of serotonin reuptake inhibitors (SRIs), one of which should be clomipramine, which was either ineffective or poorly tolerated. An adequate trial includes the recommended dose of SRIs for ≥ 12 weeks duration each.[6]
   b. Augmentation of SRIs with at least 2 agents for ≥ 6 weeks, one of which should be either risperidone or aripiprazole.
   c. Trial of structured behaviour therapy/cognitive-behaviour therapy for at least 20 sessions or demonstrated an inability to tolerate the anxiety due to therapy.

Exclusion criteria

i. Diagnosis of bipolar disorder, a psychotic disorder of ≥ 3 months duration as assessed with MINI-7.0.2
ii. Current substance use disorder (except caffeine or nicotine use disorder) or Major depressive episode or current high suicidality, as assessed with MINI-7.0.2.
iii. Severe personality disorder as assessed with Structured Clinical Assessment of DSM-5-PD version.
iv. Clinically significant abnormality on magnetic resonance imaging (MRI) of the brain.
v. Pregnancy, contraindication for DBS/anaesthesia/preoperative MRI, inability to comply with surgical requirements.

All potential study participants satisfying the above criteria will be assessed for suitability for DBS by 2 psychiatrists and a neurosurgeon from the study team. An independent review committee with members (consisting of 2 psychiatrists, 1 neurologist, and 1 neurosurgeon) who are not part of the study team would ratify the decision for eligibility for surgery.

OCD control group (OCD-C)

We would recruit 24 OCD subjects fulfilling the above criteria from the same population, including those subjects who refuse consent for DBS. These subjects would receive treatment for OCD as recommended by the treating clinician.

Healthy volunteers (HC)

Twenty-four age and gender-matched healthy volunteers would be recruited from the community to compare the baseline differences in neuroimaging, neurocognitive performance, and electroencephalogram (EEG) with the study subjects. They would be screened for the presence of psychiatric disorder using MINI 7.0.2. Other exclusion criteria include Y-BOCS ≥ 12, family history of psychiatric disorder in first-degree relatives, clinically significant neurological illness, pregnancy, and contraindication for MRI.

For peer review only

Sample size estimation

For evaluating the above-mentioned primary objective, the optimal sample size was estimated using standard principles & methods.[48] Based on approximation & estimation from the previous data on fMRI-based predictors of treatment response for other interventions in OCD (n=12,15),[49,50] a sample size of at least 24 OCD patients will be required to detect a two-tailed significant difference of α = 0.05 with an estimated 90% power for an estimated correlation coefficient of 0.6 (Pearson's) between STN-PFC connectivity and change in Y-BOCS total score with add-on DBS.

Baseline assessment

Clinical assessment

The clinical status would be assessed with the MINI-7.0.2.[60] All OCD subjects would undergo baseline assessment with Yale-Brown Obsessive Compulsive Scale (Y-BOCS) which includes a symptom checklist to assess the nature of symptoms, severity scale to assess the severity of symptoms and insight/avoidance scale to assess these domains of symptoms. The severity of illness would also be assessed with the Clinical Global Impressions-Severity (CGI-S) scale. The severity of depressive and anxiety symptoms would be assessed with the Hamilton Depression Rating Scale (HAM-D),[64] and Hamilton Anxiety Rating Scale (HAM-A),[65] respectively. The extent of disability, quality of life and mental wellbeing would be assessed with the WHO Disability Assessment Schedule 2.0- 36 item version (WHODAS 2.0),[66] WHO quality of life instrument (WHOQOL-BREF),[67] and WHO-Five Well-Being Index (WHO-5).[68] The healthy volunteers would undergo screening with MINI-7.0.2 and Y-BOCS.

Neurocognitive assessment

All the subjects would undergo a detailed neurocognitive evaluation consisting of the modified NIMHANS neuropsychology battery,[69] Stop Signal Task (SST),[70] for assessing response inhibition, modified Flanker task,[53] for assessing response inhibition in a conflict situation/error monitoring, Beads task,[46] for assessing decisional impulsivity and temporal discounting task,[71–73] for assessing reward-related decision making. Besides, Frontotemporal Behavioural Scale,[74] the Iowa Scales for Personality Change,[75] and the UPPS Impulsive Behaviour Scale,[76] would be administered to assess for personality/behavioural dysfunction. Subjects from all three groups would undergo the neurocognitive/behavioural assessment (table-1) at baseline. Besides, OCD subjects would undergo a repeat evaluation at the end of 12 months.

Magnetic resonance imaging

Magnetic resonance imaging (MRI) will be acquired with a 3Tesla MRI scanner (Philips Ingenia) at NIMHANS, Bangalore. Both groups of OCD subjects and healthy volunteers would undergo a baseline structural MRI of the brain and diffusion tensor imaging (DTI) to evaluate the baseline structural connectivity of the STN. Subjects in the OCD-DBS group would undergo an additional baseline structural MRI with a stereotactic frame attached to guide the accurate targeting of electrodes. All subjects would undergo multi-echo (ME) resting-state functional MRI (fMRI) of the brain to study the functional connectivity of the STN. The subjects would also undergo a ME-fMRI while performing stop-signal task and under symptom provocation (only for the OCD-DBS and OCD-C subjects) employing symptom dimension-specific pictures. ME-fMRI during SST activates the STN and connected cortical regions.[37] Blood oxygenation level-dependent (BOLD) activity in this paradigm would be tested as primary predictors of treatment response to DBS. The symptom provocation paradigm activates cortico-striatal and limbic structures.[77] Activity in these regions has been found to

For peer review only

predict response to medications and CBT.[78,79] To the best of our knowledge, ME-fMRI has not been performed under symptom provocation. Hence, it is unclear whether this paradigm would activate STN. Due to the previous data showing activation of OCD specific circuits, activity under this paradigm will be tested as an additional predictor of treatment response.

Electroencephalography

All subjects would undergo an awake electroencephalogram (EEG) in a comfortable resting supine position from the frontocentral(FCz), central (Cz), and bilateral dorsolateral prefrontal (F3 & F4) montages as per the international 10-20 EEG system electrode placement. The OCD subjects and healthy volunteers would undergo EEG while performing the SST and flanker tasks. The OCD subjects would also undergo EEG undergo symptom provocation employing symptom dimension-specific pictures. Artefact free epochs of resting-state EEG epochs will be selected for power spectral analysis which involves examining the spectral power in various frequency bands (δ=1-3 Hz, θ = 4-7 Hz, α = 8-11 Hz, β =12-30 Hz, γ >30Hz) at each electrode. For examining the EEG under symptom provocation, power spectral analysis would be conducted on 2-second epochs selected from 1 second before stimuli to 1 second after the stimuli i.e. symptom dimension specific pictures.

Deep Brain stimulation

Following the baseline assessments, subjects in the OCD-DBS will undergo DBS over STN.

Surgical procedure

STN would be located under stereotactic MRI guidance. Quadripolar electrodes would be implanted at the boundary of limbic and associative territories of STN, bilaterally under local anaesthesia and antiseptic precautions. [14] The targets would be defined through intra-operative micro-recording along multiple trajectories.[80] Intraoperative macro-stimulation would be conducted to evaluate the acute effects of stimulation. The trajectory would be chosen based on acute adverse vis-a-vis beneficial effects. The position of the electrodes would be confirmed by postoperative MRI using a 3D atlas-MRI co-registration.[81] Electrophysiological activity from STN would be recorded perioperatively and postoperatively. The stimulator would be implanted subcutaneously 5-7 days following STN stimulation after electrophysiological recording.

Electrophysiological recording from STN

Perioperative single-unit recording would be recorded through microelectrodes used for STN localization. Spiking activity would be recorded during 2 minutes of rest and symptom provocation through stereotactically lowered microelectrode leads from various regions of the STN. Local field potentials (LFP) would be recorded through the DBS stimulating electrodes postoperatively. The electrodes would be externalised through the scalp with extension cables connected to an amplifier for LFP recording. Adequate precautions would be taken to prevent postoperative infection. The LFP recording would be done on the 3rd-5th postoperative days at rest, during SST, Flanker task and under symptom provocation. LFP recording would also be acquired during the performance in SST and Flanker task. Power spectral analysis would be performed to study oscillatory activity in various frequency bands (δ=1-3 Hz, θ = 4-7 Hz, α = 8-11 Hz, β =12-30 Hz, γ >30Hz).

Optimization of stimulation parameters

After the implantation of the stimulator, programming would be finalised over the subsequent 6 months to test various parameters including frequency, pulse-width, amplitude, and polarity

For peer review only

of the contacts stimulated. The electrophysiological parameters and active contacts would be chosen based on anatomical location, immediate effects, and adverse effects.

Follow-up intervention

Following the optimisation of parameters that may take up to 6 months post-implantation of stimulator and electrodes, the patient would be followed-up for another 6 months, considering the latency in onset of improvement with DBS in OCD. DBS would be provided as an unblinded add-on treatment. Although the subjects chosen are treatment-refractory, medication changes and CBT would be permitted during the follow-up period due to ethical concerns as well as improve the external validity of the study. CBT would also be permitted during follow-up as it may improve the outcome after DBS.[82]

Subjects in the OCD-C would undergo the best alternative treatment for treatment-refractory OCD based on the standard treatment practices of OCD clinic, NIMHANS, and evidence-based clinical practice guidelines. The participants would be unblinded to the group allotment. If they wish to undergo neurosurgical interventions including DBS for OCD during follow-up, they would be offered the same and would be removed from the control arm of the study.

Follow-up assessments

Clinical assessments

Subjects from both OCD-DBS and OCD-C groups would undergo structured clinical assessment during follow-up. Assessment during follow-up would include a clinical evaluation consisting of monthly assessment of Y-BOCS, CGI-S, HAM-D, and HAM-A (table-1). The subjects would do a self-assessment of their obsessive-compulsive symptoms using a smartphone-based app. WHODAS 2.0, WHOQOL-BREF and WHO-5 would be administered every 3 months. Y-BOCS scores at the end of 6 months of follow-up post-optimisation of stimulation parameters would be the primary outcome variable. The structured assessment would be performed through an in-person interview or video conferencing. Subjects in the OCD-C group would also be assessed with same baseline and follow-up clinical assessments, as specified above

Neurocognitive assessment, MRI and EEG

At the end of 6 months of stable stimulation (i.e. approximately 12 months post-implantation), both groups of OCD subjects would undergo a detailed neurocognitive assessment as well as EEG (using the same paradigms described above). The effect of DBS on neurocognitive and EEG markers would be evaluated and correlated with symptom change. Acquiring MRI scans with DBS electrodes in situ may carry the risk of image distortion, interference with DBS stimulator, and risk of tissue damage due to overheating of the electrodes.[83] However, recent reports suggest that MRI scans could be obtained safely, under appropriate precautions.[84–86] DBS manufacturers recommend precautions for acquiring MRI scans in this population, such as acquisition using 1.5 T MRI. Thus, post-surgical MRI scans (including T1-weighted structural images, fMRI and DTI) would be acquired under appropriate precautions, only for subjects who provide informed consent after explaining the risks and benefits associated with the procedure. Subjects in the OCD-C group would undergo follow-up MRI, EEG, and neuro assessments at the end of 12 months, employing the same paradigms and protocols as in the OCD-DBS group. As the subjects would not undergo surgery, the electrophysiological recording would not be obtained from the subthalamic nucleus. The assessment schedule for the 3 groups are is shown in table-2.

Plan for analysis

For peer review only

The OCD-C subjects would serve as a comparator for the intervention group to establish the efficacy of DBS. The baseline and follow-up biomarkers would be compared between the two groups employing the standard statistical techniques mentioned in the protocol. The healthy volunteer group would serve as a comparator to establish the baseline differences in MRI, EEG, and neurocognitive test performances with the OCD subjects.

The statistical analysis would be conducted using the Statistical Package for Social Sciences (SPSS) software (SPSS Inc., Chicago, IL). The fMRI processing and analysis would be conducted using the Statistical Parametric Mapping (SPM) software (http://www.fil.ion.ucl.ac.uk/spm/). Functional connectivity analysis would be conducted using SPM toolboxes and FSL software (https://fsl.fmrib.ox.ac.uk/fsl/fslwiki/). Resting-state fMRI would be analysed using SPM, FSL, DPABI toolbox (http://rfmri.org/dpabi) as per established processing pipelines. EEG analysis and electrophysiological analysis would be conducted using appropriate software. The demographic, clinical, neurocognitive, electrophysiological data will be examined for normality. Correlational followed by multiple regression analysis would be employed to assess whether the biomarkers predict clinical response (Y-BOCS score) at the end of 6 months of stable stimulation. Similar analyses would be conducted to test the other study objectives.

Ethical considerations

The study proposal and the informed consent forms have been approved by NIMHANS Ethics Committee [No.NIMHANS/EC(BEH.SC.DIV.) 19TH MEETING 2019 dated 16th July 2019]. The trial has been registered in the Clinical Trial Registry of India (CTRI/2020/03/024131). The subjects would be recruited after obtaining written informed consent explaining the risks and benefits of participating in the study. Subjects in the OCD-DBS group would be diligently educated about and monitored for adverse effects by trained mental health professionals. If subjects from the OCD-C group wish to undergo neurosurgical interventions for OCD during follow-up, they would be offered the same and would be removed from the control arm. Post-operative MRI would be conducted only in consenting subjects from the OCD (intervention) group after explaining the risks and benefits. The subjects in the intervention group would be protected by an insurance plan funded by the research proposal during the process of the study. A data safety monitoring board would be constituted to periodically review the progress of the study to assess safety issues and recommend continuation, modification, and termination of the trial, as required. All OCD subjects can continue to receive treatment from the OCD clinic, NIMHANS following the study proposal irrespective of whether they complete the study.

Dissemination

The data will be anonymised, curated, and stored in a computer hard disk, with appropriate backup. In keeping with the policies of the funding agency, the data and metadata would be shared with the wider research community through appropriate platforms. The study findings would be disseminated in peer-reviewed scientific publications and scientific conferences.

Expected outcome

Identification of biomarkers of treatment response to DBS over STN in OCD would pave way for personalised medicine by assisting patient selection for this invasive and expensive treatment. Further, this would help understand the mechanisms of action of treatment which would assist in future refinement/optimisation of treatment and open the scope for innovations, including closed-loop stimulation.

For peer review only

References

1 Torres AR, Lima MCP. [Epidemiology of obsessive-compulsive disorder: a review]. *Rev Bras Psiquiatr* 2005;**27**:237–42. doi:/S1516-44462005000400015

2 Collins PY, Patel V, Joestl SS, *et al.* Grand challenges in global mental health. *Nature* 2011;**475**:27–30. doi:10.1038/475027a

3 Arumugham SS, Reddy YCJ. Commonly asked questions in the treatment of obsessive-compulsive disorder. *Expert Rev Neurother* 2014;**14**:151–63. doi:10.1586/14737175.2014.874287

4 Fineberg NA, Hollander E, Pallanti S, *et al.* Clinical advances in obsessive-compulsive disorder: a position statement by the International College of Obsessive-Compulsive Spectrum Disorders. *Int Clin Psychopharmacol* 2020;**35**:173–93. doi:10.1097/YIC.0000000000000314

5 Bear RE, Fitzgerald P, Rosenfeld JV, *et al.* Neurosurgery for obsessive-compulsive disorder: contemporary approaches. *J Clin Neurosci* 2010;**17**:1–5. doi:10.1016/j.jocn.2009.02.043

6 Janardhan Reddy YC, Sundar AS, Narayanaswamy JC, *et al.* Clinical practice guidelines for Obsessive-Compulsive Disorder. *Indian J Psychiatry* 2017;**59**:S74–90. doi:10.4103/0019-5545.196976

7 Kohl S, Schönherr DM, Luigjes J, *et al.* Deep brain stimulation for treatment-refractory obsessive compulsive disorder: a systematic review. *BMC Psychiatry* 2014;**14**:214. doi:10.1186/s12888-014-0214-y

8 Vicheva P, Butler M, Shotbolt P. Deep brain stimulation for obsessive-compulsive disorder: A systematic review of randomised controlled trials. *Neurosci Biobehav Rev* 2020;**109**:129–38. doi:10.1016/j.neubiorev.2020.01.007

9 Kumar KK, Appelboom G, Lamsam L, *et al.* Comparative effectiveness of neuroablation and deep brain stimulation for treatment-resistant obsessive-compulsive disorder: a meta-analytic study. *J Neurol Neurosurg Psychiatry* 2019;**90**:469–73. doi:10.1136/jnnp-2018-319318

10 Balachander S, Arumugham SS, Srinivas D. Ablative neurosurgery and deep brain stimulation for obsessive-compulsive disorder. *Indian J Psychiatry* 2019;**61**:S77–84. doi:10.4103/psychiatry.IndianJPsychiatry_523_18

11 van den Heuvel OA, van Wingen G, Soriano-Mas C, *et al.* Brain circuitry of compulsivity. *Eur Neuropsychopharmacol* 2016;**26**:810–27. doi:10.1016/j.euroneuro.2015.12.005

12 Dougherty DD, Brennan BP, Stewart SE, *et al.* Neuroscientifically Informed Formulation and Treatment Planning for Patients With Obsessive-Compulsive Disorder: A Review. *JAMA Psychiatry* 2018;**75**:1081–7. doi:10.1001/jamapsychiatry.2018.0930

13 Martinho FP, Duarte GS, Couto FS do. Efficacy, Effect on Mood Symptoms, and Safety of Deep Brain Stimulation in Refractory Obsessive-Compulsive Disorder: A Systematic Review and Meta-Analysis. *The Journal of Clinical Psychiatry* 2020;**81**. doi:10.4088/JCP.19r12821

For peer review only

14 Mallet L, Polosan M, Jaafari N, *et al.* Subthalamic nucleus stimulation in severe obsessive-compulsive disorder. *N Engl J Med* 2008;**359**:2121–34. doi:10.1056/NEJMoa0708514

15 Hamani C, Pilitsis J, Rughani AI, *et al.* Deep brain stimulation for obsessive-compulsive disorder: systematic review and evidence-based guideline sponsored by the American Society for Stereotactic and Functional Neurosurgery and the Congress of Neurological Surgeons (CNS) and endorsed by the CNS and American Association of Neurological Surgeons. *Neurosurgery* 2014;**75**:327–33; quiz 333. doi:10.1227/NEU.0000000000000499

16 Tyagi H, Apergis-Schoute AM, Akram H, *et al.* A Randomized Trial Directly Comparing Ventral Capsule and Anteromedial Subthalamic Nucleus Stimulation in Obsessive-Compulsive Disorder: Clinical and Imaging Evidence for Dissociable Effects. *Biol Psychiatry* 2019;**85**:726–34. doi:10.1016/j.biopsych.2019.01.017

17 Welter M-L, Santos JFAD, Clair A-H, *et al.* Deep Brain Stimulation of the Subthalamic, Accumbens, or Caudate Nuclei for Patients With Severe Obsessive-Compulsive Disorder: A Randomized Crossover Controlled Study. *Biological Psychiatry* 2020;**0**. doi:10.1016/j.biopsych.2020.07.013

18 Li N, Baldermann JC, Kibleur A, *et al.* A unified connectomic target for deep brain stimulation in obsessive-compulsive disorder. *Nature Communications* 2020;**11**:3364. doi:10.1038/s41467-020-16734-3

19 Haber SN, Yendiki A, Jbabdi S. Four Deep Brain Stimulation Targets for Obsessive-Compulsive Disorder: Are They Different? *Biological Psychiatry* Published Online First: 25 July 2020. doi:10.1016/j.biopsych.2020.06.031

20 Alonso P, Cuadras D, Gabriëls L, *et al.* Deep Brain Stimulation for Obsessive-Compulsive Disorder: A Meta-Analysis of Treatment Outcome and Predictors of Response. *PLoS One* 2015;**10**. doi:10.1371/journal.pone.0133591

21 Ooms P, Mantione M, Figee M, *et al.* Deep brain stimulation for obsessive-compulsive disorders: long-term analysis of quality of life. *J Neurol Neurosurg Psychiatry* 2014;**85**:153–8. doi:10.1136/jnnp-2012-302550

22 Mallet L, Montcel STD, Clair A-H, *et al.* Long-term effects of subthalamic stimulation in Obsessive-Compulsive Disorder: Follow-up of a randomized controlled trial. *Brain Stimulation: Basic, Translational, and Clinical Research in Neuromodulation* 2019;**12**:1080–2. doi:10.1016/j.brs.2019.04.004

23 van Westen M, Rietveld E, Figee M, *et al.* Clinical Outcome and Mechanisms of Deep Brain Stimulation for Obsessive-Compulsive Disorder. *Curr Behav Neurosci Rep* 2015;**2**:41–8. doi:10.1007/s40473-015-0036-3

24 Saxena S, Rauch SL. Functional neuroimaging and the neuroanatomy of obsessive-compulsive disorder. *Psychiatr Clin North Am* 2000;**23**:563–86.

25 Burguière E, Monteiro P, Mallet L, *et al.* Striatal circuits, habits, and implications for obsessive-compulsive disorder. *Current Opinion in Neurobiology* 2015;**30**:59–65. doi:10.1016/j.conb.2014.08.008

26 Burguière E, Monteiro P, Feng G, *et al.* Optogenetic Stimulation of Lateral Orbitofronto-Striatal Pathway Suppresses Compulsive Behaviors. *Science* 2013;**340**:1243–6. doi:10.1126/science.1232380

For peer review only

27 Robbins TW, Vaghi MM, Banca P. Obsessive-Compulsive Disorder: Puzzles and Prospects. *Neuron* 2019;**102**:27–47. doi:10.1016/j.neuron.2019.01.046

28 Ahmari SE, Spellman T, Douglass NL, *et al.* Repeated Cortico-Striatal Stimulation Generates Persistent OCD-Like Behavior. *Science* 2013;**340**:1234–9. doi:10.1126/science.1234733

29 Rotge J-Y, Guehl D, Dilharreguy B, *et al.* Provocation of obsessive-compulsive symptoms: a quantitative voxel-based meta-analysis of functional neuroimaging studies. *J Psychiatry Neurosci* 2008;**33**:405–12.

30 Saxena S, Brody AL, Ho ML, *et al.* Differential Brain Metabolic Predictors of Response to Paroxetine in Obsessive-Compulsive Disorder Versus Major Depression. *Am J Psychiatry* 2003;**160**:522–32. doi:10.1176/appi.ajp.160.3.522

31 Saxena S, Brody AL, Maidment KM, *et al.* Localized orbitofrontal and subcortical metabolic changes and predictors of response to paroxetine treatment in obsessive-compulsive disorder. *Neuropsychopharmacology* 1999;**21**:683–93. doi:10.1016/S0893-133X(99)00082-2

32 Figee M, Luigjes J, Smolders R, *et al.* Deep brain stimulation restores frontostriatal network activity in obsessive-compulsive disorder. *Nat Neurosci* 2013;**16**:386–7. doi:10.1038/nn.3344

33 Millet B, Jaafari N, Polosan M, *et al.* Limbic versus cognitive target for deep brain stimulation in treatment-resistant depression: accumbens more promising than caudate. *Eur Neuropsychopharmacol* 2014;**24**:1229–39. doi:10.1016/j.euroneuro.2014.05.006

34 Mulders AEP, Plantinga BR, Schruers K, *et al.* Deep brain stimulation of the subthalamic nucleus in obsessive-compulsive disorder: Neuroanatomical and pathophysiological considerations. *Eur Neuropsychopharmacol* 2016;**26**:1909–19. doi:10.1016/j.euroneuro.2016.10.011

35 Wiecki TV, Frank MJ. A computational model of inhibitory control in frontal cortex and basal ganglia. *Psychol Rev* 2013;**120**:329–55. doi:10.1037/a0031542

36 Aron AR, Behrens TE, Smith S, *et al.* Triangulating a cognitive control network using diffusion-weighted magnetic resonance imaging (MRI) and functional MRI. *J Neurosci* 2007;**27**:3743–52. doi:10.1523/JNEUROSCI.0519-07.2007

37 Aron AR, Poldrack RA. Cortical and subcortical contributions to Stop signal response inhibition: role of the subthalamic nucleus. *J Neurosci* 2006;**26**:2424–33. doi:10.1523/JNEUROSCI.4682-05.2006

38 Frank MJ. Hold your horses: a dynamic computational role for the subthalamic nucleus in decision making. *Neural Netw* 2006;**19**:1120–36. doi:10.1016/j.neunet.2006.03.006

39 Herz DM, Little S, Pedrosa DJ, *et al.* Mechanisms Underlying Decision-Making as Revealed by Deep-Brain Stimulation in Patients with Parkinson's Disease. *Curr Biol* 2018;**28**:1169-1178.e6. doi:10.1016/j.cub.2018.02.057

40 Haynes WIA, Haber SN. The organization of prefrontal-subthalamic inputs in primates provides an anatomical substrate for both functional specificity and integration: implications for Basal Ganglia models and deep brain stimulation. *J Neurosci* 2013;**33**:4804–14. doi:10.1523/JNEUROSCI.4674-12.2013

For peer review only

41 Pasquereau B, Turner RS. A selective role for ventromedial subthalamic nucleus in inhibitory control. *eLife Sciences* 2017;**6**:e31627. doi:10.7554/eLife.31627

42 Chamberlain SR, Fineberg NA, Blackwell AD, *et al.* Motor Inhibition and Cognitive Flexibility in Obsessive-Compulsive Disorder and Trichotillomania. *AJP* 2006;**163**:1282–4. doi:10.1176/ajp.2006.163.7.1282

43 Gu B-M, Park J-Y, Kang D-H, *et al.* Neural correlates of cognitive inflexibility during task-switching in obsessive-compulsive disorder. *Brain* 2008;**131**:155–64. doi:10.1093/brain/awm277

44 Ballanger B, van Eimeren T, Moro E, *et al.* Stimulation of the subthalamic nucleus and impulsivity: release your horses. *Ann Neurol* 2009;**66**:817–24. doi:10.1002/ana.21795

45 Green N, Bogacz R, Huebl J, *et al.* Reduction of influence of task difficulty on perceptual decision making by STN deep brain stimulation. *Curr Biol* 2013;**23**:1681–4. doi:10.1016/j.cub.2013.07.001

46 Voon V, Droux F, Morris L, *et al.* Decisional impulsivity and the associative-limbic subthalamic nucleus in obsessive-compulsive disorder: stimulation and connectivity. *Brain* 2017;**140**:442–56. doi:10.1093/brain/aww309

47 Kibleur A, Gras-Combe G, Benis D, *et al.* Modulation of motor inhibition by subthalamic stimulation in obsessive-compulsive disorder. *Transl Psychiatry* 2016;**6**:e922. doi:10.1038/tp.2016.192

48 Morris LS, Baek K, Voon V. Distinct cortico-striatal connections with subthalamic nucleus underlie facets of compulsivity. *Cortex* 2017;**88**:143–50. doi:10.1016/j.cortex.2016.12.018

49 Le Jeune F, Vérin M, N'Diaye K, *et al.* Decrease of prefrontal metabolism after subthalamic stimulation in obsessive-compulsive disorder: a positron emission tomography study. *Biol Psychiatry* 2010;**68**:1016–22. doi:10.1016/j.biopsych.2010.06.033

50 Senova S, Clair A-H, Palfi S, *et al.* Deep Brain Stimulation for Refractory Obsessive-Compulsive Disorder: Towards an Individualized Approach. *Front Psychiatry* 2019;**10**. doi:10.3389/fpsyt.2019.00905

51 Alegre M, Lopez-Azcarate J, Obeso I, *et al.* The subthalamic nucleus is involved in successful inhibition in the stop-signal task: A local field potential study in Parkinson's disease. *Experimental Neurology* 2013;**239**:1–12. doi:10.1016/j.expneurol.2012.08.027

52 Zavala B, Tan H, Ashkan K, *et al.* Human subthalamic nucleus–medial frontal cortex theta phase coherence is involved in conflict and error related cortical monitoring. *NeuroImage* 2016;**137**:178–87. doi:10.1016/j.neuroimage.2016.05.031

53 Riesel A, Kathmann N, Endrass T. Overactive performance monitoring in obsessive-compulsive disorder is independent of symptom expression. *Eur Arch Psychiatry Clin Neurosci* 2014;**264**:707–17. doi:10.1007/s00406-014-0499-3

54 Chamberlain SR, Fineberg NA, Menzies LA, *et al.* Impaired Cognitive Flexibility and Motor Inhibition in Unaffected First-Degree Relatives of Patients With Obsessive-Compulsive Disorder. *AJP* 2007;**164**:335–8. doi:10.1176/ajp.2007.164.2.335

For peer review only

55 Welter M-L, Burbaud P, Fernandez-Vidal S, *et al.* Basal ganglia dysfunction in OCD: subthalamic neuronal activity correlates with symptoms severity and predicts high-frequency stimulation efficacy. *Translational Psychiatry* 2011;**1**:e5. doi:10.1038/tp.2011.5

56 Rappel P, Marmor O, Bick AS, *et al.* Subthalamic theta activity: a novel human subcortical biomarker for obsessive compulsive disorder. *Translational Psychiatry* 2018;**8**:1–11. doi:10.1038/s41398-018-0165-z

57 Buot A, Karachi C, Lau B, *et al.* Emotions Modulate Subthalamic Nucleus Activity: New Evidence in Obsessive-Compulsive Disorder and Parkinson's Disease Patients. *Biological Psychiatry: Cognitive Neuroscience and Neuroimaging* Published Online First: 11 August 2020. doi:10.1016/j.bpsc.2020.08.002

58 Chan A-W, Tetzlaff JM, Gøtzsche PC, *et al.* SPIRIT 2013 explanation and elaboration: guidance for protocols of clinical trials. *BMJ* 2013;**346**. doi:10.1136/bmj.e7586

59 American Psychiatric Association. *Diagnostic and Statistical Manual of Mental Disorders*. American Psychiatric Association 2013. http://dsm.psychiatryonline.org/doi/book/10.1176/appi.books.9780890425596 (accessed 24 Nov 2014).

60 Sheehan DV, Lecrubier Y, Sheehan KH, *et al.* The Mini-International Neuropsychiatric Interview (M.I.N.I.): the development and validation of a structured diagnostic psychiatric interview for DSM-IV and ICD-10. *J Clin Psychiatry* 1998;**59 Suppl 20**:22-33;quiz 34-57.

61 Goodman WK, Price LH, Rasmussen SA, *et al.* The Yale-Brown Obsessive Compulsive Scale. I. Development, use, and reliability. *Arch Gen Psychiatry* 1989;**46**:1006–11.

62 Guy W. ECDEU Assessment Manual for Psychopharmacology —Revised. Rockville, MD: : U.S. Department of Health, Education, and Welfare, Public Health Service, Alcohol, Drug Abuse, and Mental Health Administration, NIMH Psychopharmacology Research Branch, Division of Extramural Research Programs, 1976. 218–22.

63 The Rehabilitation Committee of the Indian Psychiatric Society. IDEAS (Indian Disability Evaluation and Assessment Scale) – A scale for measuring and quantifying disability in mental disorders. Gurgaon, India: : Indian Psychiatric Society

64 Hamilton M. A rating scale for depression. *J Neurol Neurosurg Psychiatr* 1960;**23**:56–62.

65 Hamilton M. The assessment of anxiety states by rating. *Br J Med Psychol* 1959;**32**:50–5.

66 WHO | WHO Disability Assessment Schedule 2.0 (WHODAS 2.0). WHO. http://www.who.int/classifications/icf/whodasii/en/ (accessed 16 May 2018).

67 Skevington SM, Lotfy M, O'Connell KA, *et al.* The World Health Organization's WHOQOL-BREF quality of life assessment: psychometric properties and results of the international field trial. A report from the WHOQOL group. *Qual Life Res* 2004;**13**:299–310. doi:10.1023/B:QURE.0000018486.91360.00

68 Bech P, Olsen LR, Kjoller M, *et al.* Measuring well-being rather than the absence of distress symptoms: a comparison of the SF-36 Mental Health subscale and the WHO-Five Well-Being Scale. *Int J Methods Psychiatr Res* 2003;**12**:85–91.

For peer review only

69 Rao S, Subbakrishna DK, Gopukumar K. *NIMHANS Neuropsychological Battery*. Bangalore, India: : National Institute of Mental Health and Neurosciences 2004.

70 Logan GD, Cowan WB, Davis KA. On the ability to inhibit simple and choice reaction time responses: a model and a method. *J Exp Psychol Hum Percept Perform* 1984;**10**:276–91. doi:10.1037//0096-1523.10.2.276

71 Kable JW, Glimcher PW. The neural correlates of subjective value during intertemporal choice. *Nature Neuroscience* 2007;**10**:1625–33. doi:10.1038/nn2007

72 Figner B, Knoch D, Johnson EJ, *et al.* Lateral prefrontal cortex and self-control in intertemporal choice. *Nature Neuroscience* 2010;**13**:538–9. doi:10.1038/nn.2516

73 Steinglass JE, Lempert KM, Choo T-H, *et al.* Temporal discounting across three psychiatric disorders: Anorexia nervosa, obsessive compulsive disorder, and social anxiety disorder. *Depress Anxiety* 2017;**34**:463–70. doi:10.1002/da.22586

74 Lebert F, Pasquier F, Souliez L, *et al.* Frontotemporal behavioral scale. *Alzheimer Dis Assoc Disord* 1998;**12**:335–9.

75 Barrash J, Anderson S. *The Iowa Scales for Personality Change*. Iowa City: : University of Iowa, Department of Neurology 1997.

76 Whiteside SP, Lynam DR. The Five Factor Model and impulsivity: using a structural model of personality to understand impulsivity. *Personality and Individual Differences* 2001;**30**:669–89. doi:10.1016/S0191-8869(00)00064-7

77 Simon D, Adler N, Kaufmann C, *et al.* Amygdala hyperactivation during symptom provocation in obsessive-compulsive disorder and its modulation by distraction. *Neuroimage Clin* 2014;**4**:549–57. doi:10.1016/j.nicl.2014.03.011

78 Sanematsu H, Nakao T, Yoshiura T, *et al.* Predictors of treatment response to fluvoxamine in obsessive-compulsive disorder: an fMRI study. *J Psychiatr Res* 2010;**44**:193–200. doi:10.1016/j.jpsychires.2009.08.007

79 Olatunji BO, Davis ML, Powers MB, *et al.* Cognitive-behavioral therapy for obsessive-compulsive disorder: a meta-analysis of treatment outcome and moderators. *J Psychiatr Res* 2013;**47**:33–41. doi:10.1016/j.jpsychires.2012.08.020

80 Hutchison WD, Allan RJ, Opitz H, *et al.* Neurophysiological identification of the subthalamic nucleus in surgery for Parkinson's disease. *Ann Neurol* 1998;**44**:622–8. doi:10.1002/ana.410440407

81 Yelnik J, Damier P, Demeret S, *et al.* Localization of stimulating electrodes in patients with Parkinson disease by using a three-dimensional atlas-magnetic resonance imaging coregistration method. *J Neurosurg* 2003;**99**:89–99. doi:10.3171/jns.2003.99.1.0089

82 Mantione M, Nieman DH, Figee M, *et al.* Cognitive-behavioural therapy augments the effects of deep brain stimulation in obsessive-compulsive disorder. *Psychol Med* 2014;**44**:3515–22. doi:10.1017/S0033291714000956

83 Carmichael DW, Pinto S, Limousin-Dowsey P, *et al.* Functional MRI with active, fully implanted, deep brain stimulation systems: safety and experimental confounds. *Neuroimage* 2007;**37**:508–17. doi:10.1016/j.neuroimage.2007.04.058

For peer review only

84 Bronstein JM, Tagliati M, Alterman RL, *et al.* Deep brain stimulation for Parkinson disease: an expert consensus and review of key issues. *Arch Neurol* 2011;**68**:165. doi:10.1001/archneurol.2010.260

85 Zrinzo L, Yoshida F, Hariz MI, *et al.* Clinical Safety of Brain Magnetic Resonance Imaging with Implanted Deep Brain Stimulation Hardware: Large Case Series and Review of the Literature. *World Neurosurgery* 2011;**76**:164–72. doi:10.1016/j.wneu.2011.02.029

86 Saleh C, Dooms G, Berthold C, *et al.* Post-operative imaging in deep brain stimulation: A controversial issue. *Neuroradiol J* 2016;**29**:244–9. doi:10.1177/1971400916639960

For peer review only

Authors contributions:

SSA, the recipient of the Indian alliance fellowship, conceived and drafted the protocol YCJR advised on study design, clinical phenotyping, funding application and mentored the primary applicant; GVS mentored funding application and mentored the primary applicant, advised on study design, neuroimaging, electrophysiological recording; LM mentored the applicant, is the external sponsor for the fellowship, advised on study design, finalizing biomarkers and DBS targeting/procedure; DS is a collaborator in the project advised on DBS procedures and study design; JCN is a collaborator who worked on the study design, advised on neuroimaging, electroencephalogram, neurocognitive assessment and study design; JTS advised on clinical phenotyping, study design and clinical recruitment, HK advised on neurocognitive assessment. All authors contributed to the refinement of the study protocol and approved the final version.

Funding statement

The study is fully supported by DBT/Wellcome Trust India Alliance Intermediate fellowship granted to SSA (grant number IA/CPHI/18/1/503931). All material/equipment including DBS electrodes would be funded through the fellowship.

Acknowledgement

GV acknowledges the support of the DBT Wellcome Trust India Alliance Clinical/Public Health Research Centre Grant (IA/CRC/19/1/610005).

Competing interests statement: None declared

Patient and public involvement: Patients and public were not involved in the design of this protocol.

Patient consent for publication: Not required

Word count: 3995

Table-1 Neurocognitive & personality assessment

Neurocognitive assessment

1. Modified NIMHANS Neuropsychology battery[69]
    - Finger Tapping
    - Bender Gestalt Test
    - Digit Symbol Substitution Test
    - Colour Trails 1 And 2
    - Block Design test
    - Controlled Oral Word Association Test
    - Animal Names Test
    - Digit Span
    - Spatial Span
    - Tower of London
    - Wisconsin Card Sorting Task
    - Stroop Test
    - Auditory Verbal Learning Test
    - Rey's Complex Figure Test
2. Stop Signal Task[70]
3. Modified Flanker task[53]
4. Beads task[46]
5. Temporal discounting task[71–73]

Personality/Behavioural assessment

1. Frontotemporal Behavioural Scale[74]
2. The Iowa Scales for Personality Change[75]
3. UPPS Impulsive Behaviour Scale[76]

For peer review only

Table-2 Assessment schedule

	**OCD Intervention group**	**OCD control group**	**Healthy volunteers**
MINI 7.0.2	Baseline	Baseline	Baseline
Y-BOCS	Baseline + monthly	Baseline + monthly	Baseline
CGI-S	Baseline + monthly	Baseline + monthly	Baseline
HAM-A	Baseline + monthly	Baseline + monthly	Baseline
HAM-D	Baseline + monthly	Baseline + monthly	Baseline
WHOQOL-BREF	Baseline + 3-monthly	Baseline + 3-monthly	Baseline
WHODAS 2.0	Baseline + 3-monthly	Baseline + 3-monthly	Baseline
WHO-5	Baseline + 3-monthly	Baseline + 3-monthly	Baseline
Neurocognitive testing	Baseline + end of 12 months	Baseline + end of 12 months	Baseline
MRI	Baseline + end of 12 months*	Baseline + end of 12 months	Baseline
EEG	Baseline + end of 12 months	Baseline + end of 12 months	Baseline
Subthalamic nucleus recording	Baseline	**x**	**x**

MINI 7.0.2 Mini International Neuropsychiatric interview 7.0.2,[60] Y-BOCS Yale Brown Obsessive Compulsive Scale,[61] CGI-S Clinical Global Impressions-Severity,[62] HAM-A Hamilton Anxiety Rating Scale,[65] HAM-D Hamilton Depression Rating Scale,[64] WHOQOL-BREF WHO quality of life instrument,[67] WHODAS 2.0 WHO Disability Assessment Schedule,[66] WHO-5 WHO-Five Well-Being Index,[68] MRI – Magnetic Resonance Imaging, EEG - Electroencephalography

Figure 1 Schematic representation of study protocol

All subjects would undergo baseline clinical, neurocognitive, MRI (magnetic resonance imaging) and EEG (electroencephalography) at baseline. For the subjects in the OCD-DBS group i.e. obsessive-compulsive disorder (OCD) subjects who receive DBS, stimulation parameters would be optimised over the next 6 months; followed by for another 6 months of clinical follow-up. Subjects in the OCD-C group i.e. OCD control subjects who receive treatment as usual, would undergo clinical follow-up for 12 months. Both the OCD groups would undergo neurocognitive assessment, MRI and EEG at the end of 12 months. Subjects in HC group (healthy controls) would not undergo any further assessment following baseline.

For peer review only - http://bmjopen.bmj.com/site/about/guidelines.xhtml

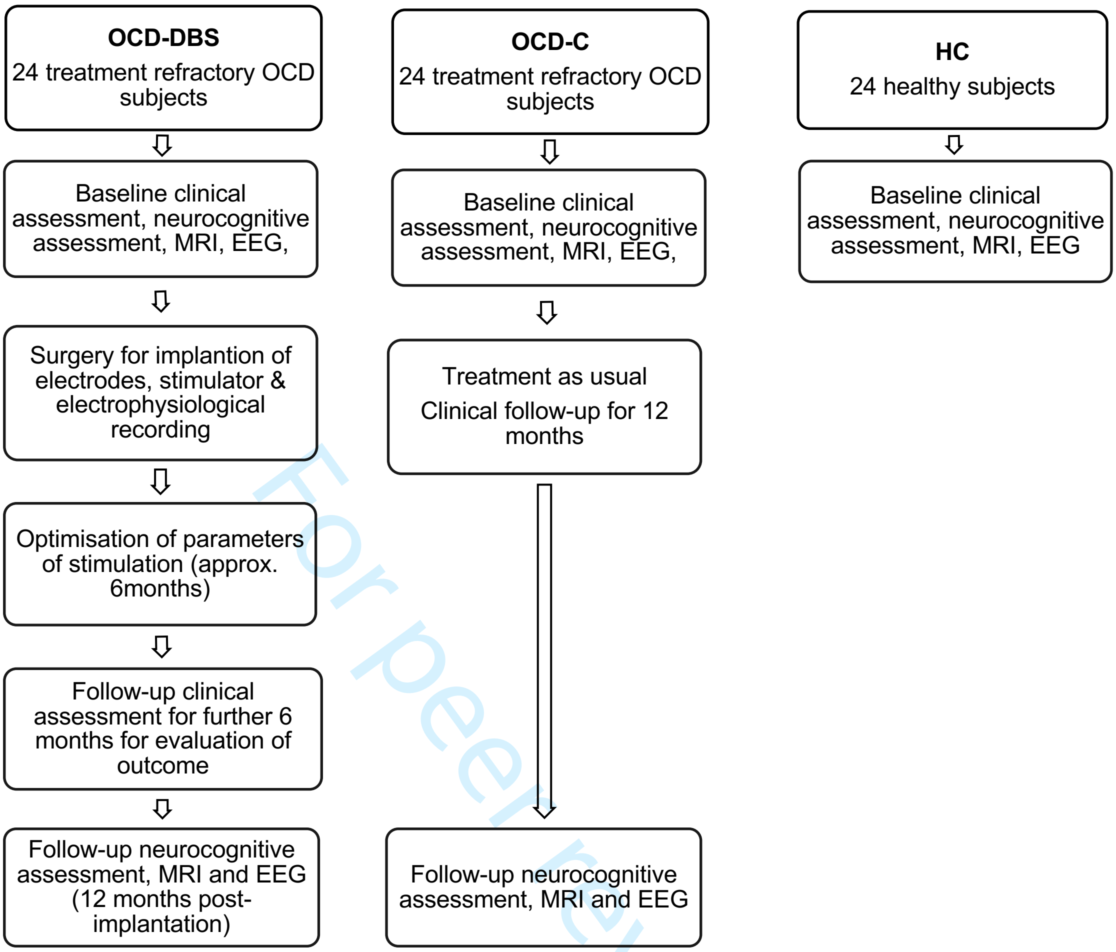

For peer review only

Reporting checklist for protocol of a clinical trial.

Based on the SPIRIT guidelines.

Instructions to authors

Complete this checklist by entering the page numbers from your manuscript where readers will find each of the items listed below.

Your article may not currently address all the items on the checklist. Please modify your text to include the missing information. If you are certain that an item does not apply, please write "n/a" and provide a short explanation.

Upload your completed checklist as an extra file when you submit to a journal.

In your methods section, say that you used the SPIRITreporting guidelines, and cite them as:

Chan A-W, Tetzlaff JM, Gøtzsche PC, Altman DG, Mann H, Berlin J, Dickersin K, Hróbjartsson A, Schulz KF, Parulekar WR, Krleža-Jerić K, Laupacis A, Moher D. SPIRIT 2013 Explanation and Elaboration: Guidance for protocols of clinical trials. BMJ. 2013;346:e7586

		Reporting Item	Page Number
**Administrative information**			
Title	#1	Descriptive title identifying the study design, population, interventions, and, if applicable, trial acronym	1
Trial registration	#2a	Trial identifier and registry name. If not yet registered, name of intended registry	2
Trial registration: data set	#2b	All items from the World Health Organization Trial Registration Data Set	Supp. material-2
Protocol version	#3	Date and version identifier	2
Funding	#4	Sources and types of financial, material, and other support	2
Roles and responsibilities: contributorship	#5a	Names, affiliations, and roles of protocol contributors	2

For peer review only

Roles and responsibilities: sponsor contact information	#5b	Name and contact information for the trial sponsor	4
Roles and responsibilities: sponsor and funder	#5c	Role of study sponsor and funders, if any, in study design; collection, management, analysis, and interpretation of data; writing of the report; and the decision to submit the report for publication, including whether they will have ultimate authority over any of these activities	2
Roles and responsibilities: committees	#5d	Composition, roles, and responsibilities of the coordinating centre, steering committee, endpoint adjudication committee, data management team, and other individuals or groups overseeing the trial, if applicable (see Item 21a for data monitoring committee)	2
**Introduction**			
Background and rationale	#6a	Description of research question and justification for undertaking the trial, including summary of relevant studies (published and unpublished) examining benefits and harms for each intervention	4-5
Background and rationale: choice of comparators	#6b	Explanation for choice of comparators	NA
Objectives	#7	Specific objectives or hypotheses	6
Trial design	#8	Description of trial design including type of trial (eg, parallel group, crossover, factorial, single group), allocation ratio, and framework (eg, superiority, equivalence, non-inferiority, exploratory)	6
**Methods: Participants, interventions, and outcomes**			
Study setting	#9	Description of study settings (eg, community clinic, academic hospital) and list of countries where data will be collected. Reference to where list of study sites can be obtained	6-7
Eligibility criteria	#10	Inclusion and exclusion criteria for participants. If applicable, eligibility criteria for study centres and individuals who will	7

For peer review only

		perform the interventions (eg, surgeons, psychotherapists)	
Interventions: description	#11a	Interventions for each group with sufficient detail to allow replication, including how and when they will be administered	9-10
Interventions: modifications	#11b	Criteria for discontinuing or modifying allocated interventions for a given trial participant (eg, drug dose change in response to harms, participant request, or improving / worsening disease)	9-10
Interventions: adherance	#11c	Strategies to improve adherence to intervention protocols, and any procedures for monitoring adherence (eg, drug tablet return; laboratory tests)	9-10
Interventions: concomitant care	#11d	Relevant concomitant care and interventions that are permitted or prohibited during the trial	9-10
Outcomes	#12	Primary, secondary, and other outcomes, including the specific measurement variable (eg, systolic blood pressure), analysis metric (eg, change from baseline, final value, time to event), method of aggregation (eg, median, proportion), and time point for each outcome. Explanation of the clinical relevance of chosen efficacy and harm outcomes is strongly recommended	10
Participant timeline	#13	Time schedule of enrolment, interventions (including any run-ins and washouts), assessments, and visits for participants. A schematic diagram is highly recommended (see Figure)	10 & figure-1
Sample size	#14	Estimated number of participants needed to achieve study objectives and how it was determined, including clinical and statistical assumptions supporting any sample size calculations	8
Recruitment	#15	Strategies for achieving adequate participant enrolment to reach target sample size	6-7
**Methods: Assignment of interventions (for controlled trials)**			
Allocation: sequence generation	#16a	Method of generating the allocation sequence (eg, computer-generated random numbers), and list of any factors for stratification. To reduce predictability of a random sequence, details of any planned restriction (eg, blocking) should be provided in a separate document that is unavailable to those who enrol participants or assign interventions	NA

For peer review only

Allocation concealment mechanism	#16b	Mechanism of implementing the allocation sequence (eg, central telephone; sequentially numbered, opaque, sealed envelopes), describing any steps to conceal the sequence until interventions are assigned	NA
Allocation: implementation	#16c	Who will generate the allocation sequence, who will enrol participants, and who will assign participants to interventions	NA
Blinding (masking)	#17a	Who will be blinded after assignment to interventions (eg, trial participants, care providers, outcome assessors, data analysts), and how	NA
Blinding (masking): emergency unblinding	#17b	If blinded, circumstances under which unblinding is permissible, and procedure for revealing a participant's allocated intervention during the trial	NA
**Methods: Data collection, management, and analysis**			
Data collection plan	#18a	Plans for assessment and collection of outcome, baseline, and other trial data, including any related processes to promote data quality (eg, duplicate measurements, training of assessors) and a description of study instruments (eg, questionnaires, laboratory tests) along with their reliability and validity, if known. Reference to where data collection forms can be found, if not in the protocol	10,19,20
Data collection plan: retention	#18b	Plans to promote participant retention and complete follow-up, including list of any outcome data to be collected for participants who discontinue or deviate from intervention protocols	10
Data management	#19	Plans for data entry, coding, security, and storage, including any related processes to promote data quality (eg, double data entry; range checks for data values). Reference to where details of data management procedures can be found, if not in the protocol	11
Statistics: outcomes	#20a	Statistical methods for analysing primary and secondary outcomes. Reference to where other details of the statistical analysis plan can be found, if not in the protocol	11
Statistics: additional analyses	#20b	Methods for any additional analyses (eg, subgroup and adjusted analyses)	11

For peer review only

Statistics: analysis population and missing data	#20c	Definition of analysis population relating to protocol non-adherence (eg, as randomised analysis), and any statistical methods to handle missing data (eg, multiple imputation)	11
**Methods: Monitoring**			
Data monitoring: formal committee	#21a	Composition of data monitoring committee (DMC); summary of its role and reporting structure; statement of whether it is independent from the sponsor and competing interests; and reference to where further details about its charter can be found, if not in the protocol. Alternatively, an explanation of why a DMC is not needed	2
Data monitoring: interim analysis	#21b	Description of any interim analyses and stopping guidelines, including who will have access to these interim results and make the final decision to terminate the trial	NA
Harms	#22	Plans for collecting, assessing, reporting, and managing solicited and spontaneously reported adverse events and other unintended effects of trial interventions or trial conduct	11
Auditing	#23	Frequency and procedures for auditing trial conduct, if any, and whether the process will be independent from investigators and the sponsor	NA
**Ethics and dissemination**			
Research ethics approval	#24	Plans for seeking research ethics committee / institutional review board (REC / IRB) approval	11
Protocol amendments	#25	Plans for communicating important protocol modifications (eg, changes to eligibility criteria, outcomes, analyses) to relevant parties (eg, investigators, REC / IRBs, trial participants, trial registries, journals, regulators)	NA
Consent or assent	#26a	Who will obtain informed consent or assent from potential trial participants or authorised surrogates, and how (see Item 32)	11
Consent or assent: ancillary studies	#26b	Additional consent provisions for collection and use of participant data and biological specimens in ancillary studies, if applicable	NA
Confidentiality	#27	How personal information about potential and enrolled participants will be collected, shared, and maintained in order to protect	11

For peer review only

		confidentiality before, during, and after the trial	
Declaration of interests	#28	Financial and other competing interests for principal investigators for the overall trial and each study site	18
Data access	#29	Statement of who will have access to the final trial dataset, and disclosure of contractual agreements that limit such access for investigators	11
Ancillary and post trial care	#30	Provisions, if any, for ancillary and post-trial care, and for compensation to those who suffer harm from trial participation	11
Dissemination policy: trial results	#31a	Plans for investigators and sponsor to communicate trial results to participants, healthcare professionals, the public, and other relevant groups (eg, via publication, reporting in results databases, or other data sharing arrangements), including any publication restrictions	11
Dissemination policy: authorship	#31b	Authorship eligibility guidelines and any intended use of professional writers	NA
Dissemination policy: reproducible research	#31c	Plans, if any, for granting public access to the full protocol, participant-level dataset, and statistical code	11
**Appendices**			
Informed consent materials	#32	Model consent form and other related documentation given to participants and authorised surrogates	Supp. material-1
Biological specimens	#33	Plans for collection, laboratory evaluation, and storage of biological specimens for genetic or molecular analysis in the current trial and for future use in ancillary studies, if applicable	NA

The SPIRIT checklist is distributed under the terms of the Creative Commons Attribution License CC-BY-ND 3.0. This checklist was completed on 27. November 2020 using https://www.goodreports.org/, a tool made by the EQUATOR Network in collaboration with Penelope.ai

For peer review only

Identification of biomarkers that predict response to subthalamic nucleus deep brain stimulation in obsessive-compulsive disorder

Journal:	*BMJ Open*
Manuscript ID	bmjopen-2020-047492.R1
Article Type:	Protocol
Date Submitted by the Author:	18-May-2021

Complete List of Authors:	Arumugham, Shyam Sundar; National Institute of Mental Health and Neuro Sciences, Psychiatry<br>Srinivas, Dwarakanath; National Institute of Mental Health and Neuro Sciences, Neurosurgery<br>Narayanaswamy, Janardhanan ; National Institute of Mental Health and Neuro Sciences, Psychiatry<br>Jaisoorya, TS; National Institute of Mental Health and Neuro Sciences, Psychiatry<br>Kashyap, Himani; National Institute of Mental Health and Neuro Sciences, Clinical Psychology<br>Domenech, Philippe; Hôpitaux Universitaires Henri Mondor, Univ Paris-Est Créteil, DMU CARE - Département Médical-Universitaire de Chirurgie et Anesthésie réanimation, DMU IMPACT, Département Médical-Universitaire de Psychiatrie et d'Addictologie; INSERM U955, Univ of Paris 12 UPEC, Faculté de médecine<br>Palfi, Stéphane; Hôpitaux Universitaires Henri Mondor, Univ Paris-Est Créteil, DMU CARE - Département Médical-Universitaire de Chirurgie et Anesthésie réanimation, DMU IMPACT, Département Médical-Universitaire de Psychiatrie et d'Addictologie; INSERM U955, Univ of Paris 12 UPEC, Faculté de médecine<br>Mallet, Luc; Sorbonne Université, Institut du Cerveau, ICM, Inserm U 1127, CNRS UMR 7225; University of Geneva, Department of Mental Health and Psychiatry<br>Venkatasubramanian, Ganesan; National Institute of Mental Health and Neuro Sciences, Psychiatry<br>Reddy, YC Janardhan; National Institute of Mental Health and Neuro Sciences, Psychiatry

**Primary Subject Heading**:	Mental health
Secondary Subject Heading:	Surgery
Keywords:	PSYCHIATRY, NEUROSURGERY, Anxiety disorders < PSYCHIATRY

SCHOLARONE™
Manuscripts

For peer review only - http://bmjopen.bmj.com/site/about/guidelines.xhtml

I, the Submitting Author has the right to grant and does grant on behalf of all authors of the Work (as defined in the below author licence), an exclusive licence and/or a non-exclusive licence for contributions from authors who are: i) UK Crown employees; ii) where BMJ has agreed a CC-BY licence shall apply, and/or iii) in accordance with the terms applicable for US Federal Government officers or employees acting as part of their official duties; on a worldwide, perpetual, irrevocable, royalty-free basis to BMJ Publishing Group Ltd ("BMJ") its licensees and where the relevant Journal is co-owned by BMJ to the co-owners of the Journal, to publish the Work in this journal and any other BMJ products and to exploit all rights, as set out in our licence.

The Submitting Author accepts and understands that any supply made under these terms is made by BMJ to the Submitting Author unless you are acting as an employee on behalf of your employer or a postgraduate student of an affiliated institution which is paying any applicable article publishing charge ("APC") for Open Access articles. Where the Submitting Author wishes to make the Work available on an Open Access basis (and intends to pay the relevant APC), the terms of reuse of such Open Access shall be governed by a Creative Commons licence – details of these licences and which Creative Commons licence will apply to this Work are set out in our licence referred to above.

Other than as permitted in any relevant BMJ Author's Self Archiving Policies, I confirm this Work has not been accepted for publication elsewhere, is not being considered for publication elsewhere and does not duplicate material already published. I confirm all authors consent to publication of this Work and authorise the granting of this licence.

For peer review only

Identification of biomarkers that predict response to subthalamic nucleus deep brain stimulation in obsessive-compulsive disorder

Shyam Sundar Arumugham[1*], Dwarakanath Srinivas[2], Janardhanan C Narayanaswamy[1], Jaisoorya TS[1], Himani Kashyap[3], Philippe Domenech[4,5], Stéphane Palfi[4,5], Luc Mallet[4,6,7], Ganesan Venkatasubramanian[1], YC Janardhan Reddy[1]

[1]Department of Psychiatry, National Institute of Mental health and Neurosciences, India
[2]Department of Neurosurgery, National Institute of Mental health and Neurosciences, India
[3]Department of Clinical Psychology, National Institute of Mental health and Neurosciences, India
[4]Univ Paris-Est Créteil, DMU CARE - Département Médical-Universitaire de Chirurgie et Anesthésie réanimation, DMU IMPACT, Département Médical-Universitaire de Psychiatrie et d'Addictologie, Hôpitaux Universitaires Henri Mondor - Albert Chenevier, Assistance Publique-Hôpitaux de Paris, Créteil, France
[5]U955 INSERM IMRB eq.15, Univ of Paris 12 UPEC, Faculté de médecine, Créteil, F-94010, France
[6]Institut du Cerveau, ICM, Inserm U 1127, CNRS UMR 7225, Sorbonne Université, F-75013, Paris, France
[7]Department of Mental Health and Psychiatry, Global Health Institute, University of Geneva, Geneva, Switzerland

*** Corresponding author**
Shyam Sundar Arumugham
DBT/Wellcome Trust India Alliance Intermediate Fellow
Additional Professor
Department of Psychiatry
National Institute of Mental Health and Neurosciences (NIMHANS)
Bengaluru, India 560029
Email- a.shyamsundar@gmail.com, shyamsundar.a@nimhans.ac.in
Phone- +91-80-26995353

Number of words: 4265
Number of tables: 2
Number of figures: 1

For peer review only

Trial registration: Clinical trials Registry – India (CTRI): CTRI/2020/03/024131

PROTOCOL VERSION

The current manuscript describes the version of the protocol approved by the institute ethics committee of NIMHANS dated 20th July 2019.

FUNDING

The study is fully funded by the DBT/Wellcome Trust India Alliance Intermediate fellowship granted to SSA. All the material/equipment including DBS electrodes would be purchased through funds from the fellowship.

ROLES & RESPONSIBILITIES

A. Contributors

SSA, the recipient of the Indian alliance fellowship, conceived and drafted the protocol YCJR advised on study design, clinical phenotyping, funding application and mentored the primary applicant; GVS mentored funding application and mentored the primary applicant, advised on study design, neuroimaging, electrophysiological recording; LM mentored the applicant, is the external sponsor for the fellowship, advised on study design, finalizing biomarkers and DBS targeting/procedure; PD advised on study design, DBS management, electrophysiological recording, biomarkers and DBS targeting/procedure; DS is a collaborator in the project advised on DBS procedures and study design; JCN is a collaborator who worked on the study design, advised on neuroimaging, electroencephalogram, neurocognitive assessment and study design; JTS advised on clinical phenotyping, study design and clinical recruitment, SP advised on DBS targeting and neurosurgical procedures, HK advised on neurocognitive assessment. All authors contributed to the refinement of the study protocol and approved the final version.

B. Sponsor

National Institute of Mental Health and Neuro Sciences (NIMHANS), Bengaluru, India
Contact: Director, NIMHANS, Hosur Road, Bengaluru, Karnataka, India 560029.
Email: dirstaff@nimhas.ac.in

C. Role of funding source

This funding source has no role in the design of this study and will not have any role during its execution, analyses, interpretation of the data, or decision to submit results

D. Committees

The Data Safety Monitoring Board (DSMB) consisting of independent specialists and a biostatistician will be constituted for overseeing the trial for its safety.

For peer review only

ABSTRACT

Introduction: Deep brain stimulation (DBS) of bilateral anteromedial subthalamic nucleus (STN) has been found to be helpful in a subset of patients with severe, chronic, and treatment-refractory obsessive-compulsive disorder (OCD). Biomarkers may aid in patient selection and optimization of this invasive treatment. In this trial, we intend to evaluate neurocognitive function related to STN and related biosignatures as potential biomarkers for STN-DBS in OCD.

Methods and analysis: Twenty-four subjects with treatment-refractory OCD will undergo open-label STN DBS. Structural/functional imaging, electrophysiological recording, and neurocognitive assessment would be performed at baseline. The subjects would undergo a structured clinical assessment for 12 months post-surgery. A group of 24 healthy volunteers and 24 subjects with treatment-refractory OCD who receive treatment as usual (TAU) would be recruited for comparison of biomarkers and treatment response, respectively. Baseline biomarkers would be evaluated as predictors of clinical response. Neuroadaptive changes would be studied through a reassessment of neurocognitive functioning, imaging, and electrophysiological activity post-DBS.

Ethics and dissemination: The protocol has been approved by the NIMHANS Ethics Committee. The study findings will be disseminated through peer-reviewed scientific journals and scientific meetings.

Keywords: Obsessive-compulsive disorder, treatment-refractory, deep brain stimulation, subthalamic nucleus, biomarkers

STRENGTHS AND LIMITATIONS

- Multimodal evaluation of biomarkers through neuroimaging, scalp/invasive electrophysiological recording and neurocognitive evaluation
- Comparison group of treatment refractory OCD subjects with sufficient follow-up to help evaluating treatment response to DBS
- Limitations include open-label treatment and unblinded assessment.

For peer review only

INTRODUCTION

Obsessive-compulsive disorder (OCD) is a common neuropsychiatric condition with a lifetime prevalence of 2-3%.[1] It is among the top ten causes of neuropsychiatric disabilities worldwide.[2] The first-line treatment of OCD includes selective serotonin reuptake inhibitors (SSRIs) and/or cognitive-behaviour therapy (CBT).[3,4] However, around 20-30% of patients do not respond to standard treatment strategies.[5] Neurosurgical interventions are considered in patients with chronic, severe, and treatment-refractory OCD.[6] Ablative neurosurgical procedures, such as gamma ventral capsulotomy are helpful in around 45-65% of patients with treatment-refractory OCD.[5] Due to the potential irreversible and severe adverse effects associated with ablative procedures, there has been a surge of interest in deep brain stimulation (DBS) as an alternate treatment. DBS involves electrical stimulation of subcortical regions through surgically implanted microelectrodes. High frequency electrical stimulation through these electrodes modulate the activity of dysfunctional neuronal circuits.[7,8] Unlike ablative procedures, the stimulation in DBS can be modulated to optimise improvement and adverse effects.[9,10]

Cortico-striato-thalamo-cortical (CSTC) circuits passing through subcortical regions are implicated in the pathogenesis of OCD.[11,12] Thus, several subcortical structures including anterior limb of the internal capsule, ventral capsule/ventral striatum (VC/VS), nucleus accumbens (NAc), bed nucleus of stria terminalis, anteromedial subthalamic nucleus (amSTN), and inferior thalamic peduncle are targets for DBS in OCD. DBS targeting some of these structures has been found to significantly reduce OCD symptoms as compared to sham stimulation.[13] Based on evidence from a double-blinded randomised controlled trial,[14] a recent treatment guideline recommended bilateral STN DBS for treatment refractory OCD.[15] Two recent randomised crossover trials found equivalent efficacy of DBS targeting amSTN as compared to VC/VS, caudate nucleus and NAc [16,17]. In the later study, most patients, who were masked/blinded to the target of stimulation, preferred the amSTN stimulation based on subjective improvement.[17] A recent systematic review also found similar efficacy for DBS of amSTN and striatal targets.[8] Interestingly, recent evidence suggests that the different targets have similar connections along fronto-subcortical circuits and thus may be targeting the same network.[18,19]

The adverse effects of DBS are frequently stimulation-related and generally mild and reversible.[7,20] DBS is an invasive procedure that requires long term close monitoring for optimization of stimulation as well as periodic changes in battery, which adds to the treatment cost. Despite these limitations, long-term follow-up studies have shown that DBS leads to improvement in symptoms as well as the quality of life.[21,22] However, DBS is helpful in only 60-75% of patients with treatment-refractory OCD.[20,22] There is a need to identify predictors of treatment response, which would assist in patient selection for this invasive and expensive treatment.

Despite decades of research, the exact mechanism of action of DBS is still not clearly understood.[23] Recent evidence suggests that DBS has both local and distant effects with resultant amelioration of pathological network activity.[11,23] The role of target nuclei such as amSTN in the pathophysiology of OCD provides useful clues to unravel the mechanism, which would help identify bio-predictors of response. Further, DBS provides a unique opportunity to study the role of STN in psychopathology with spatial and temporal precision.

Neurocircuitry of OCD

Although the aetiology of OCD is still unknown, converging evidence from neuroimaging studies implicates the dysfunctional CSTC circuits in the pathophysiology of OCD.[11,24] Particularly, the CSTC pathways involving the orbitofrontal cortex (OFC) and, to a lesser

For peer review only

extent the dorsal anterior cingulate cortex (dACC) are dysfunctional in OCD.[25–28] Functional neuroimaging studies show that these cortical regions are hyperactive at rest, which is accentuated during symptoms provocation.[29] Pretreatment OFC and caudate metabolism predict response to medications in OCD.[30,31] Similarly, DBS over various targets modulate the activity in these circuits by increasing their connectivity.[32,33] Thus, modulation of functional connectivity in the CSTC circuits is an important target for anti-obsessive treatments including DBS.

STN in the pathophysiology of OCD

STN is the key basal ganglia input structure of the "hyperdirect" pathways.[34] Based on hyperdirect connectivity, the STN is divided into three partially overlapping sub-territories, namely the limbic, associative and motor regions.[35–37] Computational models and imaging studies suggest that the "hyperdirect" pathway connecting inferior frontal gyrus (IFG) and STN is involved in global response inhibition, for example, during the stop-signal task (SST).[38,39] Functional magnetic resonance imaging (fMRI) studies have shown activation of STN, IFG and pre-supplementary motor area (pre-SMA) during SST performance.[39,40] STN also plays a crucial role in decision making and response selection in conflict situations, by setting a decision threshold that is contextually modulated.[41,42] Recent evidence suggests that neurons in ventromedial STN, which is preferentially connected to dACC and OFC, are especially involved in reactive stopping and switching.[36,43] Both these functions are impaired in patients with OCD.[44,45] Following STN DBS, patients with OCD and Parkinson's disease show decreased reaction time and performance, suggesting more 'impulsivity', as defined in response inhibition paradigms.[46] STN DBS modulates uncertainty/conflict driven decision threshold adjustment and adapting to speed/accuracy trade-offs.[47] Impairment in task switching improves specifically following stimulation of ventral DBS contacts in STN.[48] Thus, DBS targeting amSTN may decrease decisional threshold towards more optimal levels leading to a less cautious and more rapid goal-directed behaviour, which may be beneficial in patients with OCD.[37,49]

STN connectivity has been implicated in the pathophysiology of OCD. Resting-state functional connectivity of STN with cortical and striatal structures have been associated with cognitive and behavioural measures of compulsivity.[50] Tracing studies in non-human primates have found hyperdirect pathways connecting OFC, dACC and dorsolateral prefrontal cortex (DLPFC) projecting to the anteromedial STN [36]. A prospectively acquired imaging study found that effective STN DBS targets in OCD are located in this region i.e. in the anterior-inferior medial border, which has direct connections with the OFC, dACC and DLPFC.[16] Further, evidence suggests that STN DBS modulates cortico-striatal connectivity during SST.[49] STN DBS in OCD decreases metabolism in ACC and the therapeutic effects correlate with a decrease in OFC metabolism.[51] Thus, CSTC networks connecting various prefrontal regions with STN play a role in the neurocognitive functions underlying OCD, its behavioural manifestations and may mediate the therapeutic actions of DBS in OCD. The hyperdirect pathways have especially been implicated in this regard.[52]

Another line of evidence has utilised the unique opportunity provided by DBS to collect electrophysiological data directly from the nucleus. STN oscillatory activity and frontal cortico-STN coherence in β and θ frequency bands are associated with different phases of the SST, with the later prominently seen in the ventral contacts.[53] The medial prefrontal cortex (mPFC)-STN θ-phase coherence increases during high-conflict trials in the Flanker task.[54] Response inhibition and conflict monitoring, assessed through SST and Flanker task respectively, are putative endophenotypes for OCD.[55,56] In OCD subjects, bursting and oscillatory activity have been localised to the associative/limbic STN, with predominant oscillatory activity in δ-band. Further, the severity of OCD is associated with low-frequency oscillatory activity and burst characteristics.[57] Low-frequency activity (θ-band) in the ventro-

For peer review only

medial STN has been associated with symptom provocation and cognitive/emotional functioning in OCD subjects.[58] A recent study demonstrated that emotional images distinctively modulated STN θ-band activity in OCD subjects and emotion-related θ-band activity correlated with symptom severity.[59] Thus, electrophysiological activity in STN, in particular low-frequency oscillatory activity in the ventromedial region, is a putative biomarker associated with neurocognitive functions underlying OCD and its clinical manifestations.

Overall, STN-cortical connectivity (especially with the frontal cortex), scalp electroencephalographic activity in the frontal cortex, bursting/oscillatory activity in the subthalamic nucleus, and STN-related neurocognitive functioning (response inhibition) are potential biomarkers for treatment response to DBS in OCD.

The current proposal plans to identify such biomarkers with the following objectives:

Primary objective:

i. To evaluate whether STN functional connectivity when performing response inhibition task (stop signal task) predicts improvement in obsessive-compulsive symptoms after DBS over STN.

Secondary objectives:

i. To evaluate whether the improvement of obsessive-compulsive symptoms following STN DBS is predicted by
    a. baseline STN resting-state functional connectivity with prefrontal cortex (PFC)
    b. STN functional connectivity with PFC regions during symptom provocation paradigm
    c. White matter structural connectivity (fractional anisotropy and other measures) of STN with cortical regions
    d. Local field potential time frequency amplitude in the STN at rest, during response inhibition task and symptom provocation
    e. Frontal EEG activity at rest, during response inhibition task and symptom provocation.
    f. Localisation of DBS electrodes within the STN

ii. To study the neuroadaptive changes following DBS by evaluating neurocognitive performance and EEG activity pre and 12-months post-DBS.

iii. To evaluate the effect of DBS on clinical symptomatology, disability, quality of life, and subjective well-being.

We hypothesise that baseline STN-prefrontal connectivity, low-frequency oscillatory activity over frontal lobes/STN, and impaired performance on response inhibition task would predict improvement in obsessive-compulsive symptoms after STN DBS in OCD. The current report is prepared based on SPIRIT checklist.[60]

Experimental design:

We plan to conduct a longitudinal open-label follow-up study of 24 subjects undergoing STN DBS STN for severe, chronic and treatment-refractory OCD to evaluate whether baseline biomarkers predict a decrease in the severity of OCD at follow-up. The DBS leads and stimulator would be implanted after baseline investigation for biomarkers. The initial 6 months of follow-up would be utilised for the optimization of stimulus parameters. This would be followed by an open-labelled clinical follow-up of 6 months. The primary outcome would be a clinical improvement after 6 months of stable stimulation i.e. approximately 1 year after the implantation procedure. A comparison group consisting of 24 subjects with chronic, severe, and treatment-refractory OCD, who would receive treatment as usual (TAU), would also be followed-up for 1 year. We would also recruit a sample of 24 healthy volunteers for comparison of biomarkers at baseline (Figure-1).

For peer review only

Sample:

DBS Group (OCD-DBS)

Twenty-four subjects with severe, chronic, and treatment-refractory OCD would be recruited from the OCD clinic, in-patient and out-patient services of the National Institute of Mental Health & Neuro Sciences (NIMHANS), Bangalore. The subjects would be screened with the following selection criteria:

Inclusion criteria

i. Age 18-60 years.
ii. Primary diagnosis of obsessive-compulsive disorder satisfying the DSM-5 criteria,[61] established using Mini International Neuropsychiatric Interview-7.0.2.[62]
iii. Duration of OCD ≥ 5 years.
iv. Yale-Brown Obsessive-Compulsive Scale (YBOCS)[63] score of ≥ 28 or ≥ 14 if the subject has either predominantly obsessions or compulsions alone.
v. Clinical Global Impressions – Severity (CGI-S)[64] scale of ≥ 5
vi. Disability of ≥ 40% as evaluated by the Indian Disability Evaluation and Assessment Scale (IDEAS).[65]
vii. Failure to obtain meaningful improvement in OCD despite adequate trial with standard treatment strategies which should include:
    a. At least 3 trials of serotonin reuptake inhibitors (SRIs), one of which should be clomipramine, which was either ineffective or poorly tolerated. An adequate trial includes the recommended dose of SRIs for ≥ 12 weeks duration each.[6]
    b. Augmentation of SRIs with at least 2 agents for ≥ 6 weeks, one of which should be either risperidone or aripiprazole.
    c. Trial of structured behaviour therapy/cognitive-behaviour therapy for at least 20 sessions or demonstrated an inability to tolerate the anxiety due to therapy.

Exclusion criteria

i. Diagnosis of bipolar disorder, a psychotic disorder of ≥ 3 months duration as assessed with MINI-7.0.2
ii. Current substance use disorder (except caffeine or nicotine use disorder) or Major depressive episode or current high suicidality, as assessed with MINI-7.0.2.
iii. Severe personality disorder as assessed with Structured Clinical Assessment of DSM-5-PD version.
iv. Clinically significant abnormality on magnetic resonance imaging (MRI) of the brain.
v. Pregnancy, contraindication for DBS/anaesthesia/preoperative MRI, inability to comply with surgical requirements.

All potential study participants satisfying the above criteria will be assessed for suitability for DBS by 2 psychiatrists and a neurosurgeon from the study team. An independent review committee with members (consisting of 2 psychiatrists, 1 neurologist, and 1 neurosurgeon) who are not part of the study team would ratify the decision for eligibility for surgery.

OCD control group (OCD-C)

We would recruit 24 OCD subjects fulfilling the above criteria from the same population, including those subjects who refuse consent for DBS. These subjects would receive treatment for OCD as recommended by the treating clinician.

Healthy volunteers (HC)

For peer review only

Twenty-four age and gender-matched healthy volunteers would be recruited from the community to compare the baseline differences in neuroimaging, neurocognitive performance, and electroencephalogram (EEG) with the study subjects. They would be screened for the presence of psychiatric disorder using MINI 7.0.2. Other exclusion criteria include Y-BOCS ≥ 12, family history of psychiatric disorder in first-degree relatives, clinically significant neurological illness, pregnancy, and contraindication for MRI.

Sample size estimation

For evaluating the above-mentioned primary objective, the optimal sample size was estimated using standard principles & methods.[48] Based on approximation & estimation from the previous data on fMRI-based predictors of treatment response for other interventions in OCD (n=12,15),[49,50] a sample size of at least 24 OCD patients will be required to detect a two-tailed significant difference of $\alpha = 0.05$ with an estimated 90% power for an estimated correlation coefficient of 0.6 (Pearson's) between STN-PFC connectivity and change in Y-BOCS total score with add-on DBS.

Baseline assessment

Clinical assessment

The clinical status would be assessed with the MINI-7.0.2.[62] All OCD subjects would undergo baseline assessment with Yale-Brown Obsessive Compulsive Scale (Y-BOCS) which includes a symptom checklist to assess the nature of symptoms, severity scale to assess the severity of symptoms and insight/avoidance scale to assess these domains of symptoms. The severity of illness would also be assessed with the Clinical Global Impressions-Severity (CGI-S) scale. The severity of depressive and anxiety symptoms would be assessed with the Hamilton Depression Rating Scale (HAM-D),[66] and Hamilton Anxiety Rating Scale (HAM-A),[67] respectively. The extent of disability, quality of life and mental wellbeing would be assessed with the WHO Disability Assessment Schedule 2.0- 36 item version (WHODAS 2.0),[68] WHO quality of life instrument (WHOQOL-BREF),[69] and WHO-Five Well-Being Index (WHO-5).[70] The healthy volunteers would undergo screening with MINI-7.0.2 and Y-BOCS.

Neurocognitive assessment

All the subjects would undergo a detailed neurocognitive evaluation consisting of the modified NIMHANS neuropsychology battery,[71] Stop Signal Task (SST),[72] for assessing response inhibition, modified Flanker task,[55] for assessing response inhibition in a conflict situation/error monitoring, Beads task,[37] for assessing decisional impulsivity and temporal discounting task,[73–75] for assessing reward-related decision making. Impairment in the above neurocognitive functions have been observed in OCD patients and they are closely associated with amSTN functioning.[37,43,54–56,76] Besides, Frontotemporal Behavioural Scale,[77] the Iowa Scales for Personality Change,[78] and the UPPS Impulsive Behaviour Scale,[79] would be administered to assess for personality/behavioural dysfunction. Subjects from all three groups would undergo the neurocognitive/behavioural assessment (table-1) at baseline. Besides, OCD subjects would undergo a repeat evaluation at the end of 12 months.

Magnetic resonance imaging

Magnetic resonance imaging (MRI) will be acquired with a 3Tesla MRI scanner (Philips Ingenia) at NIMHANS, Bangalore. Both groups of OCD subjects and healthy volunteers would undergo a baseline structural MRI of the brain and diffusion tensor imaging (DTI) to evaluate the baseline structural connectivity of the STN. Subjects in the OCD-DBS group would undergo an additional baseline structural MRI with a stereotactic frame attached to guide the

For peer review only

accurate targeting of electrodes. All subjects would undergo multi-echo (ME) resting-state functional MRI (fMRI) of the brain to study the functional connectivity of the STN. The subjects would also undergo a ME-fMRI while performing stop-signal task and under symptom provocation (only for the OCD-DBS and OCD-C subjects) employing symptom dimension-specific pictures. ME-fMRI during SST activates the STN and connected cortical regions.[40] Blood oxygenation level-dependent (BOLD) activity in this paradigm would be tested as primary predictors of treatment response to DBS. The symptom provocation paradigm activates cortico-striatal and limbic structures.[80] Activity in these regions has been found to predict response to medications and CBT.[81,82] To the best of our knowledge, ME-fMRI has not been performed under symptom provocation. Hence, it is unclear whether this paradigm would activate STN. Due to the previous data showing activation of OCD specific circuits, activity under this paradigm will be tested as an additional predictor of treatment response.

Electroencephalography

All subjects would undergo an awake 64-channel electroencephalogram (EEG) as per the international 10-20 EEG system electrode placement, in a comfortable resting supine position. Recordings from the frontocentral(FCz), central (Cz), and bilateral dorsolateral prefrontal (F3 & F4) montages would be the focus of analysis. The OCD subjects and healthy volunteers would undergo EEG while performing the SST and flanker tasks. The OCD subjects would also undergo EEG undergo symptom provocation employing symptom dimension-specific pictures. Artefact free epochs of resting-state EEG epochs will be selected for power spectral analysis which involves examining the spectral power in various frequency bands (δ=1-3 Hz, θ = 4-7 Hz, α = 8-11 Hz, β =12-30 Hz, γ >30Hz) at each electrode. For examining the EEG under symptom provocation, power spectral analysis would be conducted on 2-second epochs selected from 1 second before stimuli to 1 second after the stimuli i.e. symptom dimension specific pictures.

Deep Brain stimulation

Following the baseline assessments, subjects in the OCD-DBS will undergo bilateral STN DBS.

Surgical procedure

STN would be located through direct visualisation in MR images. Quadripolar electrodes would be implanted at the boundary of limbic and associative territories of STN, bilaterally under local anaesthesia and antiseptic precautions. The amSTN would be targeted 2 mm anterior and 1 mm medial to the motor STN target used for DBS in Parkinson's disease.[14] The targets would be refined through intra-operative micro-recording along multiple trajectories.[83] Intraoperative macro-stimulation would be conducted to evaluate the acute effects of stimulation. The trajectory would be chosen based on acute adverse vis-a-vis beneficial effects. The position of the electrodes would be confirmed by postoperative MRI using a 3D atlas-MRI co-registration.[84] Electrophysiological activity from STN would be recorded perioperatively and postoperatively. The stimulator would be implanted subcutaneously 5-7 days following STN stimulation after electrophysiological recording.

Electrophysiological recording from STN

Perioperative single-unit recording would be recorded through microelectrodes used for STN localization. Spiking activity would be recorded during 2 minutes of rest and symptom provocation through stereotactically lowered microelectrode leads from various regions of the STN. Symptom provocation would be done by showing individualised symptom provoking pictures. Local field potentials (LFP) would be recorded through the DBS stimulating electrodes postoperatively. LFPs would be recorded through bipolar montages (to reduce

For peer review only - http://bmjopen.bmj.com/site/about/guidelines.xhtml

volume conductance effect) from all adjacent contacts, yielding 3 channels per hemisphere. The electrodes would be externalised through the scalp with extension cables connected to an amplifier for LFP recording. Adequate precautions would be taken to prevent postoperative infection. The LFP recording would be done on the 3rd-5th postoperative days at rest, during SST, Flanker task and under symptom provocation. Power spectral analysis would be performed to study oscillatory activity in various frequency bands (δ=1-3 Hz, θ = 4-7 Hz, α = 8-11 Hz, β =12-30 Hz, γ >30Hz).

Optimization of stimulation parameters

After the implantation of the stimulator, programming would be finalised over the subsequent 6-8 months. The stimulator would be set at a frequency of 130 Hz and pulse width of 60 μs. Successive trials of monopolar stimulation from each contact would be attempted, beginning with the most ventral contact. Voltage would be increased gradually to monitor for adverse effects. Each contact would be evaluated for 6-8 weeks to evaluate therapeutic efficacy. The final contact would be chosen based on a balance between clinical efficacy and adverse effects.

Follow-up intervention

Following the optimisation of parameters that may take up to 6 months post-implantation of stimulator and electrodes, the patient would be followed-up for another 6 months, considering the latency in onset of improvement with DBS in OCD. DBS would be provided as an unblinded add-on treatment. Although the subjects chosen are treatment-refractory, medication changes and CBT would be permitted during the follow-up period due to ethical concerns as well as improve the external validity of the study. CBT would also be permitted during follow-up as it may improve the outcome after DBS.[85]

Subjects in the OCD-C would undergo the best alternative treatment for treatment-refractory OCD based on the standard treatment practices of OCD clinic, NIMHANS, and evidence-based clinical practice guidelines. The participants would be unblinded to the group allotment. If they wish to undergo neurosurgical interventions including DBS for OCD during follow-up, they would be offered the same and would be removed from the control arm of the study.

Follow-up assessments

Clinical assessments

Subjects from both OCD-DBS and OCD-C groups would undergo structured clinical assessment during follow-up. Assessment during follow-up would include a clinical evaluation consisting of monthly assessment of Y-BOCS, CGI-S, HAM-D, and HAM-A (table-1). The subjects would do a self-assessment of their obsessive-compulsive symptoms using a smartphone-based app. WHODAS 2.0, WHOQOL-BREF and WHO-5 would be administered every 3 months. Y-BOCS scores at the end of 6 months of follow-up post-optimisation of stimulation parameters would be the primary outcome variable. The structured assessment would be performed through an in-person interview or video conferencing. Subjects in the OCD-C group would also be assessed with same baseline and follow-up clinical assessments, as specified above.

Neurocognitive assessment, MRI and EEG

At the end of 6 months of stable stimulation (i.e. approximately 12 months post-implantation), both groups of OCD subjects would undergo a detailed neurocognitive assessment as well as EEG (using the same paradigms described above). Subjects in the OCD-DBS group would undergo follow-up EEG both in the stimulation turned "ON" and "OFF" phases on 2

For peer review only

consecutive days. The DBS-artefacts in the follow-up EEG recordings during the "ON" phase would be removed using recommended techniques including oversampling, temporal low-pass filtering and frequency-domain Hampel filtering.[86] The effect of DBS on neurocognitive and EEG markers would be evaluated and correlated with symptom change. Acquiring MRI scans with DBS electrodes in situ may carry the risk of image distortion, interference with DBS stimulator, and risk of tissue damage due to overheating of the electrodes.[87] However, recent reports suggest that MRI scans could be obtained safely, under appropriate precautions.[88–90] DBS manufacturers recommend precautions for acquiring MRI scans in this population, such as acquisition using 1.5 T MRI. Thus, post-surgical MRI scans (including T1-weighted structural images, fMRI and DTI) would be acquired under appropriate precautions, only for subjects who provide informed consent after explaining the risks and benefits associated with the procedure. Subjects in the OCD-C group would undergo follow-up MRI, EEG, and neuro assessments at the end of 12 months, employing the same paradigms and protocols as in the OCD-DBS group. As the subjects would not undergo surgery, the electrophysiological recording would not be obtained from the subthalamic nucleus. The assessment schedule for the 3 groups are is shown in table-2.

Plan for analysis

The OCD-C subjects would serve as a comparator for the intervention group to establish the efficacy of DBS. The baseline and follow-up biomarkers would be compared between the two groups employing the standard statistical techniques mentioned in the protocol. The healthy volunteer group would serve as a comparator to establish the baseline differences in MRI, EEG, and neurocognitive test performances with the OCD subjects.

The statistical analysis would be conducted using the Statistical Package for Social Sciences (SPSS) software (SPSS Inc., Chicago, IL). The fMRI processing and analysis would be conducted using the Statistical Parametric Mapping (SPM) software (http://www.fil.ion.ucl.ac.uk/spm/). Functional connectivity analysis would be conducted using SPM toolboxes and FSL software (https://fsl.fmrib.ox.ac.uk/fsl/fslwiki/). Resting-state fMRI would be analysed using SPM, FSL, DPABI toolbox (http://rfmri.org/dpabi) as per established processing pipelines. DBS electrodes would be localised from postoperative and preoperative images following standardized pipelines using appropriate toolboxes (e.g. Lead-DBS).[91] The structural connectivity of volume of tissue activated (VTA) and location of active electrode within STN would be evaluated as a potential predictor of outcome. EEG analysis and electrophysiological analysis would be conducted using appropriate software. The demographic, clinical (including the principal symptom dimension, age-at-onset etc.), neurocognitive, electrophysiological data will be examined for normality. Correlational followed by multiple regression analysis would be employed to assess whether the biomarkers predict clinical response (Y-BOCS score) at the end of 6 months of stable stimulation Similar analyses would be conducted to test the other study objectives.

Ethical considerations

The study proposal and the informed consent forms have been approved by NIMHANS Ethics Committee [No.NIMHANS/EC(BEH.SC.DIV.) 19TH MEETING 2019 dated 16th July 2019]. The trial has been registered in the Clinical Trial Registry of India (CTRI/2020/03/024131). The subjects would be recruited after obtaining written informed consent explaining the risks and benefits of participating in the study. Subjects in the OCD-DBS group would be diligently educated about and monitored for adverse effects by trained mental health professionals. If subjects from the OCD-C group wish to undergo neurosurgical interventions for OCD during follow-up, they would be offered the same and would be removed from the control arm. Post-operative MRI would be conducted only in consenting subjects from the OCD (intervention) group after explaining the risks and benefits. The subjects in the intervention group would be protected by an insurance plan funded by the research proposal during the process of the

study. A data safety monitoring board would be constituted to periodically review the progress of the study to assess safety issues and recommend continuation, modification, and termination of the trial, as required. All OCD subjects can continue to receive treatment from the OCD clinic, NIMHANS following the study proposal irrespective of whether they complete the study.

Dissemination

The data will be anonymised, curated, and stored in a computer hard disk, with appropriate backup. In keeping with the policies of the funding agency, the data and metadata would be shared with the wider research community through appropriate platforms. The study findings would be disseminated in peer-reviewed scientific publications and scientific conferences, irrespective of outcome.

Expected outcome

Identification of biomarkers of treatment response to bilateral STN DBS in OCD would pave way for personalised medicine by assisting patient selection for this invasive and expensive treatment. Further, this would help understand the mechanisms of action of treatment which would assist in future refinement/optimisation of treatment and open the scope for innovations, including closed-loop stimulation.

References

1 Stein DJ, Costa DLC, Lochner C, *et al.* Obsessive–compulsive disorder. *Nat Rev Dis Primers* 2019;**5**:1–21. doi:10.1038/s41572-019-0102-3

2 Collins PY, Patel V, Joestl SS, *et al.* Grand challenges in global mental health. *Nature* 2011;**475**:27–30. doi:10.1038/475027a

3 Arumugham SS, Reddy YCJ. Commonly asked questions in the treatment of obsessive-compulsive disorder. *Expert Rev Neurother* 2014;**14**:151–63. doi:10.1586/14737175.2014.874287

4 Fineberg NA, Hollander E, Pallanti S, *et al.* Clinical advances in obsessive-compulsive disorder: a position statement by the International College of Obsessive-Compulsive Spectrum Disorders. *Int Clin Psychopharmacol* 2020;**35**:173–93. doi:10.1097/YIC.0000000000000314

5 Bear RE, Fitzgerald P, Rosenfeld JV, *et al.* Neurosurgery for obsessive-compulsive disorder: contemporary approaches. *J Clin Neurosci* 2010;**17**:1–5. doi:10.1016/j.jocn.2009.02.043

6 Janardhan Reddy YC, Sundar AS, Narayanaswamy JC, *et al.* Clinical practice guidelines for Obsessive-Compulsive Disorder. *Indian J Psychiatry* 2017;**59**:S74–90. doi:10.4103/0019-5545.196976

7 Kohl S, Schönherr DM, Luigjes J, *et al.* Deep brain stimulation for treatment-refractory obsessive compulsive disorder: a systematic review. *BMC Psychiatry* 2014;**14**:214. doi:10.1186/s12888-014-0214-y

8 Vicheva P, Butler M, Shotbolt P. Deep brain stimulation for obsessive-compulsive disorder: A systematic review of randomised controlled trials. *Neurosci Biobehav Rev* 2020;**109**:129–38. doi:10.1016/j.neubiorev.2020.01.007

For peer review only

9 Kumar KK, Appelboom G, Lamsam L, *et al.* Comparative effectiveness of neuroablation and deep brain stimulation for treatment-resistant obsessive-compulsive disorder: a meta-analytic study. *J Neurol Neurosurg Psychiatry* 2019;**90**:469–73. doi:10.1136/jnnp-2018-319318

10 Balachander S, Arumugham SS, Srinivas D. Ablative neurosurgery and deep brain stimulation for obsessive-compulsive disorder. *Indian J Psychiatry* 2019;**61**:S77–84. doi:10.4103/psychiatry.IndianJPsychiatry_523_18

11 van den Heuvel OA, van Wingen G, Soriano-Mas C, *et al.* Brain circuitry of compulsivity. *Eur Neuropsychopharmacol* 2016;**26**:810–27. doi:10.1016/j.euroneuro.2015.12.005

12 Dougherty DD, Brennan BP, Stewart SE, *et al.* Neuroscientifically Informed Formulation and Treatment Planning for Patients With Obsessive-Compulsive Disorder: A Review. *JAMA Psychiatry* 2018;**75**:1081–7. doi:10.1001/jamapsychiatry.2018.0930

13 Martinho FP, Duarte GS, Couto FS do. Efficacy, Effect on Mood Symptoms, and Safety of Deep Brain Stimulation in Refractory Obsessive-Compulsive Disorder: A Systematic Review and Meta-Analysis. *The Journal of Clinical Psychiatry* 2020;**81**. doi:10.4088/JCP.19r12821

14 Mallet L, Polosan M, Jaafari N, *et al.* Subthalamic nucleus stimulation in severe obsessive-compulsive disorder. *N Engl J Med* 2008;**359**:2121–34. doi:10.1056/NEJMoa0708514

15 Staudt MD, Pouratian N, Miller JP, *et al.* Congress of Neurological Surgeons Systematic Review and Evidence-Based Guidelines for Deep Brain Stimulations for Obsessive-Compulsive Disorder: Update of the 2014 Guidelines. *Neurosurgery* 2021;**88**:710–2. doi:10.1093/neuros/nyaa596

16 Tyagi H, Apergis-Schoute AM, Akram H, *et al.* A Randomized Trial Directly Comparing Ventral Capsule and Anteromedial Subthalamic Nucleus Stimulation in Obsessive-Compulsive Disorder: Clinical and Imaging Evidence for Dissociable Effects. *Biol Psychiatry* 2019;**85**:726–34. doi:10.1016/j.biopsych.2019.01.017

17 Welter M-L, Santos JFAD, Clair A-H, *et al.* Deep Brain Stimulation of the Subthalamic, Accumbens, or Caudate Nuclei for Patients With Severe Obsessive-Compulsive Disorder: A Randomized Crossover Controlled Study. *Biological Psychiatry* 2020;**0**. doi:10.1016/j.biopsych.2020.07.013

18 Li N, Baldermann JC, Kibleur A, *et al.* A unified connectomic target for deep brain stimulation in obsessive-compulsive disorder. *Nature Communications* 2020;**11**:3364. doi:10.1038/s41467-020-16734-3

19 Haber SN, Yendiki A, Jbabdi S. Four Deep Brain Stimulation Targets for Obsessive-Compulsive Disorder: Are They Different? *Biological Psychiatry* Published Online First: 25 July 2020. doi:10.1016/j.biopsych.2020.06.031

20 Alonso P, Cuadras D, Gabriëls L, *et al.* Deep Brain Stimulation for Obsessive-Compulsive Disorder: A Meta-Analysis of Treatment Outcome and Predictors of Response. *PLoS One* 2015;**10**. doi:10.1371/journal.pone.0133591

21 Ooms P, Mantione M, Figee M, *et al.* Deep brain stimulation for obsessive-compulsive disorders: long-term analysis of quality of life. *J Neurol Neurosurg Psychiatry* 2014;**85**:153–8. doi:10.1136/jnnp-2012-302550

For peer review only

22 Mallet L, Montcel STD, Clair A-H, *et al.* Long-term effects of subthalamic stimulation in Obsessive-Compulsive Disorder: Follow-up of a randomized controlled trial. *Brain Stimulation: Basic, Translational, and Clinical Research in Neuromodulation* 2019;**12**:1080–2. doi:10.1016/j.brs.2019.04.004

23 van Westen M, Rietveld E, Figee M, *et al.* Clinical Outcome and Mechanisms of Deep Brain Stimulation for Obsessive-Compulsive Disorder. *Curr Behav Neurosci Rep* 2015;**2**:41–8. doi:10.1007/s40473-015-0036-3

24 Saxena S, Rauch SL. Functional neuroimaging and the neuroanatomy of obsessive-compulsive disorder. *Psychiatr Clin North Am* 2000;**23**:563–86.

25 Burguière E, Monteiro P, Mallet L, *et al.* Striatal circuits, habits, and implications for obsessive-compulsive disorder. *Current Opinion in Neurobiology* 2015;**30**:59–65. doi:10.1016/j.conb.2014.08.008

26 Burguière E, Monteiro P, Feng G, *et al.* Optogenetic Stimulation of Lateral Orbitofronto-Striatal Pathway Suppresses Compulsive Behaviors. *Science* 2013;**340**:1243–6. doi:10.1126/science.1232380

27 Robbins TW, Vaghi MM, Banca P. Obsessive-Compulsive Disorder: Puzzles and Prospects. *Neuron* 2019;**102**:27–47. doi:10.1016/j.neuron.2019.01.046

28 Ahmari SE, Spellman T, Douglass NL, *et al.* Repeated Cortico-Striatal Stimulation Generates Persistent OCD-Like Behavior. *Science* 2013;**340**:1234–9. doi:10.1126/science.1234733

29 Rotge J-Y, Guehl D, Dilharreguy B, *et al.* Provocation of obsessive-compulsive symptoms: a quantitative voxel-based meta-analysis of functional neuroimaging studies. *J Psychiatry Neurosci* 2008;**33**:405–12.

30 Saxena S, Brody AL, Ho ML, *et al.* Differential Brain Metabolic Predictors of Response to Paroxetine in Obsessive-Compulsive Disorder Versus Major Depression. *Am J Psychiatry* 2003;**160**:522–32. doi:10.1176/appi.ajp.160.3.522

31 Saxena S, Brody AL, Maidment KM, *et al.* Localized orbitofrontal and subcortical metabolic changes and predictors of response to paroxetine treatment in obsessive-compulsive disorder. *Neuropsychopharmacology* 1999;**21**:683–93. doi:10.1016/S0893-133X(99)00082-2

32 Figee M, Luigjes J, Smolders R, *et al.* Deep brain stimulation restores frontostriatal network activity in obsessive-compulsive disorder. *Nat Neurosci* 2013;**16**:386–7. doi:10.1038/nn.3344

33 Millet B, Jaafari N, Polosan M, *et al.* Limbic versus cognitive target for deep brain stimulation in treatment-resistant depression: accumbens more promising than caudate. *Eur Neuropsychopharmacol* 2014;**24**:1229–39. doi:10.1016/j.euroneuro.2014.05.006

34 Mulders AEP, Plantinga BR, Schruers K, *et al.* Deep brain stimulation of the subthalamic nucleus in obsessive-compulsive disorder: Neuroanatomical and pathophysiological considerations. *Eur Neuropsychopharmacol* 2016;**26**:1909–19. doi:10.1016/j.euroneuro.2016.10.011

For peer review only

35 Lambert C, Zrinzo L, Nagy Z, *et al.* Confirmation of functional zones within the human subthalamic nucleus: patterns of connectivity and sub-parcellation using diffusion weighted imaging. *Neuroimage* 2012;**60**:83–94. doi:10.1016/j.neuroimage.2011.11.082

36 Haynes WIA, Haber SN. The organization of prefrontal-subthalamic inputs in primates provides an anatomical substrate for both functional specificity and integration: implications for Basal Ganglia models and deep brain stimulation. *J Neurosci* 2013;**33**:4804–14. doi:10.1523/JNEUROSCI.4674-12.2013

37 Voon V, Droux F, Morris L, *et al.* Decisional impulsivity and the associative-limbic subthalamic nucleus in obsessive-compulsive disorder: stimulation and connectivity. *Brain* 2017;**140**:442–56. doi:10.1093/brain/aww309

38 Wiecki TV, Frank MJ. A computational model of inhibitory control in frontal cortex and basal ganglia. *Psychol Rev* 2013;**120**:329–55. doi:10.1037/a0031542

39 Aron AR, Behrens TE, Smith S, *et al.* Triangulating a cognitive control network using diffusion-weighted magnetic resonance imaging (MRI) and functional MRI. *J Neurosci* 2007;**27**:3743–52. doi:10.1523/JNEUROSCI.0519-07.2007

40 Aron AR, Poldrack RA. Cortical and subcortical contributions to Stop signal response inhibition: role of the subthalamic nucleus. *J Neurosci* 2006;**26**:2424–33. doi:10.1523/JNEUROSCI.4682-05.2006

41 Frank MJ. Hold your horses: a dynamic computational role for the subthalamic nucleus in decision making. *Neural Netw* 2006;**19**:1120–36. doi:10.1016/j.neunet.2006.03.006

42 Herz DM, Little S, Pedrosa DJ, *et al.* Mechanisms Underlying Decision-Making as Revealed by Deep-Brain Stimulation in Patients with Parkinson's Disease. *Curr Biol* 2018;**28**:1169-1178.e6. doi:10.1016/j.cub.2018.02.057

43 Pasquereau B, Turner RS. A selective role for ventromedial subthalamic nucleus in inhibitory control. *eLife Sciences* 2017;**6**:e31627. doi:10.7554/eLife.31627

44 Chamberlain SR, Fineberg NA, Blackwell AD, *et al.* Motor Inhibition and Cognitive Flexibility in Obsessive-Compulsive Disorder and Trichotillomania. *AJP* 2006;**163**:1282–4. doi:10.1176/ajp.2006.163.7.1282

45 Gu B-M, Park J-Y, Kang D-H, *et al.* Neural correlates of cognitive inflexibility during task-switching in obsessive-compulsive disorder. *Brain* 2008;**131**:155–64. doi:10.1093/brain/awm277

46 Ballanger B, van Eimeren T, Moro E, *et al.* Stimulation of the subthalamic nucleus and impulsivity: release your horses. *Ann Neurol* 2009;**66**:817–24. doi:10.1002/ana.21795

47 Green N, Bogacz R, Huebl J, *et al.* Reduction of influence of task difficulty on perceptual decision making by STN deep brain stimulation. *Curr Biol* 2013;**23**:1681–4. doi:10.1016/j.cub.2013.07.001

48 Greenhouse I, Gould S, Houser M, *et al.* Stimulation of contacts in ventral but not dorsal subthalamic nucleus normalizes response switching in Parkinson's disease. *Neuropsychologia* 2013;**51**:1302–9. doi:10.1016/j.neuropsychologia.2013.03.008

For peer review only

49 Kibleur A, Gras-Combe G, Benis D, *et al.* Modulation of motor inhibition by subthalamic stimulation in obsessive-compulsive disorder. *Transl Psychiatry* 2016;**6**:e922. doi:10.1038/tp.2016.192

50 Morris LS, Baek K, Voon V. Distinct cortico-striatal connections with subthalamic nucleus underlie facets of compulsivity. *Cortex* 2017;**88**:143–50. doi:10.1016/j.cortex.2016.12.018

51 Le Jeune F, Vérin M, N'Diaye K, *et al.* Decrease of prefrontal metabolism after subthalamic stimulation in obsessive-compulsive disorder: a positron emission tomography study. *Biol Psychiatry* 2010;**68**:1016–22. doi:10.1016/j.biopsych.2010.06.033

52 Senova S, Clair A-H, Palfi S, *et al.* Deep Brain Stimulation for Refractory Obsessive-Compulsive Disorder: Towards an Individualized Approach. *Front Psychiatry* 2019;**10**. doi:10.3389/fpsyt.2019.00905

53 Alegre M, Lopez-Azcarate J, Obeso I, *et al.* The subthalamic nucleus is involved in successful inhibition in the stop-signal task: A local field potential study in Parkinson's disease. *Experimental Neurology* 2013;**239**:1–12. doi:10.1016/j.expneurol.2012.08.027

54 Zavala B, Tan H, Ashkan K, *et al.* Human subthalamic nucleus–medial frontal cortex theta phase coherence is involved in conflict and error related cortical monitoring. *NeuroImage* 2016;**137**:178–87. doi:10.1016/j.neuroimage.2016.05.031

55 Riesel A, Kathmann N, Endrass T. Overactive performance monitoring in obsessive-compulsive disorder is independent of symptom expression. *Eur Arch Psychiatry Clin Neurosci* 2014;**264**:707–17. doi:10.1007/s00406-014-0499-3

56 Chamberlain SR, Fineberg NA, Menzies LA, *et al.* Impaired Cognitive Flexibility and Motor Inhibition in Unaffected First-Degree Relatives of Patients With Obsessive-Compulsive Disorder. *AJP* 2007;**164**:335–8. doi:10.1176/ajp.2007.164.2.335

57 Welter M-L, Burbaud P, Fernandez-Vidal S, *et al.* Basal ganglia dysfunction in OCD: subthalamic neuronal activity correlates with symptoms severity and predicts high-frequency stimulation efficacy. *Translational Psychiatry* 2011;**1**:e5. doi:10.1038/tp.2011.5

58 Rappel P, Marmor O, Bick AS, *et al.* Subthalamic theta activity: a novel human subcortical biomarker for obsessive compulsive disorder. *Translational Psychiatry* 2018;**8**:1–11. doi:10.1038/s41398-018-0165-z

59 Buot A, Karachi C, Lau B, *et al.* Emotions Modulate Subthalamic Nucleus Activity: New Evidence in Obsessive-Compulsive Disorder and Parkinson's Disease Patients. *Biological Psychiatry: Cognitive Neuroscience and Neuroimaging* Published Online First: 11 August 2020. doi:10.1016/j.bpsc.2020.08.002

60 Chan A-W, Tetzlaff JM, Gøtzsche PC, *et al.* SPIRIT 2013 explanation and elaboration: guidance for protocols of clinical trials. *BMJ* 2013;**346**. doi:10.1136/bmj.e7586

61 American Psychiatric Association. *Diagnostic and Statistical Manual of Mental Disorders*. American Psychiatric Association 2013. http://dsm.psychiatryonline.org/doi/book/10.1176/appi.books.9780890425596 (accessed 24 Nov 2014).

62 Sheehan DV, Lecrubier Y, Sheehan KH, *et al.* The Mini-International Neuropsychiatric Interview (M.I.N.I.): the development and validation of a structured diagnostic psychiatric interview for DSM-IV and ICD-10. *J Clin Psychiatry* 1998;**59 Suppl 20**:22-33;quiz 34-57.

For peer review only - http://bmjopen.bmj.com/site/about/guidelines.xhtml

63 Goodman WK, Price LH, Rasmussen SA, *et al.* The Yale-Brown Obsessive Compulsive Scale. I. Development, use, and reliability. *Arch Gen Psychiatry* 1989;**46**:1006–11.

64 Guy W. ECDEU Assessment Manual for Psychopharmacology —Revised. Rockville, MD: : U.S. Department of Health, Education, and Welfare, Public Health Service, Alcohol, Drug Abuse, and Mental Health Administration, NIMH Psychopharmacology Research Branch, Division of Extramural Research Programs, 1976. 218–22.

65 The Rehabilitation Committee of the Indian Psychiatric Society. IDEAS (Indian Disability Evaluation and Assessment Scale) – A scale for measuring and quantifying disability in mental disorders. Gurgaon, India: : Indian Psychiatric Society

66 Hamilton M. A rating scale for depression. *J Neurol Neurosurg Psychiatr* 1960;**23**:56–62.

67 Hamilton M. The assessment of anxiety states by rating. *Br J Med Psychol* 1959;**32**:50–5.

68 WHO | WHO Disability Assessment Schedule 2.0 (WHODAS 2.0). WHO. http://www.who.int/classifications/icf/whodasii/en/ (accessed 16 May 2018).

69 Skevington SM, Lotfy M, O'Connell KA, *et al.* The World Health Organization's WHOQOL-BREF quality of life assessment: psychometric properties and results of the international field trial. A report from the WHOQOL group. *Qual Life Res* 2004;**13**:299–310. doi:10.1023/B:QURE.0000018486.91360.00

70 Bech P, Olsen LR, Kjoller M, *et al.* Measuring well-being rather than the absence of distress symptoms: a comparison of the SF-36 Mental Health subscale and the WHO-Five Well-Being Scale. *Int J Methods Psychiatr Res* 2003;**12**:85–91.

71 Rao S, Subbakrishna DK, Gopukumar K. *NIMHANS Neuropsychological Battery*. Bangalore, India: : National Institute of Mental Health and Neurosciences 2004.

72 Logan GD, Cowan WB, Davis KA. On the ability to inhibit simple and choice reaction time responses: a model and a method. *J Exp Psychol Hum Percept Perform* 1984;**10**:276–91. doi:10.1037//0096-1523.10.2.276

73 Kable JW, Glimcher PW. The neural correlates of subjective value during intertemporal choice. *Nature Neuroscience* 2007;**10**:1625–33. doi:10.1038/nn2007

74 Figner B, Knoch D, Johnson EJ, *et al.* Lateral prefrontal cortex and self-control in intertemporal choice. *Nature Neuroscience* 2010;**13**:538–9. doi:10.1038/nn.2516

75 Steinglass JE, Lempert KM, Choo T-H, *et al.* Temporal discounting across three psychiatric disorders: Anorexia nervosa, obsessive compulsive disorder, and social anxiety disorder. *Depress Anxiety* 2017;**34**:463–70. doi:10.1002/da.22586

76 Norman LJ, Carlisi CO, Christakou A, *et al.* Neural dysfunction during temporal discounting in paediatric Attention-Deficit/Hyperactivity Disorder and Obsessive-Compulsive Disorder. *Psychiatry Research: Neuroimaging* 2017;**269**:97–105. doi:10.1016/j.pscychresns.2017.09.008

77 Lebert F, Pasquier F, Souliez L, *et al.* Frontotemporal behavioral scale. *Alzheimer Dis Assoc Disord* 1998;**12**:335–9.

For peer review only

78 Barrash J, Anderson S. *The Iowa Scales for Personality Change*. Iowa City: : University of Iowa, Department of Neurology 1997.

79 Whiteside SP, Lynam DR. The Five Factor Model and impulsivity: using a structural model of personality to understand impulsivity. *Personality and Individual Differences* 2001;**30**:669–89. doi:10.1016/S0191-8869(00)00064-7

80 Simon D, Adler N, Kaufmann C, *et al.* Amygdala hyperactivation during symptom provocation in obsessive-compulsive disorder and its modulation by distraction. *Neuroimage Clin* 2014;**4**:549–57. doi:10.1016/j.nicl.2014.03.011

81 Sanematsu H, Nakao T, Yoshiura T, *et al.* Predictors of treatment response to fluvoxamine in obsessive-compulsive disorder: an fMRI study. *J Psychiatr Res* 2010;**44**:193–200. doi:10.1016/j.jpsychires.2009.08.007

82 Olatunji BO, Davis ML, Powers MB, *et al.* Cognitive-behavioral therapy for obsessive-compulsive disorder: a meta-analysis of treatment outcome and moderators. *J Psychiatr Res* 2013;**47**:33–41. doi:10.1016/j.jpsychires.2012.08.020

83 Hutchison WD, Allan RJ, Opitz H, *et al.* Neurophysiological identification of the subthalamic nucleus in surgery for Parkinson's disease. *Ann Neurol* 1998;**44**:622–8. doi:10.1002/ana.410440407

84 Yelnik J, Damier P, Demeret S, *et al.* Localization of stimulating electrodes in patients with Parkinson disease by using a three-dimensional atlas-magnetic resonance imaging coregistration method. *J Neurosurg* 2003;**99**:89–99. doi:10.3171/jns.2003.99.1.0089

85 Mantione M, Nieman DH, Figee M, *et al.* Cognitive-behavioural therapy augments the effects of deep brain stimulation in obsessive-compulsive disorder. *Psychol Med* 2014;**44**:3515–22. doi:10.1017/S0033291714000956

86 Lio G, Thobois S, Ballanger B, *et al.* Removing deep brain stimulation artifacts from the electroencephalogram: Issues, recommendations and an open-source toolbox. *Clinical Neurophysiology* 2018;**129**:2170–85. doi:10.1016/j.clinph.2018.07.023

87 Carmichael DW, Pinto S, Limousin-Dowsey P, *et al.* Functional MRI with active, fully implanted, deep brain stimulation systems: safety and experimental confounds. *Neuroimage* 2007;**37**:508–17. doi:10.1016/j.neuroimage.2007.04.058

88 Bronstein JM, Tagliati M, Alterman RL, *et al.* Deep brain stimulation for Parkinson disease: an expert consensus and review of key issues. *Arch Neurol* 2011;**68**:165. doi:10.1001/archneurol.2010.260

89 Zrinzo L, Yoshida F, Hariz MI, *et al.* Clinical Safety of Brain Magnetic Resonance Imaging with Implanted Deep Brain Stimulation Hardware: Large Case Series and Review of the Literature. *World Neurosurgery* 2011;**76**:164–72. doi:10.1016/j.wneu.2011.02.029

90 Saleh C, Dooms G, Berthold C, *et al.* Post-operative imaging in deep brain stimulation: A controversial issue. *Neuroradiol J* 2016;**29**:244–9. doi:10.1177/1971400916639960

91 Horn A, Li N, Dembek TA, *et al.* Lead-DBS v2: Towards a comprehensive pipeline for deep brain stimulation imaging. *Neuroimage* 2019;**184**:293–316. doi:10.1016/j.neuroimage.2018.08.068

For peer review only

Authors contributions:

SSA, the recipient of the Indian alliance fellowship, conceived and drafted the protocol YCJR advised on study design, clinical phenotyping, funding application and mentored the primary applicant; GVS mentored funding application and mentored the primary applicant, advised on study design, neuroimaging, electrophysiological recording; LM mentored the applicant, is the external sponsor for the fellowship, advised on study design, finalizing biomarkers and DBS targeting/procedure; DS is a collaborator in the project advised on DBS procedures and study design; JCN is a collaborator who worked on the study design, advised on neuroimaging, electroencephalogram, neurocognitive assessment and study design; JTS advised on clinical phenotyping, study design and clinical recruitment, HK advised on neurocognitive assessment. All authors contributed to the refinement of the study protocol and approved the final version.

Funding statement

The study is fully supported by DBT/Wellcome Trust India Alliance Intermediate fellowship granted to SSA (grant number IA/CPHI/18/1/503931). All material/equipment including DBS electrodes would be funded through the fellowship.

Acknowledgement

GV acknowledges the support of the DBT Wellcome Trust India Alliance Clinical/Public Health Research Centre Grant (IA/CRC/19/1/610005).

Competing interests statement: None declared

Patient and public involvement: Patients and public were not involved in the design of this protocol.

Patient consent for publication: Not required

Word count: 3995

For peer review only

Table-1 Neurocognitive & personality assessment

Neurocognitive assessment

1. Modified NIMHANS Neuropsychology battery[71]
    - Finger Tapping
    - Bender Gestalt Test
    - Digit Symbol Substitution Test
    - Colour Trails 1 And 2
    - Block Design test
    - Controlled Oral Word Association Test
    - Animal Names Test
    - Digit Span
    - Spatial Span
    - Tower of London
    - Wisconsin Card Sorting Task
    - Stroop Test
    - Auditory Verbal Learning Test
    - Rey's Complex Figure Test
2. Stop Signal Task[72]
3. Modified Flanker task[55]
4. Beads task[37]
5. Temporal discounting task[73–75]

Personality/Behavioural assessment

1. Frontotemporal Behavioural Scale[77]
2. The Iowa Scales for Personality Change[78]
3. UPPS Impulsive Behaviour Scale[79]

For peer review only

Table-2 Assessment schedule

	**OCD Intervention group**	**OCD control group**	**Healthy volunteers**
MINI 7.0.2	Baseline	Baseline	Baseline
Y-BOCS	Baseline + monthly	Baseline + monthly	Baseline
CGI-S	Baseline + monthly	Baseline + monthly	Baseline
HAM-A	Baseline + monthly	Baseline + monthly	Baseline
HAM-D	Baseline + monthly	Baseline + monthly	Baseline
WHOQOL-BREF	Baseline + 3-monthly	Baseline + 3-monthly	Baseline
WHODAS 2.0	Baseline + 3-monthly	Baseline + 3-monthly	Baseline
WHO-5	Baseline + 3-monthly	Baseline + 3-monthly	Baseline
Neurocognitive testing	Baseline + end of 12 months	Baseline + end of 12 months	Baseline
MRI	Baseline + end of 12 months*	Baseline + end of 12 months	Baseline
EEG	Baseline + end of 12 months	Baseline + end of 12 months	Baseline
Subthalamic nucleus recording	Baseline	**x**	**x**

MINI 7.0.2 Mini International Neuropsychiatric interview 7.0.2,[62] Y-BOCS Yale Brown Obsessive Compulsive Scale,[63] CGI-S Clinical Global Impressions-Severity,[64] HAM-A Hamilton Anxiety Rating Scale,[67] HAM-D Hamilton Depression Rating Scale,[66] WHOQOL-BREF WHO quality of life instrument,[69] WHODAS 2.0 WHO Disability Assessment Schedule,[68] WHO-5 WHO-Five Well-Being Index,[70] MRI – Magnetic Resonance Imaging, EEG - Electroencephalography

Figure 1 Schematic representation of study protocol

All subjects would undergo baseline clinical, neurocognitive, MRI (magnetic resonance imaging) and EEG (electroencephalography) at baseline. For the subjects in the OCD-DBS group i.e. obsessive-compulsive disorder (OCD) subjects who receive DBS, stimulation parameters would be optimised over the next 6 months; followed by for another 6 months of clinical follow-up. Subjects in the OCD-C group i.e. OCD control subjects who receive treatment as usual, would undergo clinical follow-up for 12 months. Both the OCD groups would undergo neurocognitive assessment, MRI and EEG at the end of 12 months. Subjects in HC group (healthy controls) would not undergo any further assessment following baseline.

For peer review only

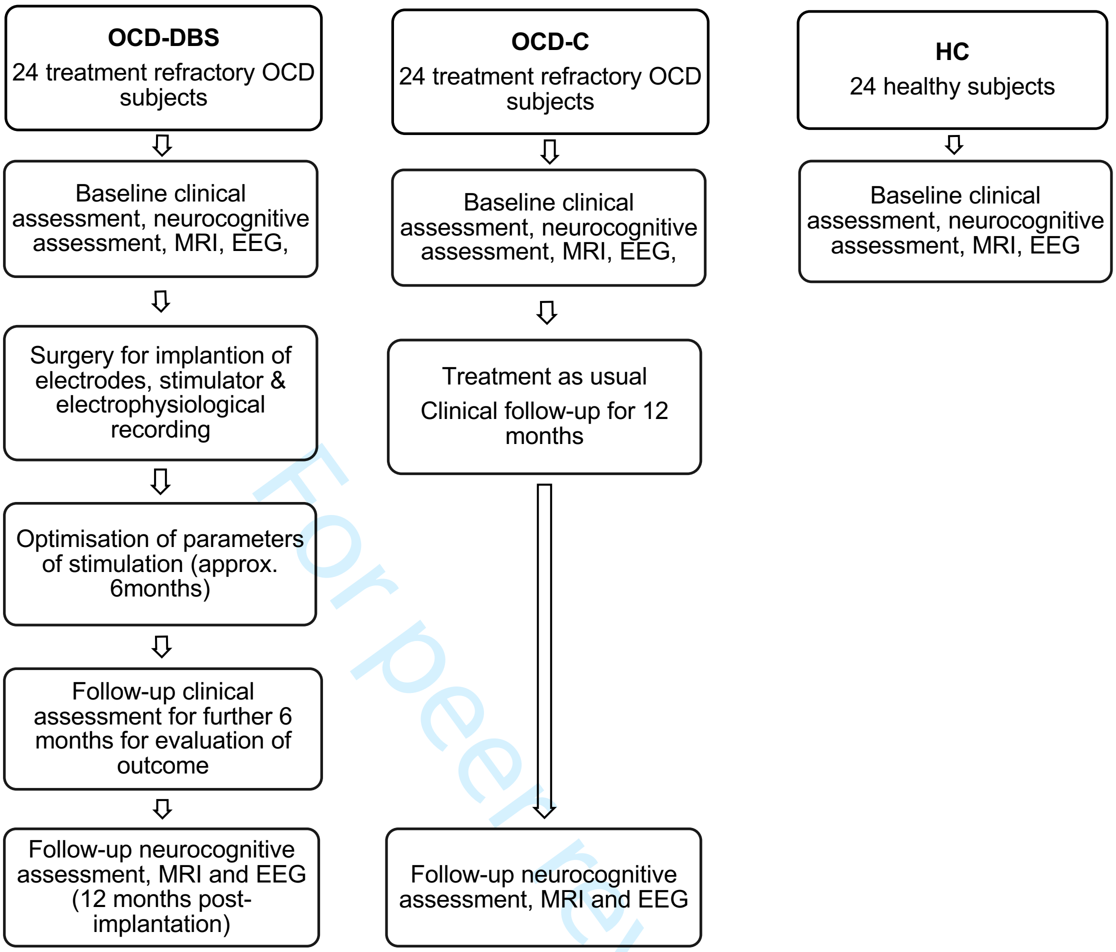

For peer review only

Reporting checklist for protocol of a clinical trial.

Based on the SPIRIT guidelines.

Instructions to authors

Complete this checklist by entering the page numbers from your manuscript where readers will find each of the items listed below.

Your article may not currently address all the items on the checklist. Please modify your text to include the missing information. If you are certain that an item does not apply, please write "n/a" and provide a short explanation.

Upload your completed checklist as an extra file when you submit to a journal.

In your methods section, say that you used the SPIRITreporting guidelines, and cite them as:

Chan A-W, Tetzlaff JM, Gøtzsche PC, Altman DG, Mann H, Berlin J, Dickersin K, Hróbjartsson A, Schulz KF, Parulekar WR, Krleža-Jerić K, Laupacis A, Moher D. SPIRIT 2013 Explanation and Elaboration: Guidance for protocols of clinical trials. BMJ. 2013;346:e7586

		Reporting Item	Page Number
**Administrative information**			
Title	#1	Descriptive title identifying the study design, population, interventions, and, if applicable, trial acronym	1
Trial registration	#2a	Trial identifier and registry name. If not yet registered, name of intended registry	2
Trial registration: data set	#2b	All items from the World Health Organization Trial Registration Data Set	Supp. material-2
Protocol version	#3	Date and version identifier	2
Funding	#4	Sources and types of financial, material, and other support	2
Roles and responsibilities: contributorship	#5a	Names, affiliations, and roles of protocol contributors	2

For peer review only

Roles and responsibilities: sponsor contact information	#5b	Name and contact information for the trial sponsor	4
Roles and responsibilities: sponsor and funder	#5c	Role of study sponsor and funders, if any, in study design; collection, management, analysis, and interpretation of data; writing of the report; and the decision to submit the report for publication, including whether they will have ultimate authority over any of these activities	2
Roles and responsibilities: committees	#5d	Composition, roles, and responsibilities of the coordinating centre, steering committee, endpoint adjudication committee, data management team, and other individuals or groups overseeing the trial, if applicable (see Item 21a for data monitoring committee)	2
**Introduction**			
Background and rationale	#6a	Description of research question and justification for undertaking the trial, including summary of relevant studies (published and unpublished) examining benefits and harms for each intervention	4-5
Background and rationale: choice of comparators	#6b	Explanation for choice of comparators	NA
Objectives	#7	Specific objectives or hypotheses	6
Trial design	#8	Description of trial design including type of trial (eg, parallel group, crossover, factorial, single group), allocation ratio, and framework (eg, superiority, equivalence, non-inferiority, exploratory)	6
**Methods: Participants, interventions, and outcomes**			
Study setting	#9	Description of study settings (eg, community clinic, academic hospital) and list of countries where data will be collected. Reference to where list of study sites can be obtained	6-7
Eligibility criteria	#10	Inclusion and exclusion criteria for participants. If applicable, eligibility criteria for study centres and individuals who will	7

For peer review only

		perform the interventions (eg, surgeons, psychotherapists)	
Interventions: description	#11a	Interventions for each group with sufficient detail to allow replication, including how and when they will be administered	9-10
Interventions: modifications	#11b	Criteria for discontinuing or modifying allocated interventions for a given trial participant (eg, drug dose change in response to harms, participant request, or improving / worsening disease)	9-10
Interventions: adherance	#11c	Strategies to improve adherence to intervention protocols, and any procedures for monitoring adherence (eg, drug tablet return; laboratory tests)	9-10
Interventions: concomitant care	#11d	Relevant concomitant care and interventions that are permitted or prohibited during the trial	9-10
Outcomes	#12	Primary, secondary, and other outcomes, including the specific measurement variable (eg, systolic blood pressure), analysis metric (eg, change from baseline, final value, time to event), method of aggregation (eg, median, proportion), and time point for each outcome. Explanation of the clinical relevance of chosen efficacy and harm outcomes is strongly recommended	10
Participant timeline	#13	Time schedule of enrolment, interventions (including any run-ins and washouts), assessments, and visits for participants. A schematic diagram is highly recommended (see Figure)	10 & figure-1
Sample size	#14	Estimated number of participants needed to achieve study objectives and how it was determined, including clinical and statistical assumptions supporting any sample size calculations	8
Recruitment	#15	Strategies for achieving adequate participant enrolment to reach target sample size	6-7
**Methods: Assignment of interventions (for controlled trials)**			
Allocation: sequence generation	#16a	Method of generating the allocation sequence (eg, computer-generated random numbers), and list of any factors for stratification. To reduce predictability of a random sequence, details of any planned restriction (eg, blocking) should be provided in a separate document that is unavailable to those who enrol participants or assign interventions	NA

For peer review only

Allocation concealment mechanism	#16b	Mechanism of implementing the allocation sequence (eg, central telephone; sequentially numbered, opaque, sealed envelopes), describing any steps to conceal the sequence until interventions are assigned	NA
Allocation: implementation	#16c	Who will generate the allocation sequence, who will enrol participants, and who will assign participants to interventions	NA
Blinding (masking)	#17a	Who will be blinded after assignment to interventions (eg, trial participants, care providers, outcome assessors, data analysts), and how	NA
Blinding (masking): emergency unblinding	#17b	If blinded, circumstances under which unblinding is permissible, and procedure for revealing a participant's allocated intervention during the trial	NA
**Methods: Data collection, management, and analysis**			
Data collection plan	#18a	Plans for assessment and collection of outcome, baseline, and other trial data, including any related processes to promote data quality (eg, duplicate measurements, training of assessors) and a description of study instruments (eg, questionnaires, laboratory tests) along with their reliability and validity, if known. Reference to where data collection forms can be found, if not in the protocol	10,19,20
Data collection plan: retention	#18b	Plans to promote participant retention and complete follow-up, including list of any outcome data to be collected for participants who discontinue or deviate from intervention protocols	10
Data management	#19	Plans for data entry, coding, security, and storage, including any related processes to promote data quality (eg, double data entry; range checks for data values). Reference to where details of data management procedures can be found, if not in the protocol	11
Statistics: outcomes	#20a	Statistical methods for analysing primary and secondary outcomes. Reference to where other details of the statistical analysis plan can be found, if not in the protocol	11
Statistics: additional analyses	#20b	Methods for any additional analyses (eg, subgroup and adjusted analyses)	11

For peer review only

Statistics: analysis population and missing data	#20c	Definition of analysis population relating to protocol non-adherence (eg, as randomised analysis), and any statistical methods to handle missing data (eg, multiple imputation)	11
**Methods: Monitoring**			
Data monitoring: formal committee	#21a	Composition of data monitoring committee (DMC); summary of its role and reporting structure; statement of whether it is independent from the sponsor and competing interests; and reference to where further details about its charter can be found, if not in the protocol. Alternatively, an explanation of why a DMC is not needed	2
Data monitoring: interim analysis	#21b	Description of any interim analyses and stopping guidelines, including who will have access to these interim results and make the final decision to terminate the trial	NA
Harms	#22	Plans for collecting, assessing, reporting, and managing solicited and spontaneously reported adverse events and other unintended effects of trial interventions or trial conduct	11
Auditing	#23	Frequency and procedures for auditing trial conduct, if any, and whether the process will be independent from investigators and the sponsor	NA
**Ethics and dissemination**			
Research ethics approval	#24	Plans for seeking research ethics committee / institutional review board (REC / IRB) approval	11
Protocol amendments	#25	Plans for communicating important protocol modifications (eg, changes to eligibility criteria, outcomes, analyses) to relevant parties (eg, investigators, REC / IRBs, trial participants, trial registries, journals, regulators)	NA
Consent or assent	#26a	Who will obtain informed consent or assent from potential trial participants or authorised surrogates, and how (see Item 32)	11
Consent or assent: ancillary studies	#26b	Additional consent provisions for collection and use of participant data and biological specimens in ancillary studies, if applicable	NA
Confidentiality	#27	How personal information about potential and enrolled participants will be collected, shared, and maintained in order to protect	11

For peer review only

		confidentiality before, during, and after the trial	
Declaration of interests	#28	Financial and other competing interests for principal investigators for the overall trial and each study site	18
Data access	#29	Statement of who will have access to the final trial dataset, and disclosure of contractual agreements that limit such access for investigators	11
Ancillary and post trial care	#30	Provisions, if any, for ancillary and post-trial care, and for compensation to those who suffer harm from trial participation	11
Dissemination policy: trial results	#31a	Plans for investigators and sponsor to communicate trial results to participants, healthcare professionals, the public, and other relevant groups (eg, via publication, reporting in results databases, or other data sharing arrangements), including any publication restrictions	11
Dissemination policy: authorship	#31b	Authorship eligibility guidelines and any intended use of professional writers	NA
Dissemination policy: reproducible research	#31c	Plans, if any, for granting public access to the full protocol, participant-level dataset, and statistical code	11
**Appendices**			
Informed consent materials	#32	Model consent form and other related documentation given to participants and authorised surrogates	Supp. material-1
Biological specimens	#33	Plans for collection, laboratory evaluation, and storage of biological specimens for genetic or molecular analysis in the current trial and for future use in ancillary studies, if applicable	NA

The SPIRIT checklist is distributed under the terms of the Creative Commons Attribution License CC-BY-ND 3.0. This checklist was completed on 27. November 2020 using https://www.goodreports.org/, a tool made by the EQUATOR Network in collaboration with Penelope.ai

For peer review only

BMJ Open

Identification of biomarkers that predict response to subthalamic nucleus deep brain stimulation in resistant obsessive-compulsive disorder: protocol for an open-label follow-up study

Journal:	*BMJ Open*
Manuscript ID	bmjopen-2020-047492.R2
Article Type:	Protocol
Date Submitted by the Author:	24-May-2021
Complete List of Authors:	Arumugham, Shyam Sundar; National Institute of Mental Health and Neuro Sciences, Psychiatry<br>Srinivas, Dwarakanath; National Institute of Mental Health and Neuro Sciences, Neurosurgery<br>Narayanaswamy, Janardhanan ; National Institute of Mental Health and Neuro Sciences, Psychiatry<br>Jaisoorya, TS; National Institute of Mental Health and Neuro Sciences, Psychiatry<br>Kashyap, Himani; National Institute of Mental Health and Neuro Sciences, Clinical Psychology<br>Domenech, Philippe; Hôpitaux Universitaires Henri Mondor, Univ Paris-Est Créteil, DMU CARE - Département Médical-Universitaire de Chirurgie et Anesthésie réanimation, DMU IMPACT, Département Médical-Universitaire de Psychiatrie et d'Addictologie; INSERM U955, Univ of Paris 12 UPEC, Faculté de médecine<br>Palfi, Stéphane; Hôpitaux Universitaires Henri Mondor, Univ Paris-Est Créteil, DMU CARE - Département Médical-Universitaire de Chirurgie et Anesthésie réanimation, DMU IMPACT, Département Médical-Universitaire de Psychiatrie et d'Addictologie; INSERM U955, Univ of Paris 12 UPEC, Faculté de médecine<br>Mallet, Luc; Sorbonne Université, Institut du Cerveau, ICM, Inserm U 1127, CNRS UMR 7225; University of Geneva, Department of Mental Health and Psychiatry<br>Venkatasubramanian, Ganesan; National Institute of Mental Health and Neuro Sciences, Psychiatry<br>Reddy, YC Janardhan; National Institute of Mental Health and Neuro Sciences, Psychiatry
**Primary Subject Heading**:	Mental health
Secondary Subject Heading:	Surgery
Keywords:	PSYCHIATRY, NEUROSURGERY, Anxiety disorders < PSYCHIATRY

I, the Submitting Author has the right to grant and does grant on behalf of all authors of the Work (as defined in the below author licence), an exclusive licence and/or a non-exclusive licence for contributions from authors who are: i) UK Crown employees; ii) where BMJ has agreed a CC-BY licence shall apply, and/or iii) in accordance with the terms applicable for US Federal Government officers or employees acting as part of their official duties; on a worldwide, perpetual, irrevocable, royalty-free basis to BMJ Publishing Group Ltd ("BMJ") its licensees and where the relevant Journal is co-owned by BMJ to the co-owners of the Journal, to publish the Work in this journal and any other BMJ products and to exploit all rights, as set out in our licence.

The Submitting Author accepts and understands that any supply made under these terms is made by BMJ to the Submitting Author unless you are acting as an employee on behalf of your employer or a postgraduate student of an affiliated institution which is paying any applicable article publishing charge ("APC") for Open Access articles. Where the Submitting Author wishes to make the Work available on an Open Access basis (and intends to pay the relevant APC), the terms of reuse of such Open Access shall be governed by a Creative Commons licence – details of these licences and which Creative Commons licence will apply to this Work are set out in our licence referred to above.

Other than as permitted in any relevant BMJ Author's Self Archiving Policies, I confirm this Work has not been accepted for publication elsewhere, is not being considered for publication elsewhere and does not duplicate material already published. I confirm all authors consent to publication of this Work and authorise the granting of this licence.

For peer review only

Identification of biomarkers that predict response to subthalamic nucleus deep brain stimulation in resistant obsessive-compulsive disorder: protocol for an open-label follow-up study

Shyam Sundar Arumugham[1*], Dwarakanath Srinivas[2], Janardhanan C Narayanaswamy[1], Jaisoorya TS[1], Himani Kashyap[3], Philippe Domenech[4,5], Stéphane Palfi[4,5], Luc Mallet[4,6,7], Ganesan Venkatasubramanian[1], YC Janardhan Reddy[1]

[1]Department of Psychiatry, National Institute of Mental health and Neurosciences, India
[2]Department of Neurosurgery, National Institute of Mental health and Neurosciences, India
[3]Department of Clinical Psychology, National Institute of Mental health and Neurosciences, India
[4]Univ Paris-Est Créteil, DMU CARE - Département Médical-Universitaire de Chirurgie et Anesthésie réanimation, DMU IMPACT, Département Médical-Universitaire de Psychiatrie et d'Addictologie, Hôpitaux Universitaires Henri Mondor - Albert Chenevier, Assistance Publique-Hôpitaux de Paris, Créteil, France
[5]U955 INSERM IMRB eq.15, Univ of Paris 12 UPEC, Faculté de médecine, Créteil, F-94010, France
[6]Institut du Cerveau, ICM, Inserm U 1127, CNRS UMR 7225, Sorbonne Université, F-75013, Paris, France
[7]Department of Mental Health and Psychiatry, Global Health Institute, University of Geneva, Geneva, Switzerland

*** Corresponding author**
Shyam Sundar Arumugham
DBT/Wellcome Trust India Alliance Intermediate Fellow
Additional Professor
Department of Psychiatry
National Institute of Mental Health and Neurosciences (NIMHANS)
Bengaluru, India 560029
Email- a.shyamsundar@gmail.com, shyamsundar.a@nimhans.ac.in
Phone- +91-80-26995353

Number of words: 4279
Number of tables: 2
Number of figures: 1

For peer review only

Trial registration: Clinical trials Registry – India (CTRI): CTRI/2020/03/024131

PROTOCOL VERSION

The current manuscript describes the version of the protocol approved by the institute ethics committee of NIMHANS dated 20th July 2019.

FUNDING

The study is fully funded by the DBT/Wellcome Trust India Alliance Intermediate fellowship granted to SSA. All the material/equipment including DBS electrodes would be purchased through funds from the fellowship.

ROLES & RESPONSIBILITIES

A. Contributors

SSA, the recipient of the Indian alliance fellowship, conceived and drafted the protocol YCJR advised on study design, clinical phenotyping, funding application and mentored the primary applicant; GVS mentored funding application and mentored the primary applicant, advised on study design, neuroimaging, electrophysiological recording; LM mentored the applicant, is the external sponsor for the fellowship, advised on study design, finalizing biomarkers and DBS targeting/procedure; PD advised on study design, DBS management, electrophysiological recording, biomarkers and DBS targeting/procedure; DS is a collaborator in the project advised on DBS procedures and study design; JCN is a collaborator who worked on the study design, advised on neuroimaging, electroencephalogram, neurocognitive assessment and study design; JTS advised on clinical phenotyping, study design and clinical recruitment, SP advised on DBS targeting and neurosurgical procedures, HK advised on neurocognitive assessment. All authors contributed to the refinement of the study protocol and approved the final version.

B. Sponsor

National Institute of Mental Health and Neuro Sciences (NIMHANS), Bengaluru, India
Contact: Director, NIMHANS, Hosur Road, Bengaluru, Karnataka, India 560029.
Email: dirstaff@nimhas.ac.in

C. Role of funding source

This funding source has no role in the design of this study and will not have any role during its execution, analyses, interpretation of the data, or decision to submit results

D. Committees

The Data Safety Monitoring Board (DSMB) consisting of independent specialists and a biostatistician will be constituted for overseeing the trial for its safety.

For peer review only

ABSTRACT

Introduction: Deep brain stimulation (DBS) of bilateral anteromedial subthalamic nucleus (STN) has been found to be helpful in a subset of patients with severe, chronic, and treatment-refractory obsessive-compulsive disorder (OCD). Biomarkers may aid in patient selection and optimization of this invasive treatment. In this trial, we intend to evaluate neurocognitive function related to STN and related biosignatures as potential biomarkers for STN-DBS in OCD.

Methods and analysis: Twenty-four subjects with treatment-refractory OCD will undergo open-label STN DBS. Structural/functional imaging, electrophysiological recording, and neurocognitive assessment would be performed at baseline. The subjects would undergo a structured clinical assessment for 12 months post-surgery. A group of 24 healthy volunteers and 24 subjects with treatment-refractory OCD who receive treatment as usual (TAU) would be recruited for comparison of biomarkers and treatment response, respectively. Baseline biomarkers would be evaluated as predictors of clinical response. Neuroadaptive changes would be studied through a reassessment of neurocognitive functioning, imaging, and electrophysiological activity post-DBS.

Ethics and dissemination: The protocol has been approved by the NIMHANS Ethics Committee. The study findings will be disseminated through peer-reviewed scientific journals and scientific meetings.

Keywords: Obsessive-compulsive disorder, treatment-refractory, deep brain stimulation, subthalamic nucleus, biomarkers

STRENGTHS AND LIMITATIONS

- Multimodal evaluation of biomarkers through neuroimaging, scalp/invasive electrophysiological recording and neurocognitive evaluation
- Comparison group of treatment refractory OCD subjects with sufficient follow-up to help evaluating treatment response to DBS
- Limitations include open-label treatment and unblinded assessment.

For peer review only

INTRODUCTION

Obsessive-compulsive disorder (OCD) is a common neuropsychiatric condition with a lifetime prevalence of 2-3%.[1] It is among the top ten causes of neuropsychiatric disabilities worldwide.[2] The first-line treatment of OCD includes selective serotonin reuptake inhibitors (SSRIs) and/or cognitive-behaviour therapy (CBT).[3,4] However, around 20-30% of patients do not respond to standard treatment strategies.[5] Neurosurgical interventions are considered in patients with chronic, severe, and treatment-refractory OCD.[6] Ablative neurosurgical procedures, such as gamma ventral capsulotomy are helpful in around 45-65% of patients with treatment-refractory OCD.[5] Due to the potential irreversible and severe adverse effects associated with ablative procedures, there has been a surge of interest in deep brain stimulation (DBS) as an alternate treatment. DBS involves electrical stimulation of subcortical regions through surgically implanted microelectrodes. High frequency electrical stimulation through these electrodes modulate the activity of dysfunctional neuronal circuits.[7,8] Unlike ablative procedures, the stimulation in DBS can be modulated to optimise improvement and adverse effects.[9,10]

Cortico-striato-thalamo-cortical (CSTC) circuits passing through subcortical regions are implicated in the pathogenesis of OCD.[11,12] Thus, several subcortical structures including anterior limb of the internal capsule, ventral capsule/ventral striatum (VC/VS), nucleus accumbens (NAc), bed nucleus of stria terminalis, anteromedial subthalamic nucleus (amSTN), and inferior thalamic peduncle are targets for DBS in OCD. DBS targeting some of these structures has been found to significantly reduce OCD symptoms as compared to sham stimulation.[13] Based on evidence from a double-blinded randomised controlled trial,[14] a recent treatment guideline recommended bilateral STN DBS for treatment refractory OCD.[15] Two recent randomised crossover trials found equivalent efficacy of DBS targeting amSTN as compared to VC/VS, caudate nucleus and NAc [16,17]. In the later study, most patients, who were masked/blinded to the target of stimulation, preferred the amSTN stimulation based on subjective improvement.[17] A recent systematic review also found similar efficacy for DBS of amSTN and striatal targets.[8] Interestingly, recent evidence suggests that the different targets have similar connections along fronto-subcortical circuits and thus may be targeting the same network.[18,19]

The adverse effects of DBS are frequently stimulation-related and generally mild and reversible.[7,20] DBS is an invasive procedure that requires long term close monitoring for optimization of stimulation as well as periodic changes in battery, which adds to the treatment cost. Despite these limitations, long-term follow-up studies have shown that DBS leads to improvement in symptoms as well as the quality of life.[21,22] However, DBS is helpful in only 60-75% of patients with treatment-refractory OCD.[20,22] There is a need to identify predictors of treatment response, which would assist in patient selection for this invasive and expensive treatment.

Despite decades of research, the exact mechanism of action of DBS is still not clearly understood.[23] Recent evidence suggests that DBS has both local and distant effects with resultant amelioration of pathological network activity.[11,23] The role of target nuclei such as amSTN in the pathophysiology of OCD provides useful clues to unravel the mechanism, which would help identify bio-predictors of response. Further, DBS provides a unique opportunity to study the role of STN in psychopathology with spatial and temporal precision.

Neurocircuitry of OCD

Although the aetiology of OCD is still unknown, converging evidence from neuroimaging studies implicates the dysfunctional CSTC circuits in the pathophysiology of OCD.[11,24]

For peer review only

Particularly, the CSTC pathways involving the orbitofrontal cortex (OFC) and, to a lesser extent the dorsal anterior cingulate cortex (dACC) are dysfunctional in OCD.[25–28] Functional neuroimaging studies show that these cortical regions are hyperactive at rest, which is accentuated during symptoms provocation.[29] Pretreatment OFC and caudate metabolism predict response to medications in OCD.[30,31] Similarly, DBS over various targets modulate the activity in these circuits by increasing their connectivity.[32,33] Thus, modulation of functional connectivity in the CSTC circuits is an important target for anti-obsessive treatments including DBS.

STN in the pathophysiology of OCD

STN is the key basal ganglia input structure of the “hyperdirect” pathways.[34] Based on hyperdirect connectivity, the STN is divided into three partially overlapping sub-territories, namely the limbic, associative and motor regions.[35–37] Computational models and imaging studies suggest that the “hyperdirect” pathway connecting inferior frontal gyrus (IFG) and STN is involved in global response inhibition, for example, during the stop-signal task (SST).[38,39] Functional magnetic resonance imaging (fMRI) studies have shown activation of STN, IFG and pre-supplementary motor area (pre-SMA) during SST performance.[39,40] STN also plays a crucial role in decision making and response selection in conflict situations, by setting a decision threshold that is contextually modulated.[41,42] Recent evidence suggests that neurons in ventromedial STN, which is preferentially connected to dACC and OFC, are especially involved in reactive stopping and switching.[36,43] Both these functions are impaired in patients with OCD.[44,45] Following STN DBS, patients with OCD and Parkinson’s disease show decreased reaction time and performance, suggesting more ‘impulsivity’, as defined in response inhibition paradigms.[46] STN DBS modulates uncertainty/conflict driven decision threshold adjustment and adapting to speed/accuracy trade-offs.[47] Impairment in task switching improves specifically following stimulation of ventral DBS contacts in STN.[48] Thus, DBS targeting amSTN may decrease decisional threshold towards more optimal levels leading to a less cautious and more rapid goal-directed behaviour, which may be beneficial in patients with OCD.[37,49]

STN connectivity has been implicated in the pathophysiology of OCD. Resting-state functional connectivity of STN with cortical and striatal structures have been associated with cognitive and behavioural measures of compulsivity.[50] Tracing studies in non-human primates have found hyperdirect pathways connecting OFC, dACC and dorsolateral prefrontal cortex (DLPFC) projecting to the anteromedial STN [36]. A prospectively acquired imaging study found that effective STN DBS targets in OCD are located in this region i.e. in the anterior-inferior medial border, which has direct connections with the OFC, dACC and DLPFC.[16] Further, evidence suggests that STN DBS modulates cortico-striatal connectivity during SST.[49] STN DBS in OCD decreases metabolism in ACC and the therapeutic effects correlate with a decrease in OFC metabolism.[51] Thus, CSTC networks connecting various prefrontal regions with STN play a role in the neurocognitive functions underlying OCD, its behavioural manifestations and may mediate the therapeutic actions of DBS in OCD. The hyperdirect pathways have especially been implicated in this regard.[52]

Another line of evidence has utilised the unique opportunity provided by DBS to collect electrophysiological data directly from the nucleus. STN oscillatory activity and frontal cortico-STN coherence in β and θ frequency bands are associated with different phases of the SST, with the later prominently seen in the ventral contacts.[53] The medial prefrontal cortex (mPFC)-STN θ-phase coherence increases during high-conflict trials in the Flanker task.[54] Response inhibition and conflict monitoring, assessed through SST and Flanker task respectively, are putative endophenotypes for OCD.[55,56] In OCD subjects, bursting and oscillatory activity have been localised to the associative/limbic STN, with predominant oscillatory activity in δ-band. Further, the severity of OCD is associated with low-frequency

For peer review only - http://bmjopen.bmj.com/site/about/guidelines.xhtml

oscillatory activity and burst characteristics.[57] Low-frequency activity (θ-band) in the ventromedial STN has been associated with symptom provocation and cognitive/emotional functioning in OCD subjects.[58] A recent study demonstrated that emotional images distinctively modulated STN θ-band activity in OCD subjects and emotion-related θ-band activity correlated with symptom severity.[59] Thus, electrophysiological activity in STN, in particular low-frequency oscillatory activity in the ventromedial region, is a putative biomarker associated with neurocognitive functions underlying OCD and its clinical manifestations.

Overall, STN-cortical connectivity (especially with the frontal cortex), scalp electroencephalographic activity in the frontal cortex, bursting/oscillatory activity in the subthalamic nucleus, and STN-related neurocognitive functioning (response inhibition) are potential biomarkers for treatment response to DBS in OCD.

The current proposal plans to identify such biomarkers with the following objectives:

Primary objective:

i. To evaluate whether STN functional connectivity when performing response inhibition task (stop signal task) predicts improvement in obsessive-compulsive symptoms after DBS over STN.

Secondary objectives:

i. To evaluate whether the improvement of obsessive-compulsive symptoms following STN DBS is predicted by
    a. baseline STN resting-state functional connectivity with prefrontal cortex (PFC)
    b. STN functional connectivity with PFC regions during symptom provocation paradigm
    c. White matter structural connectivity (fractional anisotropy and other measures) of STN with cortical regions
    d. Local field potential time frequency amplitude in the STN at rest, during response inhibition task and symptom provocation
    e. Frontal EEG activity at rest, during response inhibition task and symptom provocation.
    f. Localisation of DBS electrodes within the STN

ii. To study the neuroadaptive changes following DBS by evaluating neurocognitive performance and EEG activity pre and 12-months post-DBS.

iii. To evaluate the effect of DBS on clinical symptomatology, disability, quality of life, and subjective well-being.

We hypothesise that baseline STN-prefrontal connectivity, low-frequency oscillatory activity over frontal lobes/STN, and impaired performance on response inhibition task would predict improvement in obsessive-compulsive symptoms after STN DBS in OCD. The current report is prepared based on SPIRIT checklist.[60]

Experimental design:

We plan to conduct a longitudinal open-label follow-up study of 24 subjects undergoing STN DBS STN for severe, chronic and treatment-refractory OCD to evaluate whether baseline biomarkers predict a decrease in the severity of OCD at follow-up. The DBS leads and stimulator would be implanted after baseline investigation for biomarkers. The initial 6 months of follow-up would be utilised for the optimization of stimulus parameters. This would be followed by an open-labelled clinical follow-up of 6 months. The primary outcome would be a clinical improvement after 6 months of stable stimulation i.e. approximately 1 year after the implantation procedure. A comparison group consisting of 24 subjects with chronic, severe, and treatment-refractory OCD, who would receive treatment as usual (TAU), would also be followed-up for 1 year. We would also recruit a sample of 24 healthy volunteers for comparison

For peer review only

of biomarkers at baseline (Figure-1). Subject enrolment is expected to commence in July 2021 and complete by September 2024.

Sample:

DBS Group (OCD-DBS)

Twenty-four subjects with severe, chronic, and treatment-refractory OCD would be recruited from the OCD clinic, in-patient and out-patient services of the National Institute of Mental Health & Neuro Sciences (NIMHANS), Bangalore. The subjects would be screened with the following selection criteria:

Inclusion criteria

i. Age 18-60 years.
ii. Primary diagnosis of obsessive-compulsive disorder satisfying the DSM-5 criteria,[61] established using Mini International Neuropsychiatric Interview-7.0.2.[62]
iii. Duration of OCD ≥ 5 years.
iv. Yale-Brown Obsessive-Compulsive Scale (YBOCS)[63] score of ≥ 28 or ≥ 14 if the subject has either predominantly obsessions or compulsions alone.
v. Clinical Global Impressions – Severity (CGI-S)[64] scale of ≥ 5
vi. Disability of ≥ 40% as evaluated by the Indian Disability Evaluation and Assessment Scale (IDEAS).[65]
vii. Failure to obtain meaningful improvement in OCD despite adequate trial with standard treatment strategies which should include:
    a. At least 3 trials of serotonin reuptake inhibitors (SRIs), one of which should be clomipramine, which was either ineffective or poorly tolerated. An adequate trial includes the recommended dose of SRIs for ≥ 12 weeks duration each.[6]
    b. Augmentation of SRIs with at least 2 agents for ≥ 6 weeks, one of which should be either risperidone or aripiprazole.
    c. Trial of structured behaviour therapy/cognitive-behaviour therapy for at least 20 sessions or demonstrated an inability to tolerate the anxiety due to therapy.

Exclusion criteria

i. Diagnosis of bipolar disorder, a psychotic disorder of ≥ 3 months duration as assessed with MINI-7.0.2
ii. Current substance use disorder (except caffeine or nicotine use disorder) or Major depressive episode or current high suicidality, as assessed with MINI-7.0.2.
iii. Severe personality disorder as assessed with Structured Clinical Assessment of DSM-5-PD version.
iv. Clinically significant abnormality on magnetic resonance imaging (MRI) of the brain.
v. Pregnancy, contraindication for DBS/anaesthesia/preoperative MRI, inability to comply with surgical requirements.

All potential study participants satisfying the above criteria will be assessed for suitability for DBS by 2 psychiatrists and a neurosurgeon from the study team. An independent review committee with members (consisting of 2 psychiatrists, 1 neurologist, and 1 neurosurgeon) who are not part of the study team would ratify the decision for eligibility for surgery.

OCD control group (OCD-C)

We would recruit 24 OCD subjects fulfilling the above criteria from the same population, including those subjects who refuse consent for DBS. These subjects would receive treatment for OCD as recommended by the treating clinician.

For peer review only

Healthy volunteers (HC)

Twenty-four age and gender-matched healthy volunteers would be recruited from the community to compare the baseline differences in neuroimaging, neurocognitive performance, and electroencephalogram (EEG) with the study subjects. They would be screened for the presence of psychiatric disorder using MINI 7.0.2. Other exclusion criteria include Y-BOCS ≥ 12, family history of psychiatric disorder in first-degree relatives, clinically significant neurological illness, pregnancy, and contraindication for MRI.

Sample size estimation

For evaluating the above-mentioned primary objective, the optimal sample size was estimated using standard principles & methods.[48] Based on approximation & estimation from the previous data on fMRI-based predictors of treatment response for other interventions in OCD (n=12,15),[49,50] a sample size of at least 24 OCD patients will be required to detect a two-tailed significant difference of $\alpha = 0.05$ with an estimated 90% power for an estimated correlation coefficient of 0.6 (Pearson's) between STN-PFC connectivity and change in Y-BOCS total score with add-on DBS.

Baseline assessment

Clinical assessment

The clinical status would be assessed with the MINI-7.0.2.[62] All OCD subjects would undergo baseline assessment with Yale-Brown Obsessive Compulsive Scale (Y-BOCS) which includes a symptom checklist to assess the nature of symptoms, severity scale to assess the severity of symptoms and insight/avoidance scale to assess these domains of symptoms. The severity of illness would also be assessed with the Clinical Global Impressions-Severity (CGI-S) scale. The severity of depressive and anxiety symptoms would be assessed with the Hamilton Depression Rating Scale (HAM-D),[66] and Hamilton Anxiety Rating Scale (HAM-A),[67] respectively. The extent of disability, quality of life and mental wellbeing would be assessed with the WHO Disability Assessment Schedule 2.0- 36 item version (WHODAS 2.0),[68] WHO quality of life instrument (WHOQOL-BREF),[69] and WHO-Five Well-Being Index (WHO-5).[70] The healthy volunteers would undergo screening with MINI-7.0.2 and Y-BOCS.

Neurocognitive assessment

All the subjects would undergo a detailed neurocognitive evaluation consisting of the modified NIMHANS neuropsychology battery,[71] Stop Signal Task (SST),[72] for assessing response inhibition, modified Flanker task,[55] for assessing response inhibition in a conflict situation/error monitoring, Beads task,[37,73] for assessing decisional impulsivity and temporal discounting task,[74–76] for assessing reward-related decision making. Impairment in the above neurocognitive functions have been observed in OCD patients and they are closely associated with amSTN functioning.[37,43,54–56,77] Besides, Frontotemporal Behavioural Scale,[78] the Iowa Scales for Personality Change,[79] and the UPPS Impulsive Behaviour Scale,[80] would be administered to assess for personality/behavioural dysfunction. Subjects from all three groups would undergo the neurocognitive/behavioural assessment (table-1) at baseline. Besides, OCD subjects would undergo a repeat evaluation at the end of 12 months.

Magnetic resonance imaging

Magnetic resonance imaging (MRI) will be acquired with a 3Tesla MRI scanner (Philips Ingenia) at NIMHANS, Bangalore. Both groups of OCD subjects and healthy volunteers would

For peer review only

undergo a baseline structural MRI of the brain and diffusion tensor imaging (DTI) to evaluate the baseline structural connectivity of the STN. Subjects in the OCD-DBS group would undergo an additional baseline structural MRI with a stereotactic frame attached to guide the accurate targeting of electrodes. All subjects would undergo multi-echo (ME) resting-state functional MRI (fMRI) of the brain to study the functional connectivity of the STN. The subjects would also undergo a ME-fMRI while performing stop-signal task and under symptom provocation (only for the OCD-DBS and OCD-C subjects) employing symptom dimension-specific pictures. ME-fMRI during SST activates the STN and connected cortical regions.[40] Blood oxygenation level-dependent (BOLD) activity in this paradigm would be tested as primary predictors of treatment response to DBS. The symptom provocation paradigm activates cortico-striatal and limbic structures.[81] Activity in these regions has been found to predict response to medications and CBT.[82,83] To the best of our knowledge, ME-fMRI has not been performed under symptom provocation. Hence, it is unclear whether this paradigm would activate STN. Due to the previous data showing activation of OCD specific circuits, activity under this paradigm will be tested as an additional predictor of treatment response.

Electroencephalography

All subjects would undergo an awake 64-channel electroencephalogram (EEG) as per the international 10-20 EEG system electrode placement, in a comfortable resting supine position. Recordings from the frontocentral(FCz), central (Cz), and bilateral dorsolateral prefrontal (F3 & F4) montages would be the focus of analysis. The OCD subjects and healthy volunteers would undergo EEG while performing the SST and flanker tasks. The OCD subjects would also undergo EEG undergo symptom provocation employing symptom dimension-specific pictures. Artefact free epochs of resting-state EEG epochs will be selected for power spectral analysis which involves examining the spectral power in various frequency bands (δ=1-3 Hz, θ = 4-7 Hz, α = 8-11 Hz, β =12-30 Hz, γ >30Hz) at each electrode. For examining the EEG under symptom provocation, power spectral analysis would be conducted on 2-second epochs selected from 1 second before stimuli to 1 second after the stimuli i.e. symptom dimension specific pictures.

Deep Brain stimulation

Following the baseline assessments, subjects in the OCD-DBS will undergo bilateral STN DBS.

Surgical procedure

STN would be located through direct visualisation in MR images. Quadripolar electrodes would be implanted at the boundary of limbic and associative territories of STN, bilaterally under local anaesthesia and antiseptic precautions. The amSTN would be targeted 2 mm anterior and 1 mm medial to the motor STN target used for DBS in Parkinson's disease.[14] The targets would be refined through intra-operative micro-recording along multiple trajectories.[84] Intraoperative macro-stimulation would be conducted to evaluate the acute effects of stimulation. The trajectory would be chosen based on acute adverse vis-a-vis beneficial effects. The position of the electrodes would be confirmed by postoperative MRI using a 3D atlas-MRI co-registration.[85] Electrophysiological activity from STN would be recorded perioperatively and postoperatively. The stimulator would be implanted subcutaneously 5-7 days following STN stimulation after electrophysiological recording.

Electrophysiological recording from STN

Perioperative single-unit recording would be recorded through microelectrodes used for STN localization. Spiking activity would be recorded during 2 minutes of rest and symptom provocation through stereotactically lowered microelectrode leads from various regions of the

For peer review only

STN. Symptom provocation would be done by showing individualised symptom provoking pictures. Local field potentials (LFP) would be recorded through the DBS stimulating electrodes postoperatively. LFPs would be recorded through bipolar montages (to reduce volume conductance effect) from all adjacent contacts, yielding 3 channels per hemisphere. The electrodes would be externalised through the scalp with extension cables connected to an amplifier for LFP recording. Adequate precautions would be taken to prevent postoperative infection. The LFP recording would be done on the 3rd-5th postoperative days at rest, during SST, Flanker task and under symptom provocation. Power spectral analysis would be performed to study oscillatory activity in various frequency bands (δ=1-3 Hz, θ = 4-7 Hz, α = 8-11 Hz, β =12-30 Hz, γ >30Hz).

Optimization of stimulation parameters

After the implantation of the stimulator, programming would be finalised over the subsequent 6-8 months. The stimulator would be set at a frequency of 130 Hz and pulse width of 60 μs. Successive trials of monopolar stimulation from each contact would be attempted, beginning with the most ventral contact. Voltage would be increased gradually to monitor for adverse effects. Each contact would be evaluated for 6-8 weeks to evaluate therapeutic efficacy. The final contact would be chosen based on a balance between clinical efficacy and adverse effects.

Follow-up intervention

Following the optimisation of parameters that may take up to 6 months post-implantation of stimulator and electrodes, the patient would be followed-up for another 6 months, considering the latency in onset of improvement with DBS in OCD. DBS would be provided as an unblinded add-on treatment. Although the subjects chosen are treatment-refractory, medication changes and CBT would be permitted during the follow-up period due to ethical concerns as well as improve the external validity of the study. CBT would also be permitted during follow-up as it may improve the outcome after DBS.[86]

Subjects in the OCD-C would undergo the best alternative treatment for treatment-refractory OCD based on the standard treatment practices of OCD clinic, NIMHANS, and evidence-based clinical practice guidelines. The participants would be unblinded to the group allotment. If they wish to undergo neurosurgical interventions including DBS for OCD during follow-up, they would be offered the same and would be removed from the control arm of the study.

Follow-up assessments

Clinical assessments

Subjects from both OCD-DBS and OCD-C groups would undergo structured clinical assessment during follow-up. Assessment during follow-up would include a clinical evaluation consisting of monthly assessment of Y-BOCS, CGI-S, HAM-D, and HAM-A (table-1). The subjects would do a self-assessment of their obsessive-compulsive symptoms using a smartphone-based app. WHODAS 2.0, WHOQOL-BREF and WHO-5 would be administered every 3 months. Y-BOCS scores at the end of 6 months of follow-up post-optimisation of stimulation parameters would be the primary outcome variable. The structured assessment would be performed through an in-person interview or video conferencing. Subjects in the OCD-C group would also be assessed with same baseline and follow-up clinical assessments, as specified above.

Neurocognitive assessment, MRI and EEG

For peer review only

At the end of 6 months of stable stimulation (i.e. approximately 12 months post-implantation), both groups of OCD subjects would undergo a detailed neurocognitive assessment as well as EEG (using the same paradigms described above). Subjects in the OCD-DBS group would undergo follow-up EEG both in the stimulation turned “ON” and “OFF” phases on 2 consecutive days. The DBS-artefacts in the follow-up EEG recordings during the “ON” phase would be removed using recommended techniques including oversampling, temporal low-pass filtering and frequency-domain Hampel filtering.[87] The effect of DBS on neurocognitive and EEG markers would be evaluated and correlated with symptom change. Acquiring MRI scans with DBS electrodes in situ may carry the risk of image distortion, interference with DBS stimulator, and risk of tissue damage due to overheating of the electrodes.[88] However, recent reports suggest that MRI scans could be obtained safely, under appropriate precautions.[89–91] DBS manufacturers recommend precautions for acquiring MRI scans in this population, such as acquisition using 1.5 T MRI. Thus, post-surgical MRI scans (including T1-weighted structural images, fMRI and DTI) would be acquired under appropriate precautions, only for subjects who provide informed consent after explaining the risks and benefits associated with the procedure. Subjects in the OCD-C group would undergo follow-up MRI, EEG, and neuro assessments at the end of 12 months, employing the same paradigms and protocols as in the OCD-DBS group. As the subjects would not undergo surgery, the electrophysiological recording would not be obtained from the subthalamic nucleus. The assessment schedule for the 3 groups are is shown in table-2.

Plan for analysis

The OCD-C subjects would serve as a comparator for the intervention group to establish the efficacy of DBS. The baseline and follow-up biomarkers would be compared between the two groups employing the standard statistical techniques mentioned in the protocol. The healthy volunteer group would serve as a comparator to establish the baseline differences in MRI, EEG, and neurocognitive test performances with the OCD subjects.

The statistical analysis would be conducted using the Statistical Package for Social Sciences (SPSS) software (SPSS Inc., Chicago, IL). The fMRI processing and analysis would be conducted using the Statistical Parametric Mapping (SPM) software (http://www.fil.ion.ucl.ac.uk/spm/). Functional connectivity analysis would be conducted using SPM toolboxes and FSL software (https://fsl.fmrib.ox.ac.uk/fsl/fslwiki/). Resting-state fMRI would be analysed using SPM, FSL, DPABI toolbox (http://rfmri.org/dpabi) as per established processing pipelines. DBS electrodes would be localised from postoperative and preoperative images following standardized pipelines using appropriate toolboxes (e.g. Lead-DBS).[92] The structural connectivity of volume of tissue activated (VTA) and location of active electrode within STN would be evaluated as a potential predictor of outcome. EEG analysis and electrophysiological analysis would be conducted using appropriate software. The demographic, clinical (including the principal symptom dimension, age-at-onset etc.), neurocognitive, electrophysiological data will be examined for normality. Correlational followed by multiple regression analysis would be employed to assess whether the biomarkers predict clinical response (Y-BOCS score) at the end of 6 months of stable stimulation Similar analyses would be conducted to test the other study objectives.

Ethical considerations

The study proposal and the informed consent forms have been approved by NIMHANS Ethics Committee [No.NIMHANS/EC(BEH.SC.DIV.) 19TH MEETING 2019 dated 16th July 2019]. The trial has been registered in the Clinical Trial Registry of India (CTRI/2020/03/024131). The subjects would be recruited after obtaining written informed consent explaining the risks and benefits of participating in the study. Subjects in the OCD-DBS group would be diligently educated about and monitored for adverse effects by trained mental health professionals. If subjects from the OCD-C group wish to undergo neurosurgical interventions for OCD during

For peer review only - http://bmjopen.bmj.com/site/about/guidelines.xhtml

follow-up, they would be offered the same and would be removed from the control arm. Post-operative MRI would be conducted only in consenting subjects from the OCD (intervention) group after explaining the risks and benefits. The subjects in the intervention group would be protected by an insurance plan funded by the research proposal during the process of the study. A data safety monitoring board would be constituted to periodically review the progress of the study to assess safety issues and recommend continuation, modification, and termination of the trial, as required. All OCD subjects can continue to receive treatment from the OCD clinic, NIMHANS following the study proposal irrespective of whether they complete the study.

Dissemination

The data will be anonymised, curated, and stored in a computer hard disk, with appropriate backup. In keeping with the policies of the funding agency, the data and metadata would be shared with the wider research community through appropriate platforms. The study findings would be disseminated in peer-reviewed scientific publications and scientific conferences, irrespective of outcome.

Expected outcome

Identification of biomarkers of treatment response to bilateral STN DBS in OCD would pave way for personalised medicine by assisting patient selection for this invasive and expensive treatment. Further, this would help understand the mechanisms of action of treatment which would assist in future refinement/optimisation of treatment and open the scope for innovations, including closed-loop stimulation.

References

1 Stein DJ, Costa DLC, Lochner C, *et al.* Obsessive–compulsive disorder. *Nat Rev Dis Primers* 2019;**5**:1–21. doi:10.1038/s41572-019-0102-3

2 Collins PY, Patel V, Joestl SS, *et al.* Grand challenges in global mental health. *Nature* 2011;**475**:27–30. doi:10.1038/475027a

3 Arumugham SS, Reddy YCJ. Commonly asked questions in the treatment of obsessive-compulsive disorder. *Expert Rev Neurother* 2014;**14**:151–63. doi:10.1586/14737175.2014.874287

4 Fineberg NA, Hollander E, Pallanti S, *et al.* Clinical advances in obsessive-compulsive disorder: a position statement by the International College of Obsessive-Compulsive Spectrum Disorders. *Int Clin Psychopharmacol* 2020;**35**:173–93. doi:10.1097/YIC.0000000000000314

5 Bear RE, Fitzgerald P, Rosenfeld JV, *et al.* Neurosurgery for obsessive-compulsive disorder: contemporary approaches. *J Clin Neurosci* 2010;**17**:1–5. doi:10.1016/j.jocn.2009.02.043

6 Janardhan Reddy YC, Sundar AS, Narayanaswamy JC, *et al.* Clinical practice guidelines for Obsessive-Compulsive Disorder. *Indian J Psychiatry* 2017;**59**:S74–90. doi:10.4103/0019-5545.196976

7 Kohl S, Schönherr DM, Luigjes J, *et al.* Deep brain stimulation for treatment-refractory obsessive compulsive disorder: a systematic review. *BMC Psychiatry* 2014;**14**:214. doi:10.1186/s12888-014-0214-y

For peer review only

8 Vicheva P, Butler M, Shotbolt P. Deep brain stimulation for obsessive-compulsive disorder: A systematic review of randomised controlled trials. *Neurosci Biobehav Rev* 2020;**109**:129–38. doi:10.1016/j.neubiorev.2020.01.007

9 Kumar KK, Appelboom G, Lamsam L, *et al.* Comparative effectiveness of neuroablation and deep brain stimulation for treatment-resistant obsessive-compulsive disorder: a meta-analytic study. *J Neurol Neurosurg Psychiatry* 2019;**90**:469–73. doi:10.1136/jnnp-2018-319318

10 Balachander S, Arumugham SS, Srinivas D. Ablative neurosurgery and deep brain stimulation for obsessive-compulsive disorder. *Indian J Psychiatry* 2019;**61**:S77–84. doi:10.4103/psychiatry.IndianJPsychiatry_523_18

11 van den Heuvel OA, van Wingen G, Soriano-Mas C, *et al.* Brain circuitry of compulsivity. *Eur Neuropsychopharmacol* 2016;**26**:810–27. doi:10.1016/j.euroneuro.2015.12.005

12 Dougherty DD, Brennan BP, Stewart SE, *et al.* Neuroscientifically Informed Formulation and Treatment Planning for Patients With Obsessive-Compulsive Disorder: A Review. *JAMA Psychiatry* 2018;**75**:1081–7. doi:10.1001/jamapsychiatry.2018.0930

13 Martinho FP, Duarte GS, Couto FS do. Efficacy, Effect on Mood Symptoms, and Safety of Deep Brain Stimulation in Refractory Obsessive-Compulsive Disorder: A Systematic Review and Meta-Analysis. *The Journal of Clinical Psychiatry* 2020;**81**. doi:10.4088/JCP.19r12821

14 Mallet L, Polosan M, Jaafari N, *et al.* Subthalamic nucleus stimulation in severe obsessive-compulsive disorder. *N Engl J Med* 2008;**359**:2121–34. doi:10.1056/NEJMoa0708514

15 Staudt MD, Pouratian N, Miller JP, *et al.* Congress of Neurological Surgeons Systematic Review and Evidence-Based Guidelines for Deep Brain Stimulations for Obsessive-Compulsive Disorder: Update of the 2014 Guidelines. *Neurosurgery* 2021;**88**:710–2. doi:10.1093/neuros/nyaa596

16 Tyagi H, Apergis-Schoute AM, Akram H, *et al.* A Randomized Trial Directly Comparing Ventral Capsule and Anteromedial Subthalamic Nucleus Stimulation in Obsessive-Compulsive Disorder: Clinical and Imaging Evidence for Dissociable Effects. *Biol Psychiatry* 2019;**85**:726–34. doi:10.1016/j.biopsych.2019.01.017

17 Welter M-L, Santos JFAD, Clair A-H, *et al.* Deep Brain Stimulation of the Subthalamic, Accumbens, or Caudate Nuclei for Patients With Severe Obsessive-Compulsive Disorder: A Randomized Crossover Controlled Study. *Biological Psychiatry* 2020;**0**. doi:10.1016/j.biopsych.2020.07.013

18 Li N, Baldermann JC, Kibleur A, *et al.* A unified connectomic target for deep brain stimulation in obsessive-compulsive disorder. *Nature Communications* 2020;**11**:3364. doi:10.1038/s41467-020-16734-3

19 Haber SN, Yendiki A, Jbabdi S. Four Deep Brain Stimulation Targets for Obsessive-Compulsive Disorder: Are They Different? *Biological Psychiatry* Published Online First: 25 July 2020. doi:10.1016/j.biopsych.2020.06.031

20 Alonso P, Cuadras D, Gabriëls L, *et al.* Deep Brain Stimulation for Obsessive-Compulsive Disorder: A Meta-Analysis of Treatment Outcome and Predictors of Response. *PLoS One* 2015;**10**. doi:10.1371/journal.pone.0133591

For peer review only

21 Ooms P, Mantione M, Figee M, *et al.* Deep brain stimulation for obsessive-compulsive disorders: long-term analysis of quality of life. *J Neurol Neurosurg Psychiatry* 2014;**85**:153–8. doi:10.1136/jnnp-2012-302550

22 Mallet L, Montcel STD, Clair A-H, *et al.* Long-term effects of subthalamic stimulation in Obsessive-Compulsive Disorder: Follow-up of a randomized controlled trial. *Brain Stimulation: Basic, Translational, and Clinical Research in Neuromodulation* 2019;**12**:1080–2. doi:10.1016/j.brs.2019.04.004

23 van Westen M, Rietveld E, Figee M, *et al.* Clinical Outcome and Mechanisms of Deep Brain Stimulation for Obsessive-Compulsive Disorder. *Curr Behav Neurosci Rep* 2015;**2**:41–8. doi:10.1007/s40473-015-0036-3

24 Saxena S, Rauch SL. Functional neuroimaging and the neuroanatomy of obsessive-compulsive disorder. *Psychiatr Clin North Am* 2000;**23**:563–86.

25 Burguière E, Monteiro P, Mallet L, *et al.* Striatal circuits, habits, and implications for obsessive-compulsive disorder. *Current Opinion in Neurobiology* 2015;**30**:59–65. doi:10.1016/j.conb.2014.08.008

26 Burguière E, Monteiro P, Feng G, *et al.* Optogenetic Stimulation of Lateral Orbitofronto-Striatal Pathway Suppresses Compulsive Behaviors. *Science* 2013;**340**:1243–6. doi:10.1126/science.1232380

27 Robbins TW, Vaghi MM, Banca P. Obsessive-Compulsive Disorder: Puzzles and Prospects. *Neuron* 2019;**102**:27–47. doi:10.1016/j.neuron.2019.01.046

28 Ahmari SE, Spellman T, Douglass NL, *et al.* Repeated Cortico-Striatal Stimulation Generates Persistent OCD-Like Behavior. *Science* 2013;**340**:1234–9. doi:10.1126/science.1234733

29 Rotge J-Y, Guehl D, Dilharreguy B, *et al.* Provocation of obsessive-compulsive symptoms: a quantitative voxel-based meta-analysis of functional neuroimaging studies. *J Psychiatry Neurosci* 2008;**33**:405–12.

30 Saxena S, Brody AL, Ho ML, *et al.* Differential Brain Metabolic Predictors of Response to Paroxetine in Obsessive-Compulsive Disorder Versus Major Depression. *Am J Psychiatry* 2003;**160**:522–32. doi:10.1176/appi.ajp.160.3.522

31 Saxena S, Brody AL, Maidment KM, *et al.* Localized orbitofrontal and subcortical metabolic changes and predictors of response to paroxetine treatment in obsessive-compulsive disorder. *Neuropsychopharmacology* 1999;**21**:683–93. doi:10.1016/S0893-133X(99)00082-2

32 Figee M, Luigjes J, Smolders R, *et al.* Deep brain stimulation restores frontostriatal network activity in obsessive-compulsive disorder. *Nat Neurosci* 2013;**16**:386–7. doi:10.1038/nn.3344

33 Millet B, Jaafari N, Polosan M, *et al.* Limbic versus cognitive target for deep brain stimulation in treatment-resistant depression: accumbens more promising than caudate. *Eur Neuropsychopharmacol* 2014;**24**:1229–39. doi:10.1016/j.euroneuro.2014.05.006

34 Mulders AEP, Plantinga BR, Schruers K, *et al.* Deep brain stimulation of the subthalamic nucleus in obsessive-compulsive disorder: Neuroanatomical and pathophysiological

For peer review only

considerations. *Eur Neuropsychopharmacol* 2016;**26**:1909–19. doi:10.1016/j.euroneuro.2016.10.011

35 Lambert C, Zrinzo L, Nagy Z, *et al.* Confirmation of functional zones within the human subthalamic nucleus: patterns of connectivity and sub-parcellation using diffusion weighted imaging. *Neuroimage* 2012;**60**:83–94. doi:10.1016/j.neuroimage.2011.11.082

36 Haynes WIA, Haber SN. The organization of prefrontal-subthalamic inputs in primates provides an anatomical substrate for both functional specificity and integration: implications for Basal Ganglia models and deep brain stimulation. *J Neurosci* 2013;**33**:4804–14. doi:10.1523/JNEUROSCI.4674-12.2013

37 Voon V, Droux F, Morris L, *et al.* Decisional impulsivity and the associative-limbic subthalamic nucleus in obsessive-compulsive disorder: stimulation and connectivity. *Brain* 2017;**140**:442–56. doi:10.1093/brain/aww309

38 Wiecki TV, Frank MJ. A computational model of inhibitory control in frontal cortex and basal ganglia. *Psychol Rev* 2013;**120**:329–55. doi:10.1037/a0031542

39 Aron AR, Behrens TE, Smith S, *et al.* Triangulating a cognitive control network using diffusion-weighted magnetic resonance imaging (MRI) and functional MRI. *J Neurosci* 2007;**27**:3743–52. doi:10.1523/JNEUROSCI.0519-07.2007

40 Aron AR, Poldrack RA. Cortical and subcortical contributions to Stop signal response inhibition: role of the subthalamic nucleus. *J Neurosci* 2006;**26**:2424–33. doi:10.1523/JNEUROSCI.4682-05.2006

41 Frank MJ. Hold your horses: a dynamic computational role for the subthalamic nucleus in decision making. *Neural Netw* 2006;**19**:1120–36. doi:10.1016/j.neunet.2006.03.006

42 Herz DM, Little S, Pedrosa DJ, *et al.* Mechanisms Underlying Decision-Making as Revealed by Deep-Brain Stimulation in Patients with Parkinson's Disease. *Curr Biol* 2018;**28**:1169-1178.e6. doi:10.1016/j.cub.2018.02.057

43 Pasquereau B, Turner RS. A selective role for ventromedial subthalamic nucleus in inhibitory control. *eLife Sciences* 2017;**6**:e31627. doi:10.7554/eLife.31627

44 Chamberlain SR, Fineberg NA, Blackwell AD, *et al.* Motor Inhibition and Cognitive Flexibility in Obsessive-Compulsive Disorder and Trichotillomania. *AJP* 2006;**163**:1282–4. doi:10.1176/ajp.2006.163.7.1282

45 Gu B-M, Park J-Y, Kang D-H, *et al.* Neural correlates of cognitive inflexibility during task-switching in obsessive-compulsive disorder. *Brain* 2008;**131**:155–64. doi:10.1093/brain/awm277

46 Ballanger B, van Eimeren T, Moro E, *et al.* Stimulation of the subthalamic nucleus and impulsivity: release your horses. *Ann Neurol* 2009;**66**:817–24. doi:10.1002/ana.21795

47 Green N, Bogacz R, Huebl J, *et al.* Reduction of influence of task difficulty on perceptual decision making by STN deep brain stimulation. *Curr Biol* 2013;**23**:1681–4. doi:10.1016/j.cub.2013.07.001

48 Greenhouse I, Gould S, Houser M, *et al.* Stimulation of contacts in ventral but not dorsal subthalamic nucleus normalizes response switching in Parkinson's disease. *Neuropsychologia* 2013;**51**:1302–9. doi:10.1016/j.neuropsychologia.2013.03.008

For peer review only

49 Kibleur A, Gras-Combe G, Benis D, *et al.* Modulation of motor inhibition by subthalamic stimulation in obsessive-compulsive disorder. *Transl Psychiatry* 2016;**6**:e922. doi:10.1038/tp.2016.192

50 Morris LS, Baek K, Voon V. Distinct cortico-striatal connections with subthalamic nucleus underlie facets of compulsivity. *Cortex* 2017;**88**:143–50. doi:10.1016/j.cortex.2016.12.018

51 Le Jeune F, Vérin M, N'Diaye K, *et al.* Decrease of prefrontal metabolism after subthalamic stimulation in obsessive-compulsive disorder: a positron emission tomography study. *Biol Psychiatry* 2010;**68**:1016–22. doi:10.1016/j.biopsych.2010.06.033

52 Senova S, Clair A-H, Palfi S, *et al.* Deep Brain Stimulation for Refractory Obsessive-Compulsive Disorder: Towards an Individualized Approach. *Front Psychiatry* 2019;**10**. doi:10.3389/fpsyt.2019.00905

53 Alegre M, Lopez-Azcarate J, Obeso I, *et al.* The subthalamic nucleus is involved in successful inhibition in the stop-signal task: A local field potential study in Parkinson's disease. *Experimental Neurology* 2013;**239**:1–12. doi:10.1016/j.expneurol.2012.08.027

54 Zavala B, Tan H, Ashkan K, *et al.* Human subthalamic nucleus–medial frontal cortex theta phase coherence is involved in conflict and error related cortical monitoring. *NeuroImage* 2016;**137**:178–87. doi:10.1016/j.neuroimage.2016.05.031

55 Riesel A, Kathmann N, Endrass T. Overactive performance monitoring in obsessive-compulsive disorder is independent of symptom expression. *Eur Arch Psychiatry Clin Neurosci* 2014;**264**:707–17. doi:10.1007/s00406-014-0499-3

56 Chamberlain SR, Fineberg NA, Menzies LA, *et al.* Impaired Cognitive Flexibility and Motor Inhibition in Unaffected First-Degree Relatives of Patients With Obsessive-Compulsive Disorder. *AJP* 2007;**164**:335–8. doi:10.1176/ajp.2007.164.2.335

57 Welter M-L, Burbaud P, Fernandez-Vidal S, *et al.* Basal ganglia dysfunction in OCD: subthalamic neuronal activity correlates with symptoms severity and predicts high-frequency stimulation efficacy. *Translational Psychiatry* 2011;**1**:e5. doi:10.1038/tp.2011.5

58 Rappel P, Marmor O, Bick AS, *et al.* Subthalamic theta activity: a novel human subcortical biomarker for obsessive compulsive disorder. *Translational Psychiatry* 2018;**8**:1–11. doi:10.1038/s41398-018-0165-z

59 Buot A, Karachi C, Lau B, *et al.* Emotions Modulate Subthalamic Nucleus Activity: New Evidence in Obsessive-Compulsive Disorder and Parkinson's Disease Patients. *Biological Psychiatry: Cognitive Neuroscience and Neuroimaging* Published Online First: 11 August 2020. doi:10.1016/j.bpsc.2020.08.002

60 Chan A-W, Tetzlaff JM, Gøtzsche PC, *et al.* SPIRIT 2013 explanation and elaboration: guidance for protocols of clinical trials. *BMJ* 2013;**346**. doi:10.1136/bmj.e7586

61 American Psychiatric Association. *Diagnostic and Statistical Manual of Mental Disorders*. American Psychiatric Association 2013. http://dsm.psychiatryonline.org/doi/book/10.1176/appi.books.9780890425596 (accessed 24 Nov 2014).

62 Sheehan DV, Lecrubier Y, Sheehan KH, *et al.* The Mini-International Neuropsychiatric Interview (M.I.N.I.): the development and validation of a structured diagnostic psychiatric interview for DSM-IV and ICD-10. *J Clin Psychiatry* 1998;**59 Suppl 20**:22-33;quiz 34-57.

For peer review only

63 Goodman WK, Price LH, Rasmussen SA, *et al.* The Yale-Brown Obsessive Compulsive Scale. I. Development, use, and reliability. *Arch Gen Psychiatry* 1989;**46**:1006–11.

64 Guy W. ECDEU Assessment Manual for Psychopharmacology —Revised. Rockville, MD: : U.S. Department of Health, Education, and Welfare, Public Health Service, Alcohol, Drug Abuse, and Mental Health Administration, NIMH Psychopharmacology Research Branch, Division of Extramural Research Programs, 1976. 218–22.

65 The Rehabilitation Committee of the Indian Psychiatric Society. IDEAS (Indian Disability Evaluation and Assessment Scale) – A scale for measuring and quantifying disability in mental disorders. Gurgaon, India: : Indian Psychiatric Society

66 Hamilton M. A rating scale for depression. *J Neurol Neurosurg Psychiatr* 1960;**23**:56–62.

67 Hamilton M. The assessment of anxiety states by rating. *Br J Med Psychol* 1959;**32**:50–5.

68 WHO | WHO Disability Assessment Schedule 2.0 (WHODAS 2.0). WHO. http://www.who.int/classifications/icf/whodasii/en/ (accessed 16 May 2018).

69 Skevington SM, Lotfy M, O'Connell KA, *et al.* The World Health Organization's WHOQOL-BREF quality of life assessment: psychometric properties and results of the international field trial. A report from the WHOQOL group. *Qual Life Res* 2004;**13**:299–310. doi:10.1023/B:QURE.0000018486.91360.00

70 Bech P, Olsen LR, Kjoller M, *et al.* Measuring well-being rather than the absence of distress symptoms: a comparison of the SF-36 Mental Health subscale and the WHO-Five Well-Being Scale. *Int J Methods Psychiatr Res* 2003;**12**:85–91.

71 Rao S, Subbakrishna DK, Gopukumar K. *NIMHANS Neuropsychological Battery*. Bangalore, India: : National Institute of Mental Health and Neurosciences 2004.

72 Logan GD, Cowan WB, Davis KA. On the ability to inhibit simple and choice reaction time responses: a model and a method. *J Exp Psychol Hum Percept Perform* 1984;**10**:276–91. doi:10.1037//0096-1523.10.2.276

73 Jacoby RJ, Abramowitz JS, Buck BE, *et al.* How is the Beads Task related to intolerance of uncertainty in anxiety disorders? *Journal of Anxiety Disorders* 2014;**28**:495–503. doi:10.1016/j.janxdis.2014.05.005

74 Kable JW, Glimcher PW. The neural correlates of subjective value during intertemporal choice. *Nature Neuroscience* 2007;**10**:1625–33. doi:10.1038/nn2007

75 Figner B, Knoch D, Johnson EJ, *et al.* Lateral prefrontal cortex and self-control in intertemporal choice. *Nature Neuroscience* 2010;**13**:538–9. doi:10.1038/nn.2516

76 Steinglass JE, Lempert KM, Choo T-H, *et al.* Temporal discounting across three psychiatric disorders: Anorexia nervosa, obsessive compulsive disorder, and social anxiety disorder. *Depress Anxiety* 2017;**34**:463–70. doi:10.1002/da.22586

77 Norman LJ, Carlisi CO, Christakou A, *et al.* Neural dysfunction during temporal discounting in paediatric Attention-Deficit/Hyperactivity Disorder and Obsessive-Compulsive Disorder. *Psychiatry Research: Neuroimaging* 2017;**269**:97–105. doi:10.1016/j.pscychresns.2017.09.008

For peer review only

78 Lebert F, Pasquier F, Souliez L, *et al.* Frontotemporal behavioral scale. *Alzheimer Dis Assoc Disord* 1998;**12**:335–9.

79 Barrash J, Anderson S. *The Iowa Scales for Personality Change*. Iowa City: : University of Iowa, Department of Neurology 1997.

80 Whiteside SP, Lynam DR. The Five Factor Model and impulsivity: using a structural model of personality to understand impulsivity. *Personality and Individual Differences* 2001;**30**:669–89. doi:10.1016/S0191-8869(00)00064-7

81 Simon D, Adler N, Kaufmann C, *et al.* Amygdala hyperactivation during symptom provocation in obsessive-compulsive disorder and its modulation by distraction. *Neuroimage Clin* 2014;**4**:549–57. doi:10.1016/j.nicl.2014.03.011

82 Sanematsu H, Nakao T, Yoshiura T, *et al.* Predictors of treatment response to fluvoxamine in obsessive-compulsive disorder: an fMRI study. *J Psychiatr Res* 2010;**44**:193–200. doi:10.1016/j.jpsychires.2009.08.007

83 Olatunji BO, Davis ML, Powers MB, *et al.* Cognitive-behavioral therapy for obsessive-compulsive disorder: a meta-analysis of treatment outcome and moderators. *J Psychiatr Res* 2013;**47**:33–41. doi:10.1016/j.jpsychires.2012.08.020

84 Hutchison WD, Allan RJ, Opitz H, *et al.* Neurophysiological identification of the subthalamic nucleus in surgery for Parkinson's disease. *Ann Neurol* 1998;**44**:622–8. doi:10.1002/ana.410440407

85 Yelnik J, Damier P, Demeret S, *et al.* Localization of stimulating electrodes in patients with Parkinson disease by using a three-dimensional atlas-magnetic resonance imaging coregistration method. *J Neurosurg* 2003;**99**:89–99. doi:10.3171/jns.2003.99.1.0089

86 Mantione M, Nieman DH, Figee M, *et al.* Cognitive-behavioural therapy augments the effects of deep brain stimulation in obsessive-compulsive disorder. *Psychol Med* 2014;**44**:3515–22. doi:10.1017/S0033291714000956

87 Lio G, Thobois S, Ballanger B, *et al.* Removing deep brain stimulation artifacts from the electroencephalogram: Issues, recommendations and an open-source toolbox. *Clinical Neurophysiology* 2018;**129**:2170–85. doi:10.1016/j.clinph.2018.07.023

88 Carmichael DW, Pinto S, Limousin-Dowsey P, *et al.* Functional MRI with active, fully implanted, deep brain stimulation systems: safety and experimental confounds. *Neuroimage* 2007;**37**:508–17. doi:10.1016/j.neuroimage.2007.04.058

89 Bronstein JM, Tagliati M, Alterman RL, *et al.* Deep brain stimulation for Parkinson disease: an expert consensus and review of key issues. *Arch Neurol* 2011;**68**:165. doi:10.1001/archneurol.2010.260

90 Zrinzo L, Yoshida F, Hariz MI, *et al.* Clinical Safety of Brain Magnetic Resonance Imaging with Implanted Deep Brain Stimulation Hardware: Large Case Series and Review of the Literature. *World Neurosurgery* 2011;**76**:164–72. doi:10.1016/j.wneu.2011.02.029

91 Saleh C, Dooms G, Berthold C, *et al.* Post-operative imaging in deep brain stimulation: A controversial issue. *Neuroradiol J* 2016;**29**:244–9. doi:10.1177/1971400916639960

For peer review only

92 Horn A, Li N, Dembek TA, *et al.* Lead-DBS v2: Towards a comprehensive pipeline for deep brain stimulation imaging. *Neuroimage* 2019;**184**:293–316. doi:10.1016/j.neuroimage.2018.08.068

Funding statement

The study is fully supported by DBT/Wellcome Trust India Alliance Intermediate fellowship granted to SSA (grant number IA/CPHI/18/1/503931). All material/equipment including DBS electrodes would be funded through the fellowship.

Acknowledgement

GV acknowledges the support of the DBT Wellcome Trust India Alliance Clinical/Public Health Research Centre Grant (IA/CRC/19/1/610005).

Competing interests statement: None declared

Patient and public involvement: Patients and public were not involved in the design of this protocol.

Patient consent for publication: Not required

Word count: 4279

For peer review only

Table-1 Neurocognitive & personality assessment

Neurocognitive assessment

1. Modified NIMHANS Neuropsychology battery[71]
    - Finger Tapping
    - Bender Gestalt Test
    - Digit Symbol Substitution Test
    - Colour Trails 1 And 2
    - Block Design test
    - Controlled Oral Word Association Test
    - Animal Names Test
    - Digit Span
    - Spatial Span
    - Tower of London
    - Wisconsin Card Sorting Task
    - Stroop Test
    - Auditory Verbal Learning Test
    - Rey's Complex Figure Test
2. Stop Signal Task[72]
3. Modified Flanker task[55]
4. Beads task[37]
5. Temporal discounting task[74–76]

Personality/Behavioural assessment

1. Frontotemporal Behavioural Scale[78]
2. The Iowa Scales for Personality Change[79]
3. UPPS Impulsive Behaviour Scale[80]

For peer review only

Table-2 Assessment schedule

	OCD Intervention group	OCD control group	Healthy volunteers
MINI 7.0.2	Baseline	Baseline	Baseline
Y-BOCS	Baseline + monthly	Baseline + monthly	Baseline
CGI-S	Baseline + monthly	Baseline + monthly	Baseline
HAM-A	Baseline + monthly	Baseline + monthly	Baseline
HAM-D	Baseline + monthly	Baseline + monthly	Baseline
WHOQOL-BREF	Baseline + 3-monthly	Baseline + 3-monthly	Baseline
WHODAS 2.0	Baseline + 3-monthly	Baseline + 3-monthly	Baseline
WHO-5	Baseline + 3-monthly	Baseline + 3-monthly	Baseline
Neurocognitive testing	Baseline + end of 12 months	Baseline + end of 12 months	Baseline
MRI	Baseline + end of 12 months*	Baseline + end of 12 months	Baseline
EEG	Baseline + end of 12 months	Baseline + end of 12 months	Baseline
Subthalamic nucleus recording	Baseline	**x**	**x**

MINI 7.0.2 Mini International Neuropsychiatric interview 7.0.2,[62] Y-BOCS Yale Brown Obsessive Compulsive Scale,[63] CGI-S Clinical Global Impressions-Severity,[64] HAM-A Hamilton Anxiety Rating Scale,[67] HAM-D Hamilton Depression Rating Scale,[66] WHOQOL-BREF WHO quality of life instrument,[69] WHODAS 2.0 WHO Disability Assessment Schedule,[68] WHO-5 WHO-Five Well-Being Index,[70] MRI – Magnetic Resonance Imaging, EEG - Electroencephalography

Figure 1 Schematic representation of study protocol

All subjects would undergo baseline clinical, neurocognitive, MRI (magnetic resonance imaging) and EEG (electroencephalography) at baseline. For the subjects in the OCD-DBS group i.e. obsessive-compulsive disorder (OCD) subjects who receive DBS, stimulation parameters would be optimised over the next 6 months; followed by for another 6 months of clinical follow-up. Subjects in the OCD-C group i.e. OCD control subjects who receive treatment as usual, would undergo clinical follow-up for 12 months. Both the OCD groups would undergo neurocognitive assessment, MRI and EEG at the end of 12 months. Subjects in HC group (healthy controls) would not undergo any further assessment following baseline.

For peer review only

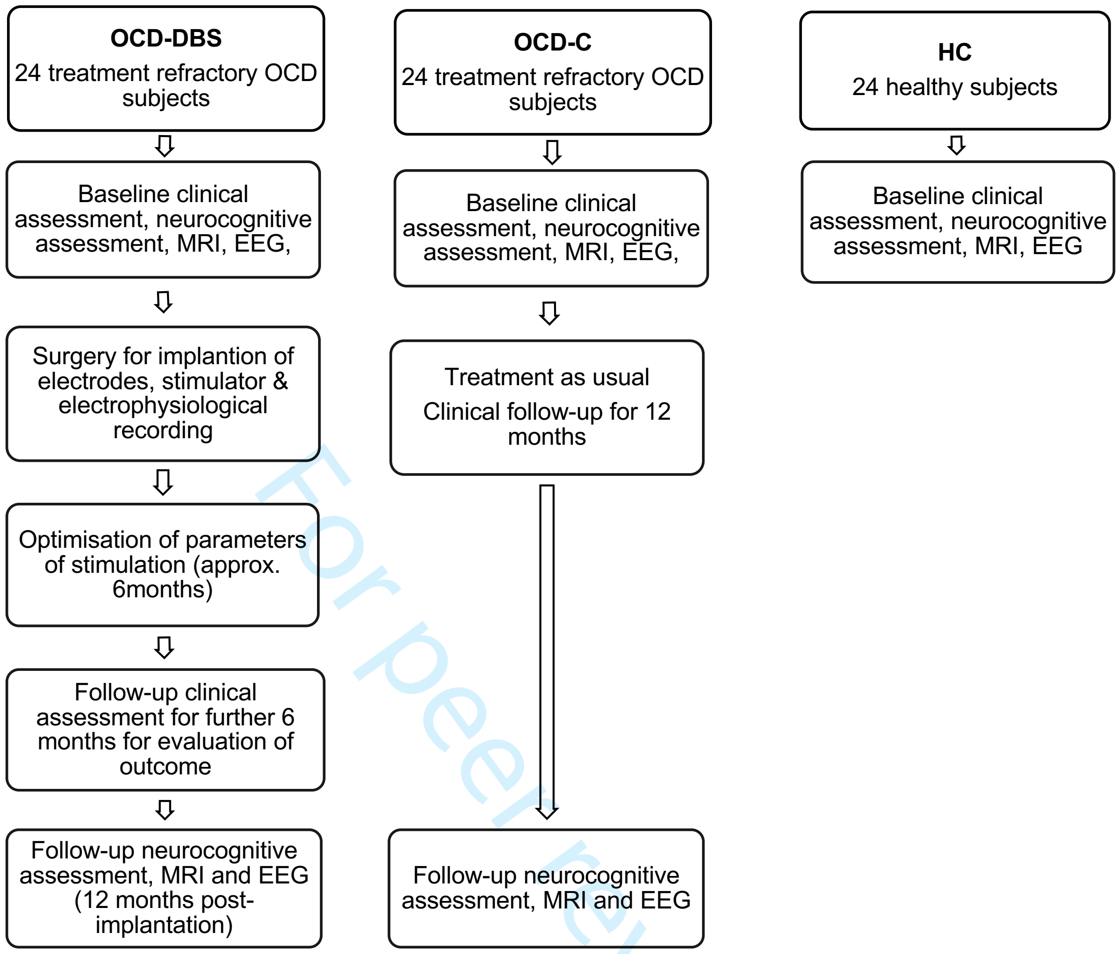
OCD-DBS
24 treatment refractory OCD subjects
Baseline clinical assessment, neurocognitive assessment, MRI, EEG,
Surgery for implantion of electrodes, stimulator & electrophysiological recording
Optimisation of parameters of stimulation (approx. 6months)
Follow-up clinical assessment for further 6 months for evaluation of outcome
Follow-up neurocognitive assessment, MRI and EEG (12 months post-implantation)
OCD-C
24 treatment refractory OCD subjects
Baseline clinical assessment, neurocognitive assessment, MRI, EEG,
Treatment as usual
Clinical follow-up for 12 months
Follow-up neurocognitive assessment, MRI and EEG
HC
24 healthy subjects
Baseline clinical assessment, neurocognitive assessment, MRI, EEG

Reporting checklist for protocol of a clinical trial.

Based on the SPIRIT guidelines.

Instructions to authors

Complete this checklist by entering the page numbers from your manuscript where readers will find each of the items listed below.

Your article may not currently address all the items on the checklist. Please modify your text to include the missing information. If you are certain that an item does not apply, please write "n/a" and provide a short explanation.

Upload your completed checklist as an extra file when you submit to a journal.

In your methods section, say that you used the SPIRITreporting guidelines, and cite them as:

Chan A-W, Tetzlaff JM, Gøtzsche PC, Altman DG, Mann H, Berlin J, Dickersin K, Hróbjartsson A, Schulz KF, Parulekar WR, Krleža-Jerić K, Laupacis A, Moher D. SPIRIT 2013 Explanation and Elaboration: Guidance for protocols of clinical trials. BMJ. 2013;346:e7586

		Reporting Item	Page Number
**Administrative information**			
Title	#1	Descriptive title identifying the study design, population, interventions, and, if applicable, trial acronym	1
Trial registration	#2a	Trial identifier and registry name. If not yet registered, name of intended registry	2
Trial registration: data set	#2b	All items from the World Health Organization Trial Registration Data Set	Supp. material-2
Protocol version	#3	Date and version identifier	2
Funding	#4	Sources and types of financial, material, and other support	2
Roles and responsibilities: contributorship	#5a	Names, affiliations, and roles of protocol contributors	2

For peer review only

Roles and responsibilities: sponsor contact information	#5b	Name and contact information for the trial sponsor	4
Roles and responsibilities: sponsor and funder	#5c	Role of study sponsor and funders, if any, in study design; collection, management, analysis, and interpretation of data; writing of the report; and the decision to submit the report for publication, including whether they will have ultimate authority over any of these activities	2
Roles and responsibilities: committees	#5d	Composition, roles, and responsibilities of the coordinating centre, steering committee, endpoint adjudication committee, data management team, and other individuals or groups overseeing the trial, if applicable (see Item 21a for data monitoring committee)	2
**Introduction**			
Background and rationale	#6a	Description of research question and justification for undertaking the trial, including summary of relevant studies (published and unpublished) examining benefits and harms for each intervention	4-5
Background and rationale: choice of comparators	#6b	Explanation for choice of comparators	NA
Objectives	#7	Specific objectives or hypotheses	6
Trial design	#8	Description of trial design including type of trial (eg, parallel group, crossover, factorial, single group), allocation ratio, and framework (eg, superiority, equivalence, non-inferiority, exploratory)	6
**Methods: Participants, interventions, and outcomes**			
Study setting	#9	Description of study settings (eg, community clinic, academic hospital) and list of countries where data will be collected. Reference to where list of study sites can be obtained	6-7
Eligibility criteria	#10	Inclusion and exclusion criteria for participants. If applicable, eligibility criteria for study centres and individuals who will	7

For peer review only

		perform the interventions (eg, surgeons, psychotherapists)	
Interventions: description	#11a	Interventions for each group with sufficient detail to allow replication, including how and when they will be administered	9-10
Interventions: modifications	#11b	Criteria for discontinuing or modifying allocated interventions for a given trial participant (eg, drug dose change in response to harms, participant request, or improving / worsening disease)	9-10
Interventions: adherance	#11c	Strategies to improve adherence to intervention protocols, and any procedures for monitoring adherence (eg, drug tablet return; laboratory tests)	9-10
Interventions: concomitant care	#11d	Relevant concomitant care and interventions that are permitted or prohibited during the trial	9-10
Outcomes	#12	Primary, secondary, and other outcomes, including the specific measurement variable (eg, systolic blood pressure), analysis metric (eg, change from baseline, final value, time to event), method of aggregation (eg, median, proportion), and time point for each outcome. Explanation of the clinical relevance of chosen efficacy and harm outcomes is strongly recommended	10
Participant timeline	#13	Time schedule of enrolment, interventions (including any run-ins and washouts), assessments, and visits for participants. A schematic diagram is highly recommended (see Figure)	10 & figure-1
Sample size	#14	Estimated number of participants needed to achieve study objectives and how it was determined, including clinical and statistical assumptions supporting any sample size calculations	8
Recruitment	#15	Strategies for achieving adequate participant enrolment to reach target sample size	6-7
**Methods: Assignment of interventions (for controlled trials)**			
Allocation: sequence generation	#16a	Method of generating the allocation sequence (eg, computer-generated random numbers), and list of any factors for stratification. To reduce predictability of a random sequence, details of any planned restriction (eg, blocking) should be provided in a separate document that is unavailable to those who enrol participants or assign interventions	NA

For peer review only

Allocation concealment mechanism	#16b	Mechanism of implementing the allocation sequence (eg, central telephone; sequentially numbered, opaque, sealed envelopes), describing any steps to conceal the sequence until interventions are assigned	NA
Allocation: implementation	#16c	Who will generate the allocation sequence, who will enrol participants, and who will assign participants to interventions	NA
Blinding (masking)	#17a	Who will be blinded after assignment to interventions (eg, trial participants, care providers, outcome assessors, data analysts), and how	NA
Blinding (masking): emergency unblinding	#17b	If blinded, circumstances under which unblinding is permissible, and procedure for revealing a participant's allocated intervention during the trial	NA
**Methods: Data collection, management, and analysis**			
Data collection plan	#18a	Plans for assessment and collection of outcome, baseline, and other trial data, including any related processes to promote data quality (eg, duplicate measurements, training of assessors) and a description of study instruments (eg, questionnaires, laboratory tests) along with their reliability and validity, if known. Reference to where data collection forms can be found, if not in the protocol	10,19,20
Data collection plan: retention	#18b	Plans to promote participant retention and complete follow-up, including list of any outcome data to be collected for participants who discontinue or deviate from intervention protocols	10
Data management	#19	Plans for data entry, coding, security, and storage, including any related processes to promote data quality (eg, double data entry; range checks for data values). Reference to where details of data management procedures can be found, if not in the protocol	11
Statistics: outcomes	#20a	Statistical methods for analysing primary and secondary outcomes. Reference to where other details of the statistical analysis plan can be found, if not in the protocol	11
Statistics: additional analyses	#20b	Methods for any additional analyses (eg, subgroup and adjusted analyses)	11

For peer review only

Statistics: analysis population and missing data	#20c	Definition of analysis population relating to protocol non-adherence (eg, as randomised analysis), and any statistical methods to handle missing data (eg, multiple imputation)	11
**Methods: Monitoring**			
Data monitoring: formal committee	#21a	Composition of data monitoring committee (DMC); summary of its role and reporting structure; statement of whether it is independent from the sponsor and competing interests; and reference to where further details about its charter can be found, if not in the protocol. Alternatively, an explanation of why a DMC is not needed	2
Data monitoring: interim analysis	#21b	Description of any interim analyses and stopping guidelines, including who will have access to these interim results and make the final decision to terminate the trial	NA
Harms	#22	Plans for collecting, assessing, reporting, and managing solicited and spontaneously reported adverse events and other unintended effects of trial interventions or trial conduct	11
Auditing	#23	Frequency and procedures for auditing trial conduct, if any, and whether the process will be independent from investigators and the sponsor	NA
**Ethics and dissemination**			
Research ethics approval	#24	Plans for seeking research ethics committee / institutional review board (REC / IRB) approval	11
Protocol amendments	#25	Plans for communicating important protocol modifications (eg, changes to eligibility criteria, outcomes, analyses) to relevant parties (eg, investigators, REC / IRBs, trial participants, trial registries, journals, regulators)	NA
Consent or assent	#26a	Who will obtain informed consent or assent from potential trial participants or authorised surrogates, and how (see Item 32)	11
Consent or assent: ancillary studies	#26b	Additional consent provisions for collection and use of participant data and biological specimens in ancillary studies, if applicable	NA
Confidentiality	#27	How personal information about potential and enrolled participants will be collected, shared, and maintained in order to protect	11

For peer review only

		confidentiality before, during, and after the trial	
Declaration of interests	#28	Financial and other competing interests for principal investigators for the overall trial and each study site	18
Data access	#29	Statement of who will have access to the final trial dataset, and disclosure of contractual agreements that limit such access for investigators	11
Ancillary and post trial care	#30	Provisions, if any, for ancillary and post-trial care, and for compensation to those who suffer harm from trial participation	11
Dissemination policy: trial results	#31a	Plans for investigators and sponsor to communicate trial results to participants, healthcare professionals, the public, and other relevant groups (eg, via publication, reporting in results databases, or other data sharing arrangements), including any publication restrictions	11
Dissemination policy: authorship	#31b	Authorship eligibility guidelines and any intended use of professional writers	NA
Dissemination policy: reproducible research	#31c	Plans, if any, for granting public access to the full protocol, participant-level dataset, and statistical code	11
**Appendices**			
Informed consent materials	#32	Model consent form and other related documentation given to participants and authorised surrogates	Supp. material-1
Biological specimens	#33	Plans for collection, laboratory evaluation, and storage of biological specimens for genetic or molecular analysis in the current trial and for future use in ancillary studies, if applicable	NA

The SPIRIT checklist is distributed under the terms of the Creative Commons Attribution License CC-BY-ND 3.0. This checklist was completed on 27. November 2020 using https://www.goodreports.org/, a tool made by the EQUATOR Network in collaboration with Penelope.ai

For peer review only
